# A stochastic programming approach to perform hospital capacity assessments

**DOI:** 10.1371/journal.pone.0287980

**Published:** 2023-11-09

**Authors:** Robert L. Burdett, Paul Corry, Belinda Spratt, David Cook, Prasad Yarlagadda

**Affiliations:** 1 School of Mathematical Sciences, Queensland University of Technology, Brisbane, Qld, Australia; 2 School of Mechanical, Medical & Process Engineering, Queensland University of Technology, Brisbane, Qld, Australia; 3 Princess Alexandra Hospital, Woolloongabba, Brisbane, Qld, Australia; Cyprus International University Faculty of Engineering: Uluslararasi Kibris Universitesi Muhendislik Fakultesi, TURKEY

## Abstract

This article introduces a bespoke risk averse stochastic programming approach for performing a strategic level assessment of hospital capacity (QAHC). We include stochastic treatment durations and length of stay in the analysis for the first time. To the best of our knowledge this is a new capability, not yet provided in the literature. Our stochastic programming approach identifies the maximum caseload that can be treated over a specified duration of time subject to a specified risk threshold in relation to temporary exceedances of capacity. Sample averaging techniques are applied to handle probabilistic constraints, but due to the size and complexity of the resultant mixed integer programming model, a novel two-stage hierarchical solution approach is needed. Our two-stage hierarchical solution approach is novel as it combines the application of a meta-heuristic with a binary search. It is also computationally fast. A case study of a large public hospital has been considered and extensive numerical tests have been undertaken to highlight the nuances and intricacies of the analysis. We conclude that the proposed approach is effective and can provide extra clarity and insights around hospital outputs. It provides a way to better calibrate hospitals and other health care infrastructure to future demands and challenges, like those created by the COVID pandemic.

## 1. Introduction

In this article a new approach for performing insightful and actionable quantitative assessments of hospital capacity is proposed and tested. Analysing hospital capacity and productivity is a difficult task because hospitals treat and care for a diverse cohort of patients with very different illnesses and conditions, with very different health care requirements. Also, there is significant competition for shared resources within hospitals, and how that competition is regulated greatly affects the assessment [1].

This contemporary topic has become noticeably more important over the last few years and the current COVID pandemic appears to be a significant catalyst. It has been reported in [2] that the health care needs created by the coronavirus pandemic far exceeds the capacity of most hospitals and has placed additional sustained demands on public health systems around the world. They also describe significant imbalances between the supply and demand for medical resources in many countries. As such, principal “bread and butter” services like elective surgery have been cancelled in great numbers, causing waiting lists and waiting times to increase. Besides pandemics and natural disasters (i.e., like earthquakes, tsunami, etc) our interest in this topic is motivated more generally by the inherently challenging capacity-related decisions relating to *prioritisation*, *allocation and sharing of resources* within a single hospital or across many, which hospital planners and executives have always faced [1, 3, 4]. These decisions have a profound and enduring impact on the output of a hospital, and of the health care system in general.

Given the immense number of hospitals in the world, there is a great need for practical decision support tools. In Australia alone (i.e., where this research originates), 693 public and 657 private hospitals are currently managed and operated. According to [5] these hospitals collectively operate about 62,000 beds in public and 34,000 in private facilities. In most countries the cost to provide medical and surgical care is increasing yearly. In Australia, this is also true. The AIHW reports that about 41% of public hospital funding and 24% of private, is provided by the Australian Government. In 2017–2018 for instance, there was recurrent expenditure of approximately AUD 71 billion on public hospitals.

Despite the great need for decision support tools, there appears to be a significant lack of them to implement and put into practice. Upon detailed investigation, there appears to be a specific lack of data-driven quantitative decision support tools to make well-informed capacity allocations or other capacity related decisions of a strategic or tactical nature in a single hospital, or across a regional healthcare system [3]. Of the existing decision support tools, anecdotal evidence suggests very few hospitals have adopted them for regular use. As [6] reports, current techniques are too limited, being too myopic, focussing only on the development of long-term cyclical plans, and are incapable of providing solutions for real-life sized instances. [7] also comments, integrated models that can tie together competing metrics in capacity planning decisions are not being developed. Nor are there tools that provide sufficient “what-if” capabilities to support managerial decision making.

### 1.1. Research aims and methodology

In this article we consider strategic level hospital capacity assessment and hospital resource allocation and integrate stochastic treatment durations and lengths of stay. To the best of our knowledge this is a new capability, not yet provided in the literature. It is necessary to point out that we do not explicitly consider how to measure quality of care or maximize health outcomes in our approach. Nor do we consider patient scheduling or other operational decision-making.

Apart from the development of analytical methods and algorithms, determining whether an adequate approach can be devised and put into practice is an important line of inquiry. The inclusion of stochastic durations seems like a necessity for any decision support tool that is planned for actual use in a hospital or health care environment. We also suspect that to be applied by hospital planners and executives, any assessment approach should be embedded within an appropriate decision support tool within an intelligent hospital information system.

As indicated by our study of the literature, a comprehensive stochastic QAHC has yet to be developed and tested, so one is proposed in this article. Based on our observations and consultations with hospital practitioners, we believe that there is a strong need for a stochastic QAHC to provide situational awareness around performance impacts of capacity-related decisions. For instance, like adding or removing beds in wards, building new theatres, changing the master surgical schedule (i.e., adding or removing sessions, changing session duration or sessions per day, changing the number of days theatres operate per week), treating new patient types, applying new medical or surgical techniques, etc. Our approach can also highlight the level of buffering needed to offset uncertainty in treatment times and length of stay. Without appropriate buffering, the variability of medical and surgical activity durations can greatly affect the output of a hospital and can create short-term oversaturation of resources, resulting in bottlenecks, overtime, staff fatigue, and reduced quality of care.

Our background research indicates that the deterministic mixed integer programming (MIP) model of [3] is a good starting point, however, significant modification is required to develop a more informative stochastic approach to capacity assessment. The new model identifies the maximum caseload achievable over time and optimally allocates resources’ capacity to perform various medical and surgical tasks. As such the model can be viewed as both a “capacity assessment” technique and as a “capacity allocation” technique. It is noteworthy to mention that the original model in [3], provided an accurate measure of theoretical capacity. With the incorporation of empirical durations, however, our new approach provides a more accurate measure of operational capacity.

### 1.2. Risk paradigm

The main objective of a QAHC is to determine what performance or output is possible from a hospital over time. To identify the maximum output, the predominant approach is to choose a patient caseload that saturates (i.e., fully utilizes) hospital resources. However, in practical situations, more time may be required to treat patients than expected, and hospital resources may exceed their allotted time availability. Hence, the risk of exceeding the hospital resources time availability should be managed. In a deterministic model, this is quite straight forward. A linear constraint of the form ∑_*a*∈*A*_*n*_*a*_*t*_*a*_≤*T* can be imposed for each resource present. Here, *A* is the set of activities, *t*_*a*_ is the deterministic time of activity *a*, *n*_*a*_ is the number of activities of type *a* assigned (i.e., a decision), and *t_a_* is the time availability of the resource. In a stochastic situation, however, the simple linear constraint shown is no longer sufficient, as activity durations are random variables. As such, the value of the left-hand side depends on the realisation of each occurrence of an activity and changes every time it is evaluated. We hereby denote $t_{a}^{s}$ as the *s*^th^ realisation of activity *a*.

There are various mathematical frameworks that can be applied for stochastic scenarios like this. To provide a compromise between good system performance and satisfying random constraints [8], the chance-constrained programming (CCP) paradigm [9–12] is adopted. Hence, to ensure the probability of exceeding resource capacity during a given time horizon remains below a certain threshold, constraints of the following form Pr(∑_*a*∈*A*_*n*_*a*_*t*_*a*_>*T*)≤1−*α* are added for each hospital resource. Constraints of this nature provide a suitable risk-management framework for hospital managers and executives, ensuring sufficient buffering in the system to account for variabilities between patients. In practice it is important to restrict the extent of the over usage because staff are required to perform overtime in those circumstances. This causes increased staffing costs and burnout. It is worth noting that reliable shift completion is important to staff morale and retention, and patient safety is tied to the frequency and length of overtime in ORs [9]. This approach can be risk averse and aligns with the conservative policies and practices of many hospitals, that are needed to maintain the highest quality of care. It also provides modelling flexibility to deal with reliability issues [13]. The main contributions of this article are as follows:

- Development of the first pragmatic strategic level capacity allocation approach to enable quantitative assessments of hospital capacity considering uncertain and generally distributed patient treatment durations and length of stay.
- Formulation of a stochastic hospital capacity assessment model (SHCAM) and the creation of a deterministic equivalent model using sample-averaging techniques.
- Development of a novel two-stage hierarchical solution algorithm for solving the proposed SHCAM, involving the application of a binary search algorithm with an embedded meta-heuristic.
- Application of the two-stage hierarchical solution algorithm to a real-life public hospital of large size and a sensitivity analysis of risk of resource over-usage.
- Managerial insights regarding the application of the proposed SHCA approach to real hospitals.

The format of this article is as follows. In Section 2, previous research is discussed and analysed in more detail. In Section 3, the SHCAM is introduced, followed by details of the two-stage hierarchical solution algorithm. In Section 4, the SHCA approach is applied to a real-world scenario. Conclusions, managerial insights, and other discussions are presented last, in Section 5.

## 2. Literature review

*Capacity assessment*, *capacity allocation* and *capacity planning* are the focus of this article. These are fundamental topics in Industrial Engineering, Operations Research and Management Science. Although well researched “generally”, articles on this topic are relatively sparse in the health care area [7]. This contrasts greatly with more popular areas like transportation and manufacturing; see for example: [14–20]. Over the last decade there has been significantly more research on hospital management, capacity planning, capacity allocation and optimization. This has resulted in the development of some well-documented and well-tested quantitative approaches. In the following discussion, important articles in the field are examined. First deterministic approaches are considered and then stochastic.

### 2.1. Hospital capacity planning with no consideration of uncertainty

The article by [21] is noteworthy because a “state-wide” hospital capacity assessment approach was proposed. In their data envelopment analysis (DEA) approach the capacity metric is consistent with engineering practices and is the maximum rate of output per unit of time. To apply their DEA approach, each hospital must be assessed in terms of specialty capacity and general capacity. Information on hospital capacity, patient characteristics, inpatient discharges, and financial performance are also included to perform their articles’ study. Despite their approaches’ applicability, their DEA approach was developed to facilitate emergency preparedness planning only and no other testing of a more generic nature was performed. Their metric of output is not the same metric considered in this articles’ approach, nor do they appear to include any stochasticity.

[22] put forth the idea of a smart hospital and health care environment and considered patient flow scheduling and capacity planning. In their viewpoint, “a smart environment is one that is designed to facilitate people’s experience that includes a set of devices and many intelligent supporting techniques”. In response, a quantitative analysis and a dynamic scheduling policy are proposed. A formal algebraic modelling approach, an ODE based fluid flow analysis and simulation tools were implemented. Despite the general potential of their approach, it has only been applied to a rheumatology department and not to an entire hospital.

[3] put forth the idea of a holistic hospital capacity assessment approach. They provided a deterministic mixed integer programming (MIP) model to perform a strategic capacity assessment of an entire hospital. Their model determines the maximum number of patients that can be treated within a given duration of time subject to various technical constraints. The model optimally allocates resources to surgical and medical tasks within the defined patient care pathways provided. Other capacity querying activities are also discussed and facilitated by the model. [1] also introduced a multi-objective approach for hospital capacity assessment. Their proposed multi-objective hospital capacity allocation model (MOHCM) identifies non-dominated capacity allocation solutions and provides a sensitivity analysis of patient case mix and the effect upon hospital output. In their numerical testing, 21 objectives were considered, one for each surgical specialty in a local hospital.

An optimization approach for outpatient capacity allocation planning was developed in [7]. Their approach performs planning through the optimization of an appointment template and reserves appointment slots to limit access delays. Each slot may have a deterministic duration that depends on the type of appointment it is designated for. The proposed “templating” process has been performed separately in a reactive manner in past research, but in their article, an integrative, proactive approach is suggested.

A non-linear mixed-integer programming model for case mix planning was developed in [23]. Their model incorporates economies of scale and investigates the effect of changes in the efficiency of resource use on the optimal case mix. As their model is non-linear, piecewise linear functions were used, and an iterative approximation scheme (i.e., e-optimal solution methodology) was applied. They conclude that meaningful results depend upon the accuracy of input parameters. Demand, for instance, is difficult to obtain. They, however, omitted uncertainty to keep their computations tractable.

### 2.2. Hospital capacity planning with consideration of uncertainty

Stochastic techniques have been applied for a variety of planning problem in health. The types of stochastic problems that can be solved efficiently, however, are still limited [13]. [24] proposed an optimization model to locate hospitals and their capacities for an upcoming disaster, like an earthquake. Objectives of the decision making were distance travelled and coverage. For small instances, they reported the successful application of an MIP solver (namely IBM ILOG CPLEX). For larger instances, however, a Simulated Annealing (SA) meta-heuristic was necessary. They also considered how to readjust hospital capacities. A similar problem was also tackled recently in [25]. They considered the allocation of hospital beds to cities and proposed a simulation-optimisation approach to solve the facility location-allocation decision problem. Distinct patient arrival rates and length of stay were considered, and a discrete event simulation was proposed to evaluate two conflicting objective functions.

Hospital bed planning (HBP) was considered in [26] and a multi-objective stochastic MIP to assign beds to hospital departments was proposed. To solve their model, they used chance-constraints and stochastic programming with recourse. They also integrated goal programming to handle three objectives. Their approach considers regions with different hospital types, with a fixed number of departments, each containing several specialists. They used nurse-to-bed and doctor-to-bed ratio data. The sample average approximation (SAA) approach was applied in [27] to solve a stochastic planning model. They considered the selection of a case mix for a single surgical department, with uncertain surgery durations, length of stay and demand. The number of theatre hours assigned to each patient group was a primary decision.

The capacity allocation of hospital wards and the joint optimization of hospital revenue and equity among different types of patients was considered in [28]. In response they proposed a multi-objective stochastic programming model with two objectives. As their objective functions have no “closed form” they used a data-driven discrete-event simulation to evaluate random patient arrivals and lengths of stay. An adaptive e-constraint algorithm and a multi-objective Genetic algorithm were developed to solve the proposed non-linear mathematical model.

Capacity balancing to reduce overcrowding of hospital wards was considered in [29]. They developed mathematical models to minimize the number of rejections at preferred wards, by changing the distribution of bed resources. In their article, patient flow is modelled using a homogenous continuous time Markov chain model.

Case mix planning [4] was considered in [30] and a multi-phase approach was developed to generate a set of candidate solutions. They applied simulation techniques to evaluate the master surgical schedule (MSS) and each case mix solution. A framework for evaluating the effect of stochastic parameters on the case mix of a hospital was presented in [31]. Stochastic influences were categorized relative to demand, resource consumption, and resource availability. Numerical testing was then performed for different options.

### 2.3. Hospital scheduling with stochastic durations

Stochasticity has been addressed more frequently in elective and outpatient patient scheduling, and as such is useful as a guideline to address stochasticity in case mix planning and hospital capacity allocation. Elective patient scheduling, with uncertain surgery and length of stay (LOS) and with consideration of upstream and downstream activities (like pre- and post-operative care), have been researched by [32–42]. In [36] several stochastic optimization models were developed, and SAA was applied as a solution method. The SAA approach is shown to *outperform* the expected value problem. Downstream capacity was considered in [37] and a two-stage “robust” optimization approach was proposed. Specifically, they applied column and constraint generation methods to obtain exact solutions. A simulation model is employed to calculate the proposed risk measures. [32] considered theatre planning and developed a “two-stage chance-constrained” stochastic programming model with a cost objective. They considered stochastic surgery durations and lengths of stay in an ICU. They solved this model using SAA. Their numerical testing shows that robustness is only achieved with high cost and low operating room utilization. Uncertain emergency arrivals are later incorporated in [33].

[9] considered surgery planning and scheduling when there is large uncertainty in surgery durations. They developed a stochastic model to minimize cost of opening an operating room subject to CC limiting overtime. They assigned OR to surgeries and developed schedules of surgeries using a two-stage process and branch and cut methods.

[35] proposed an MIP model with three objectives, integrated as a weighted sum. They proposed a robust counterpart, and this was solved using a local neighbourhood search heuristic. [41] proposed a two-level optimization model to address the weakness of a conventional formulation, that optimizes the schedule of one single decision period, without consideration of later periods. The SAA approach was also applied. [40] developed a distributionally robust model (DRM) for surgery block allocation with a cost objective. Their problem involves assigning surgeries to theatres and is designed to deal with surgery duration uncertainty. Since their model is intractable, several reformulations were proposed. It is worth pointing out that DRM is an alternative modelling paradigm for traditional stochastic programming. The objective is to find decisions that minimize the worst-case expected cost.

[38] presented an optimization model for a stochastic single-resource sequencing and scheduling problem encountered in outpatient scheduling. A weighted sum objective is posed, that aggregates waiting time, idle time, and clinic overtime. To incorporate procedure duration uncertainty, a sample average approximation is used. [39] developed a “distributionally robust optimization” approach for outpatient colonoscopy scheduling. An optimal appointment sequence and schedule is obtained. In their approach they minimize the worst-case weighted expected sum of patient waiting, provider idling, and provider overtime. They incorporate pre-operative “preparation” activities, recognizing the impact and effect on the variability in colonoscopy duration. [42] developed a column-generation-based heuristic approach integrated with SAA for the weekly “stochastic” surgery scheduling problem, with downstream capacity constraints.

[34] developed for the daily scheduling of surgical patients, an integrated scheduling and capacity planning approach. They declare that “traditional scheduling policy, driven by operating room usage, may lead to significantly suboptimal use of downstream capacity and may result in up to a three-fold increase in total expenses”. In contrast, “a scheduling policy based on downstream capacity usage often performs close to an integrated scheduling policy, and therefore may serve as a simple, effective scheduling heuristic for hospital managers—especially when the downstream capacity is costly and less flexible”.

### 2.4. Solution techniques for Chance Constrained Problems (CCP)

Chance-constrained stochastic programs are inherently difficult to solve except for some special cases [43]. They are strongly NP-hard and relevant MIP formulations have a weak LP relaxation [44]. The literature describes various exact and approximate methods to solve CCP. Stochastic programs and CCP are predominantly solved by relying on an approximating problem obtained via discretization of the probability space [43]. Sample Average Approximation is the most popular method and has been used to solve many models with uncertain parameters. Scenario-based approaches do not require restrictive assumptions on the distribution of the random parameters, which increases their generality and flexibility [45]. They do, however, rely upon the generation and evaluation of a representative set of scenarios [10–12]. SAA is advantageous in cases where the expected outcomes can be observed and measured, such as risk and resource over (under) usage. Upfront, SAA, has a few known weaknesses. First, the SAA makes a solution robust to a subset of possibilities, rather than to all possibilities. An increase in the number of scenarios leads to an increase in the accuracy of the method, however, computational requirements will also increase significantly [10]. Recently constraint removal has been suggested as a means for achieving high-quality, non-conservative solutions. [43] proposed a heuristic approach based upon Lagrangian relaxation and scenario decomposition. [46], studied the link between a CCP and its sample counterpart. They considered trading feasibility for performance by permitting the violation of some of the chance constraints (CC) (i.e., they remove *k* out of *N* chance constraints). [45] considered CCP and tested the tractability of constraint removal approaches for obtaining high-quality solutions to portfolio optimization problem. In their solution approach multiple convex optimization problems are solved. [47] introduced a similarly inspired approach, called the Pool&Discard algorithm for handling chance constrained portfolio optimization problems. Their approach utilizes the warm-start functionality of modern solvers and involves the iterative solution of smaller sub problems. The most violated constraints are added to the relaxed problem after each solve, and the process is repeated until a termination criterion is met.

[44] focused on MILP based approaches. They discussed various strategies from the literature such as improving relaxation bounds, constructing approximate solutions, coefficient tightening of big-M constraints and so forth. Practical application of the considered methods, however, was not considered.

The replacement of single CCs with a single joint chance constraint (JCC) is another approach that has been considered. With this alternative modelling paradigm, the probability level of meeting all restrictions is simultaneously met. Results in recent papers show that methods for handling JCC are sometimes computationally faster and are more efficient at finding feasible solutions. [48] solved a stochastic optimization problem in chemical processing and developed an approximate method to handle JCC. [49] considered a single-item single-resource capacitated lot-sizing problem with stochastic demand. They proposed a JCC in which the probability that an inventory shortage occurs during the planning horizon is limited to a maximum acceptable risk level. An approximate solution method based upon sample approximation was applied. [50] applied JCC in a power flow optimization problem in electrical engineering. [51] applied JCC to two simple stochastic models of a generic nature. [8] considered a stochastic gas network design problem with JCC. The proposed solution method introduces auxiliary binary variables, relaxes them into continuous variables, and regularizes the resulting optimization problem. To handle a greater number of scenarios, the regularized problem was transformed into a two-stage problem. [52] considered a multi-period single-product inventory problem with demand uncertainties. They formulated a model with JCC and transformed it into a linear programming model using an approximation for the JCC.

### 2.5. Findings

As demonstrated, there are stochastic models for many decision problems arising in health care. Most, however, are only applied to one part of the hospital. Another observation is that stochastic techniques are more often applied to short term “operational” planning problems, like theatre scheduling. Application to strategic problems as considered in this article, is less common. It is clear from the literature that smaller instances of stochastic problems are significantly more tractable and, “even crude discretization’s of the random parameters lead to an exponential growth in computational complexity” [41].

Our literature review shows that there are few if any competing approaches for the decision-making problem that we have tackled in this article. Existing case mix planning approaches primarily provide a single solution, that excludes uncertain patient arrivals and operation times [30]. The uncertain arrival of patients requiring urgent care is also excluded.

Various stochastic features have been included in capacity allocation problems [31]. Despite this, detailed practical examples applying those methods are rare. Uncertain durations are arguably and anecdotally more important than other stochastic features, particularly when it comes to elective surgeries. They pose the most difficult barrier to planning and cause the greatest variations in hospital outputs. As such, we concentrate on that feature more comprehensively and solve practical instances arising in a larger sized hospital.

The literature demonstrates that sample averaging techniques and discrete event simulation are well tried and tested. They appear popular for stochastic optimization as classical robust optimization may yield conservative solutions [40]. Including stochastic parameters in strategic capacity allocation, however, could be considerably more difficult. There is evidence to suggest that SAA is easier to apply to scheduling problems. When solving a stochastic scheduling problem, the number of activities is static, and for each scenario a duration is selected for each activity. In strategic capacity allocation, the number of activities and patients treated of each type is selectable. So, the generation of scenarios will in theory be more complex. This will be investigated further.

The nature and number of the chance constraints, considered in the literature varies. It is quite apparent that portfolio selection is popular as a benchmark problem to test new stochastic programming approaches [53]. There is, however, only one chance constraint in that decision problem and a known set of assets (a.k.a., investments) to generate scenarios for. In our HCAP there is one chance constraint for each treatment space and treatment area in every scenario. This is appropriate in the context of hospitals in which specialties and treatment spaces locally have capability to flex and smooth out workload in the short term.

In recent engineering applications JCC are imposed, and new approximate methods have been developed. Reductions in computational effort are described, however, as a modelling approach, we found no evidence of any superiority. [50] comment that there are difficulties in handling JCC, requiring computationally heavy sampling-based approaches which are limited by problem size.

Many approaches in the literature assume the presence of historical data concerning length of stay and treatment duration. If that is not present, those approaches are of dubious value. Possibilistic programming approaches involving fuzzy numbers may be a good option for that situation [54] or distributionally robust optimization [53].

### 3. The stochastic hospital capacity allocation model

In this section, the proposed stochastic hospital capacity allocation model (SHCAM) is discussed, and following this, an approach to solve it is presented. The terminology used, however, is first introduced. To avoid confusion, it is important to note that in *some limited circumstances we reuse some symbols and differentiate parameters by their index*. This abuse of notation is beneficial as fewer symbols need to be introduced and understood. A clarifying example, for instance, is the designation of three similar set variables $Z_{a}^{1},Z_{a,b}^{2},Z_{c}^{3}$ as simply *Z*_*a*_,*Z*_*a*,*b*_, *Z*_*c*_. For quick reference, a full list of all notations can be found in Appendix A in S1 File.

### 3.1. Parameters

For the proposed QAHC it is necessary to input the hospitals’ structure and some other relevant parameters. As hospitals are deemed to be a collection of treatment areas partitioned into treatment spaces [3], it is necessary to input the set of areas and spaces, denoted respectively by *W* and *S*. Each area *w*∈*W* has a set of treatment spaces which must also be input, denoted by *S*_*w*_.

It is noteworthy to mention that the beds and operating theatres constitute the main treatment spaces of the hospital. These exist, within only one area.

To treat patients, hospitals have an assortment of medical and surgical units staffed by a variety of doctors, nurses, and other health care professionals. Each hospital unit *u*∈*U* provides treatments for a specific medical or surgical specialisation (a.k.a., a specialty). There are a variety of patient types (a.k.a., groups) treated by hospitals. For each patient type *g*∈*G*, different patient sub types exist. Each one of those requires a specific patient pathway (a.k.a., patient care plan). The exact nature of these pathways is inherently linked to a patient’s condition, diagnosis, and the planned medical and surgical procedures. The set of pathways for patient type *g* is hereby denoted *P*_*g*_. Each pathway *p*∈*P*_*g*_ contains a set of activities, denoted *A*_*g*,*p*_. The number of activities is denoted *K*_*g*,*p*_. For each activity *a*∈*A*, where $A={⋃}_{\forall (g,p)\in GP}A_{g,p}$ and $GP=\{(g,p)|\forall g\in G,\forall p\in P_{g}\}$, there is a defined duration, unit, and activity type. These are denoted as follows, *t*_*a*_, *u*_*a*_, *ϕ*_*a*_. Some typical activity types are acute care (ac), imaging (im), intensive care (ic), medical care, post anaesthesia care (pac), pre and post operative care (postop), palliative care (pal), radiology (rad), and rehabilitation (reh).

Activities can be performed in different locations within the hospital. The activities that can be performed in hospital space *s* are inferred as *A*_*s*_ = {*a*∈*A*|*s*∈*S*_*a*_} where *S*_*a*_ is the set of spaces that may be used for activity *a*. The activities that can be performed in area *w* is *A*_*w*_ = {*a*∈*A*|*w*∈*W*_*a*_} where *W*_*a*_ is set of areas that can be used for activity *a*. It is worth pointing out that *S*_*a*_ and *W*_*a*_ can be input directly or inferred using the activity type, and or the specialty unit involved. Further details of these pragmatic considerations could be discussed at length but are omitted for brevity.

The patient case mix (i.e., the composition and volume of patients to be treated) of a hospital, has a large impact on resource utilization. In this article, two case mix are provided as input to the analysis. The first specifies the proportional mix of patient types, and the second defines the proportional mix of patient sub types. These are defined respectively by $\mu_{g}^{1}$ and $\mu_{g,p}^{2}$. It is worth noting that $\sum_{\forall g\in G}\mu_{g}^{1}=1$ and $\sum_{p\in P_{g}}\mu_{g,p}^{2}=1\;\forall g\in G$.

### 3.2. Stochastic hospital capacity allocation model

The purpose of the proposed SHCAM is to identify for a specified duration of time *T* the maximum number of patients that can be reliably treated, denoted N. This answer may also be regarded as a rate of output. The duration for the analysis may be short or long. A shorter time frame is suggested when handling seasonal demands. Seasonal demands can cause vastly different patient case mix throughout the year. If patient case mix is consistent, however, we are unaware of any reason for not considering a longer time frame.

The level of reliability to be imposed is a parameter. It will be defined by the user and enforced using CC. The concept of CCs is well tested in the literature. They have been shown on many occasions to be an appropriate mechanism for evaluating complex probabilistic constraints. This approach is also arguably easier to understand, and communicate to hospital managers and executives, in a position to consider the application of this articles’ model.

The number of patients of each type to be treated is denoted by $n_{g}^{1}$ and how many are of a specific sub type is denoted $n_{g,p}^{2}$. This is the caseload for the hospital. Both decision variables are assumed real-valued, but they may also be restricted to be integers, if circumstances require it. Non-integer values of these decisions highlight that some patients will be partially and not fully treated within the period. As such, their treatment would complete in the next period. The rationale for real valued decisions was established previously in [3].

The case mix proportions are a key aspect. Without them, any analysis would be highly biased, and patient types with short treatment durations will be chosen. It is important to recognize that $N=\sum_{g\in G}n_{g}^{1},n_{g}^{1}=\mu_{g}^{1}N\;\forall g\in G$ and $n_{g,p}^{2}=\mu_{g,p}^{2}n_{g}^{1}\;\forall g\in G,\forall p\in P_{g}$. Also, $n_{g}^{1}=\sum_{p\in P_{g}}\left(n_{g,p}^{2}\right)\;\forall g\in G$. The number of activities of each type *a* is also a dependent decision variable. It is hereby denoted $n_{a}^{3}$. As each pathway has multiple activities, it is necessary that $n_{a}^{3}=n_{g,p}^{2}\;\forall a\in A_{g,p}$.

Every activity occurs in an assigned treatment space within an assigned treatment area. All treatment spaces, however, have a time availability *T*_*s*_≤*T*. The number of activities of type *a* performed in space *s* is a primary decision and is denoted *β*_*a*,*s*_. This is called “*the allocation*”. It may also be viewed as either integer or real-valued. Some assignments are inherently invalid. It is necessary to set *β*_*a*,*s*_ = 0 ∀*a*∈*A*, ∀*s*∈*S*\*S*_*a*_. As such the model can only assign tasks to the correct hospital spaces in the correct hospital areas. If other spaces become available (unavailable), then they should be added (removed) from set *S*_*a*_ before the model is solved. The SHCAM can now be presented simply as follows:

MaximizeN[Totalnumberofpatientstreated] (1)

Subject To

$$\sum_{s\in S_{a}}\beta_{a,s}=\mu_{g,p}^{2}\mu_{g}^{1}N\;\forall (g,p)\in GP,\forall a\in A_{g,p}\;[Required\;allocations]$$ (2)

$$Pr(U_{s}\leq T_{s})\geq {SL}_{s}\forall s\in S\;[Service\;(a.k.a.,reliability)\;level]$$

$$\mathrm{where}\;U_{s}=\sum_{\forall a\in A_{s}}F(\beta_{a,s},t_{a})\;[Treatment\;space\;occupancy]$$ (3)

$$\beta_{a,s}\geq 0\;\forall a\in A,\forall s\in S_{a}\;[Positivity\;requirement]$$ (4)

This model is a stochastic version of the deterministic capacity allocation model from [3]. It has ∑_∀*a*∈*A*_*S*_*a*_ positivity constraints, $\sum_{\forall (g,p)\in GP}\left|A_{g,p}\right|$ resource allocation balance constraints and |*S*| reliability constraints. Constraint (3) is the chance constraint, mentioned earlier. It ensures that the solution is sufficiently reliable. It describes the usage of each treatment space given uncertain activity durations and replaces the deterministic version, *U*_*s*_≤*T*_*s*_ ∀*s*∈*S* previously applied in [1, 3]. The probability on the left-hand side is just a count of how often over usages would occur. Some over usages would be fine, but the exact level a hospital would be willing to tolerate must be explicitly defined for the analysis to provide, a correct assessment. The term Pr(*U*_*s*_≤*T*_*s*_) is hereby described as the service (a.k.a., reliability, confidence) level, for treatment space *s*. It can take any value between zero and one, i.e. ${SL}_{s}\in [0,1]$. The risk of exceeding the time availability of treatment space *s*∈*S* may alternatively be computed and restricted, i.e., $Pr(U_{s}>T_{s})<1-{SL}_{s}$ where the level of risk (a.k.a. the risk threshold) is ${RL}_{s}=1-{SL}_{s}$.

It is worth mentioning that we choose the constraint $Pr(U_{s}\leq T_{s})\geq {SL}_{s}$, rather than *E*[*U*_*s*_]≤*T*_*s*_, as the later may cause too many over-usages in practice. This occurs when there is large variability in the treatment times of activities. From the data we have collected from local hospitals, we have observed very large tails in the empirical distributions of all the medical and surgical procedures performed.

An alternative version of the SHCAM is also suggested for practical applications. In that version the assignment of activities to treatment areas is considered rather than to specific treatment spaces. In this variant, all constraints involving *β*_*a*,*s*_ are replaced with *α*_*a*,*w*_, the number of activities *a* assigned to treatment area *w*. The revisions necessary to the model are as follows:

$$\sum_{w\in W_{a}}\alpha_{a,w}=\mu_{g,p}^{2}\mu_{g}^{1}N\;\forall a\in A\;[Allocations\;required]$$ (5)

$$\alpha_{a,w}\geq 0\;\forall a\in A,\forall w\in W_{a}\;[Positivity\;requirement]$$ (6)

$$\alpha_{a,w}=0\;\forall a\in A,\forall w\in W\backslash W_{a}\;[No\;allocation\;permitted]$$ (7)

$$Pr(U_{w}\leq T_{w}|S_{w}|)\geq {SL}_{w}\forall w\in W\;[Service\;level\;requirement]$$

$$where\;U_{w}=\sum_{\forall a\in A_{w}}F(\alpha_{a,w},t_{a})\;[Treatment\;area\;utilization]$$ (8)

This formulation has vastly fewer decision variables and is well suited to what-if scenarios where |*S*_*w*_| is a decision variable. It would, however, be inapplicable if we were to replace *t*_*a*_ with a space dependent duration *t*_*a*,*s*_.

### 3.3. Pragmatic considerations

Patient type and pathway modelling are a key ingredient, necessary prior to the application of the SHCAM. For deterministic situations, upper bounds can be pre-computed for the main decision variables, i.e., $n_{g}^{1}\leq {\hat{n}}_{g}^{1},n_{g,p}^{2}\leq {\hat{n}}_{g,p}^{2}$ and $\beta_{a,s}\leq {\hat{\beta }}_{a,s}$. These bounds are derived in Appendix B in S1 File.

The afore-mentioned reliability level is a selectable parameter, but various values need to be evaluated to gain a complete picture of the hospitals’ capacity and to gain the most insights. A single value could be chosen; the value a hospital might choose is specific to their “appetite for risk” and/or their risk threshold. In the real-world, increased appetite for risk directly translates into a need to perform overtime in theatre sessions, managing staff fatigue due to overtime, and managing the frequency of patients being cared for in wards outside of their required specialty. The risk threshold is a subjective value, and it may require some experimentation to choose. We envisage an iterative process is perhaps warranted, in which the modification of the risk threshold is a key step. For instance, the planner would initially propose (or be given) a threshold of risk and then perform a QAHC. After evaluation of the results, the threshold may be incrementally increased or decreased and further assessments performed, until a desired outcome is reached.

Constraint (3) is a generic probabilistic statement and must be converted to a proper constraint.

This is not straightforward because *U*_*s*_ is a function of different *β*_*a*,*s*_ values which are unknown. To evaluate the usage (a.k.a., occupancy) *U*_*s*_ it is necessary to choose a realisation of the random variables given by *t*_*a*_. Function F describes how much time is required to process *β*_*a*,*s*_ activities. In deterministic situations $F(\beta_{a,s},t_{a})=\beta_{a,s}\times E[t_{a}]$, however, in other circumstances, a general analytical expression does not exist. As each activity may have a different statistical distribution, it is not possible to form a single distribution (i.e., convolution) for *U*_*s*_, except for a few special situations. One of those is where every activity is normally distributed. In that situation, the convolution of several normally distributed random variables also produces a normal distribution. A cumulative density function (CDF) for *U*_*s*_ can be created (denoted $F_{U_{s}}$) and the inverse CDF can be evaluated. A proper constraint can be derived and added to the model, i.e., $Pr(U_{s}\leq T_{s})\geq {SL}_{s}\Rightarrow F_{U_{s}}(T_{s})\geq {SL}_{s}\Rightarrow F_{U_{s}}^{-1}({SL}_{s})\leq T_{s}$. Another similar situation occurs when every activity duration is uniformly distributed. An Irwin-Hall distribution results.

Another possibility worth considering is how to restrict the extent of the over usage likely to occur, rather than the number of times over-usage occurs. This may be achieved by restricting the expected over utilization, $E[O_{s}]\leq O_{s}^{max}$ where $O_{s}^{max}$ is the maximum over utilization permitted. Computing *E*[*O*_*s*_], is again problematic as *O*_*s*_ is an unknown random variable.

The buffering required on each treatment space, hereby denoted *B*_*s*_, is an important piece of “administrative” information worth computing and reporting to hospital managers and executives. For a deterministic situation it can be computed in the following way:

$$B_{s}=T_{s}-E[U_{s}]\;where\;E[U_{s}]=\sum_{\forall a\in A_{s}}\beta_{a,s}E[t_{a}]$$ (9)

The percentage utilization and percentage “slackness” (a.k.a., level of buffering) can then be computed as follows:

$$\rho_{s}^{util}=100\times E[U_{s}]/T_{s}\;\mathrm{and}\;\rho_{s}^{slack}=100\times B_{s}/T_{s}$$ (10)

### 3.4. Deterministic equivalent model

In this section a deterministic equivalent model is developed for the SHCAM of Section 3.2. It is hereby labelled SHCAM-SAA because it is based upon the application of the sample average approximation paradigm. The earlier model is preserved but some additional variables and constraints are added. These are necessary to evaluate probabilistic constraint shown in Eq (3).

At first glance, the hospital capacity allocation problem is not well suited to the SAA approach. Each patient type and path may occur numerous times. Hence, the exact number of values to generate (a.k.a., the sample) is variable. This makes scenario generation more difficult. In other decision problems that is not the case, for instance, in scheduling, the exact number of jobs and activities that are to be scheduled, each having stochastic durations, is known upfront. As such, each scenario involves the generation of a specific number of realisations of the durations.

When generating a representative set of scenarios for our stochastic QAHC, it is necessary to provide a sample duration for each realisation of each activity on each treatment space that could be used. Parameter $t_{a,s}^{i,n}$ (a.k.a., $t_{a,s}^{i}[n]$) is designated and denotes the *n*th realisation of activity *a*∈*A* in scenario *i*∈*I*, on space *s*∈*S*_*a*_, where *n*∈{1..*β*_*a*,*s*_}. We could also define parameter $t_{a}^{i,n}$ which has fewer values, but that would require us to use the same values for each space (i.e., $t_{a,s}^{i,n}=t_{a}^{i,n}\;\forall s\in S_{a}$) or else force us to select a sub set of values for a particular space, which would be unnecessary.

Given our assumptions, the utilization level of each space is the sum of *β*_*a*,*s*_ realisations. It can be computed by Eq (11) if *β*_*a*,*s*_ is an integer or by Eq (12) otherwise.


$$U_{s}^{i}=\sum_{\forall a\in A_{s}}\left(\sum_{n=1,\ldots ,\beta_{a,s}}t_{a,s}^{i,n}\right)\;\forall s\in S,\forall i\in I$$ (11)

$$U_{s}^{i}=\sum_{\forall a\in A_{s}}\left(\sum_{n=1,\ldots ,\lfloor \beta_{a,s}\rfloor }t_{a,s}^{i,n}+(\beta_{a,s}-\lfloor \beta_{a,s}\rfloor )t_{a,s}^{i,\bar{n}}\right)\;\forall s\in S,\forall i\in I\;\mathrm{where}\;\bar{n}=\lceil \beta_{a,s}\rceil$$ (12)

Eqs (11) and (12) are a starting point but must be reposed as *β*_*a*,*s*_ is a decision variable. For cases where *β*_*a*,*s*_ is an integer, the following equations are necessary:

$$U_{s}^{i}=\sum_{\forall a\in A_{s}}\sum_{n=1,\ldots ,{\hat{\beta }}_{a,s}}x_{a,s}^{n}t_{a,s}^{i,n}\;\forall s\in S,\forall i\in I$$ (13)

$$n\geq \beta_{a,s}+\epsilon -x_{a,s}^{n}{\hat{\beta }}_{a,s}\;\forall a\in A,\forall s\in S_{a},\forall n\in \{1..{\hat{\beta }}_{a,s}\}$$ (14)

$$n\leq \beta_{a,s}+(1-x_{a,s}^{n}){\hat{\beta }}_{a,s}\forall a\in A,\forall s\in S_{a},\forall n\in \{1..{\hat{\beta }}_{a,s}\}$$ (15)

$$x_{a,s}^{n}\in \{0,1\}\;\forall a\in A,\forall s\in S_{a},\forall n\in \{1..{\hat{\beta }}_{a,s}\}$$ (16)

Here, *ϵ* is introduced as a small value and $x_{a,s}^{n}$ is a binary indicator variable. It is used to turn on or off certain values of $t_{a,s}^{i,n}$. Constraint (14) and (15) respectively are used for setting $x_{a,s}^{n}=1$ if *n*≤*β*_*a*,*s*_ and $x_{a,s}^{n}=0$ if *n*≥*β*_*a*,*s*_+1. If *β*_*a*,*s*_ is real-valued, then Eq (13) is in principle still valid, however, we must add another clause for $x_{a,s}^{n}$ as follows: $x_{a,s}^{n}=\beta_{a,s}-\lfloor \beta_{a,s}\rfloor$ if *β*_*a*,*s*_<*n*<*β*_*a*,*s*_+1. The term ⌊*β*_*a*,*s*_⌋ can be replaced with an integer variable *y*_*a*,*s*_∈ℤ and additional constraints to compute it, namely *y*_*a*,*s*_≤*β*_*a*,*s*_ and *y*_*a*,*s*_+1≥*β*_*a*,*s*_+*ϵ*.

**Example**: If *β*_*a*,*s*_ = 4.2_._ Then $x_{a,s}^{n}=1$ for *n*≤4, $x_{a,s}^{n}=0.2$ for *n* = 5, and $x_{a,s}^{n}=0$ for *n*≥5.2.

Now let us define $e_{s}^{i}$ as an indicator of a scenario *i* exceeding the time availability of resource *s*. This variable is zero if the time availability of space *s* is not violated (i.e., $0\leq U_{s}^{i}\leq T_{s}$), and one otherwise (i.e., $T_{s}+\delta \leq U_{s}^{i}\leq U_{s}^{+}$) where *δ* is a small value used as a tolerance to approximate a strictly greater than constraint, and $U_{s}^{+}$ is the maximum over usage permitted. This indicator variable can be defined by the following constraints:

$$U_{s}^{i}\leq T_{s}+e_{s}^{i}(U_{s}^{+}-T_{s})\forall s\in S,\forall i\in I\;[U_{s}^{i}\leq T_{s}\;\mathrm{or}\;U_{s}^{i}\leq U_{s}^{+}]$$ (17)

$$U_{s}^{i}\geq e_{s}^{i}(T_{s}+\delta )\forall s\in S,\forall i\in I\;[U_{s}^{i}\geq 0\;or\;U_{s}^{i}\geq T_{s}+\delta ]$$ (18)

$$e_{s}^{i}\in \{0,1\}\forall s\in S,\forall i\in I\;[Binary\;domain\;constraint]$$ (19)

To manage the risk of over utilization, Constraint (3) can now be replaced with Constraint (20).


$$\sum_{i\in I}e_{s}^{i}\leq (1-{SL}_{s})|I|\;\forall s\in S$$ (20)

The left-hand side is the number of scenarios that exceed the time availability of space *s*. The probabilities of under and over utilization, however, can be explicitly computed as follows:

$$Pr(U_{s}\leq T_{s})=1-\frac{\sum_{i\in I}e_{s}^{i}}{\left|I\right|}\geq {SL}_{s}\;and\;Pr(U_{s}>T_{s})=\frac{\sum_{i\in I}e_{s}^{i}}{\left|I\right|}\leq 1-{SL}_{s}.$$ (21)

It is also worth computing the expected usage and over-usage using Eqs (22) and (23):

$$E[U_{s}]=\sum_{i\in I}U_{s}^{i}/|I|\;\forall s\in S$$ (22)

$$E[O_{s}]=\left\{ \begin{array}{cc} \frac{\sum_{i\in I}O_{s}^{i}}{\sum_{i\in I}e_{s}^{i}} & if\;\sum_{i\in I}e_{s}^{i}\geq 1\\ 0 & if\;\sum_{i\in I}e_{s}^{i}<1 \end{array}\right.\;\forall s\in S\;\mathrm{where}\;O_{s}^{i}=\max\left(U_{s}^{i}-T_{s},0\right)\leq O_{s}^{max}$$ (23)

In Eq (23) the expected over-usage is computed relative to those scenarios which exceeded the time availability. Hence, the denominator is not |*I*| the total number of scenarios. This equation has two parts, to guard for divisions by zero.

### 3.5. Final remarks

The proposed SHCAM-SAA is a very large mixed integer programming problem, primarily due to the number of scenarios that would be necessary to evaluate. Scenario reduction techniques, however, can be applied in some circumstances. In the literature, the predominant approach involves the generation of a reduced set of the most probable scenarios. Each scenario is given a probability of occurrence that is used to scale the results [55]. Further research, outside the scope of this article, however, is necessary to identify if that course of action is possible for our QAHC.

The expected values given by Eqs (22) and (23) may be restricted should the need arise, for instance, via the inclusion of some additional “simple” bounds, $U_{s}^{LB}\leq E[U_{s}]\leq U_{s}^{UB}$ and $O_{s}^{LB}\leq E[O_{s}]\leq O_{s}^{UB}$. The ratio of over usage to usage may also be indicative, and something worth measuring and restricting, for instance, via a constraint of the form $E[O_{s}]\leq {⋔}_{s}E[U_{s}]$ or $\sum_{i\in I}O_{s}^{i}\leq {⋔}_{s}\sum_{i\in I}U_{s}^{i}$ where ⋔_*s*_ is a target threshold. It is worth noting that the percentage utilization and slackness can be computed, just like it was for the deterministic case. Instead of using $E[U_{s}]=\sum_{\forall a\in A_{s}}\beta_{a,s}E[t_{a}]$ however, we can use Eq (22).

## 4. Solving the stochastic model

We investigated the solution of the SHCAM-SAA using the IBM ILOG CPLEX (V12.10) mixed integer programming solver. Although functional, the model could only be solved to optimality for a very small demonstrative example with a handful of activities and treatment spaces. Weak performance is accentuated by poor upper and lower bounds and an excessive number of binary variables proportional to the number of scenarios considered (i.e., |*S*||*I*|). The number of constraints required to compute the utilisation level in (13), across all scenarios and spaces, is immense. The number of constraints required is dictated by the upper bound ${\hat{\beta }}_{a,s}$ which is particularly large in most practical situations. We conclude that a typical real-life scenario with hundreds of treatment spaces, tens of patient types with dozens of pathways, resulting in hundreds of activities, would not be possible to handle in this way. As such, this avenue was discarded, and another approach was taken.

### 4.1. Overview of proposed solution approach

Chance constrained optimization problems are intractable [44]. The difficulties encountered in solving the MIP model of Section 4.3 may be bypassed by decomposing the problem into an upper and lower-level decision problem, as shown by Eqs (24)–(26), along the same lines as [8, 9, 37, 41].


$$\mathrm{Upper}\;\mathrm{Level}.\;\mathrm{Maximize}\;\omega_{1}N-\omega_{2}SLV\;\mathrm{Subject}\;\mathrm{to}:\;0\leq N\leq \hat{N}$$ (24)

***Lower Level*.** Minimize *SLV* Subject to: Eqs (2) and (4) and the following:

$$SLV=\sum_{\forall s\in S}\max\left(E[U_{s}]-T_{s},0\right)E[U_{s}]=\sum_{a\in A_{s}}\beta_{a,s}E[t_{a}]\forall s\in S\;[DET]$$ (25)

$$SLV=\sum_{\forall s\in S}\max\left({SL}_{s}-Pr(U_{s}\leq T_{s}),0\right)\;[STOCH]$$ (26)

This is essentially a Lagrangian relaxation of the original problem [43]. This description is equivalent to the original model but excludes all details relevant to calculating Pr(*U*_*s*_≤*T*_*s*_). In theory, this approach can make better use of parallel computing facilities and permit more scenario testing to be evaluated. Details about this will be presented in due course. Our two-level optimization approach is motivated by the presence of hierarchical decisions within the considered capacity identification problem and the dependence of one set of decisions on the other. For instance, $n_{g}^{1}$ and $n_{g,p}^{2}$ are dependent decision variables and can be computed directly. The calculations $n_{\gamma }^{1}=\mu_{\gamma }^{1}N$ and $n_{\gamma ,\psi }^{2}=\mu_{\gamma ,\psi }^{2}n_{\gamma }^{1}$ can be performed for any selected N and after any perturbation of N.

The upper level identifies the largest value of N, denoted $N^{opt}$, using a Binary Search Algorithm (BSA). It permits us to compute the number of patients of each type (i.e., the case mix), and the exact number of activities that need to be performed. BSA is a traditional iterative search algorithm for finding the position of a target value in a sorted array. At each step, one side of the array is eliminated. This depends upon a comparison of the target with the middle element of the array.

The allocation of activities to hospital spaces is a separate constraint satisfaction problem. For a particular value of N, the goal of the lower-level decision problem, is to identify which resources should be used to undertake the activities that would eventuate if the case mix (i.e., from the upper one) were realized. This is achieved using either a Threshold Acceptance (TA) or Simulated Annealing (SA) meta-heuristic. The purpose of the meta-heuristics is to identify whether a resource allocation with a sufficient level of reliability exists. In other words, does a solution with a service level violation (SLV) of zero exist. To find one, the resource assignments must be distributed so that the probability of resource over utilization is kept to an acceptable “specified” level. That level may in fact be zero, meaning no over utilization is permitted. Solving the allocation problem consumes all the computational time. The binary search is hence the repeated solution of the allocation problem.

It is worth pointing out that *SLV* = 0 for all values of $N\leq N^{opt}$. For all value $N>N^{opt}$ then *SLV*>0. It is also noteworthy to mention that there is no optimal allocation, as we do not differentiate between candidate hospital spaces. In theory we could, but that is outside the scope of this article. We could for instance rank spaces in one area more highly or less highly than spaces in another area. At present we assume that each space in *S*_*a*_ is equally valid.

As we are looking for the solution with the largest value of N with no SLV present, it is reasonable to select *ω*_1_ = 1 and *ω*_2_≫1. A large value of *ω*_2_ is needed to ensure that solutions with any *SLV* are heavily penalized. A reasonable value for $\omega_{2}=\hat{N}$. In the context of this application, further experimentation with different values of *ω*_1_, *ω*_2_ is not required and provide no additional benefit. It would be better to directly manipulate the parameter ${SL}_{s}$ if solutions with more/less inherent risk are deemed acceptable.

### 4.2. Implementation details

**Fig 1:**
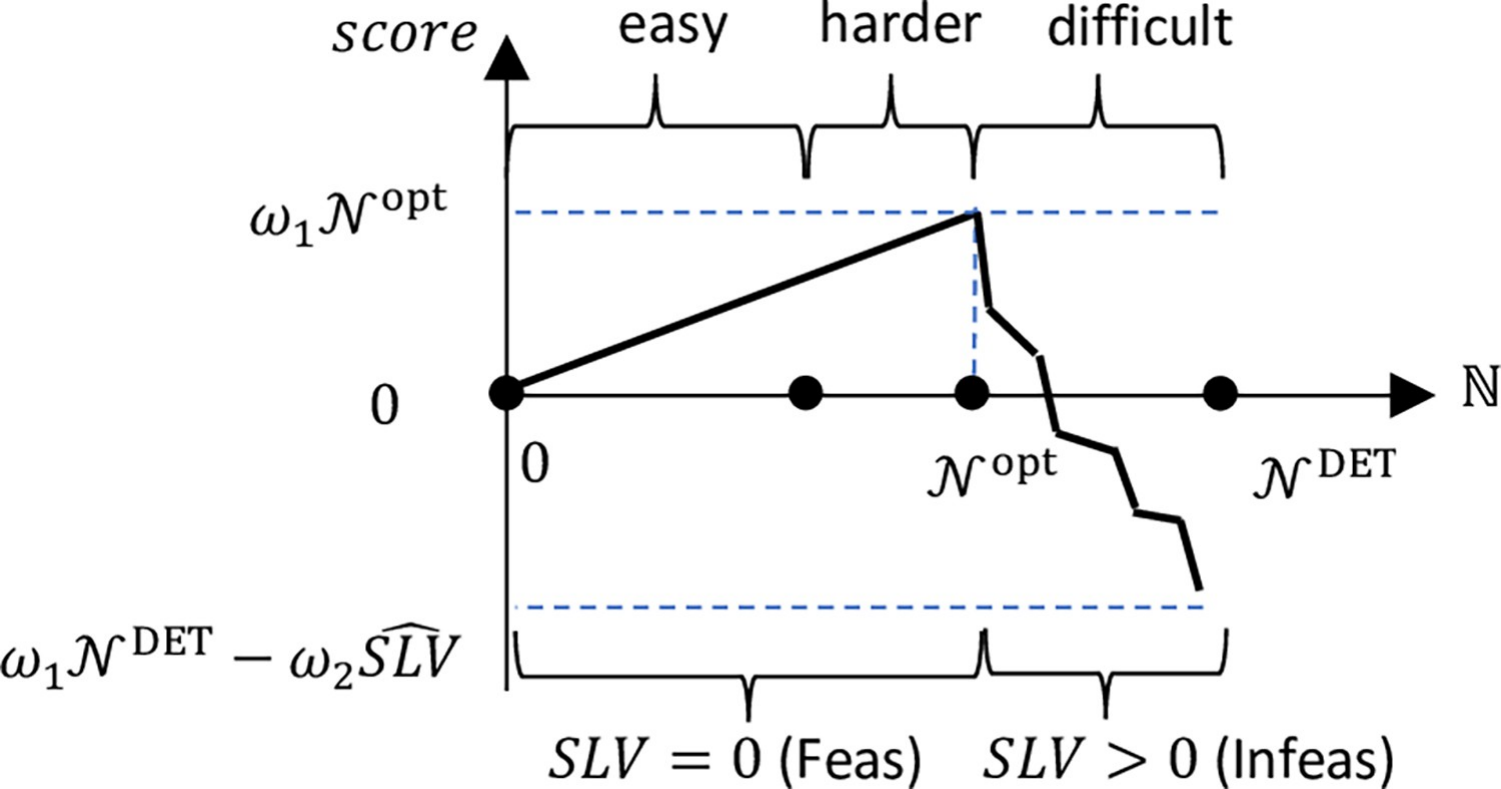
Summary of search space.

The exact details of our implementation can be found in Appendix C in S1 File. In our approach a capacity analysis for the deterministic case is first performed using Alg. 2. Then a capacity analysis for the stochastic case follows, using Alg. 3. The input to Alg. 3 is $N^{DET}$, the output of Alg. 2. Both approaches utilize binary search as described in Alg. 4. The binary search algorithm runs iteratively, and at each step, a new value of ℕ is chosen and a resource allocation with minimal *SLV* value is generated. The new value is $N\prime =N^{left}+\lambda (N^{right}-N^{left})$ where the scale parameter *λ*∈(0,1). At the end of each step, the left or right bound is updated appropriately. For instance, if $N^{\prime }\geq N^{left}$ then $N^{left}=N^{\prime }$, otherwise $N^{right}=N^{\prime }$. As shown in Fig 1, when N is relatively small, finding an *SLV* = 0 allocation is relatively easy and is not time consuming.

Beyond a certain size however, computing times increase, and a full search is required to prove or disprove the possibility. Due to this phenomenon, it is anticipated that the binary search will run fastest when its’ scale parameter is around 0.1 or 0.2. The reason for this hypothesis is that, with those values, the BSA can creep up towards the optimal without having to apply many full runs of the meta-heuristic. In contrast, if the scale is 0.7 or larger, the possibility of exceeding $N^{opt}$ is almost certain. *This hypothesis however will be tested in later numerical investigations*. The first part of the function shown in Fig 1 is linear, but the shape of the second part is problem specific, and either non-linear (concave, convex), piecewise linear, or stepped. Knowing the first part is linear, provides no help in locating the first N value where the SLV is no longer zero.

The proposed Binary Search Algorithm has been coded in a generic way to facilitate application to both deterministic and stochastic cases. The specified function references (a.k.a. handles), *makeAlloc*, *evalFn*, *evalSLV*, tell the BSA how to operate. For instance, how to generate an appropriate allocation, how to evaluate the caseload, how to evaluate the risk of over utilization, etc. The evaluation procedures are shown in Alg. 5–8. Algorithm 5 takes as input the number of patients, namely ℕ, and translates this into proper $n_{g}^{1}$ and $n_{g,p}^{2}$ values. Then an allocation is created by running the meta-heuristic. The number of SLV is computed and a score is returned. Algorithm 6. And Algorithm 7 compute the number of SLV for the deterministic and stochastic situations, respectively. Parallel processing can be used in Alg. 7 as each hospital space is independent. For the stochastic situation, Algorithm 8 is pivotal. It simulates the utilization of hospital spaces and provides a value for Pr(*U*_*s*_≤*T*_*s*_). The details behind Algorithm 8 are now discussed.

Realisations of the activity durations are required for evaluating the utilization of each treatment space. These values are determined via distribution sampling procedures and can be generated upfront or else computed “ad-lib”. It is necessary to use one set of scenarios, otherwise a solution can be scored differently each time. As our numerical testing indicates, generating large numbers of durations “ad-lib” is slow on a typical personal computer, so it is best to generate |*I*| complete scenarios upfront. Depending on the circumstances of a problem instance, the number of values can also be excessive. For the parameter $t_{a,s}^{i,n}$, the required number is $\sum_{i\in I}\sum_{a=(g,p,k)\in A}\sum_{s\in S_{a}}{\hat{n}}_{g,p}^{2}$. If alternative treatment spaces are identical, one way to cut down the values is to define and record durations $t_{a}^{i,n}$ instead, where $n=1,\ldots ,{\hat{n}}_{g,p}^{2}$. The number of elements to be stored is hence $\sum_{i\in I}\sum_{a=(g,p,k)\in A}{\hat{n}}_{g,p}^{2}$.

Provided that *β*_*a*,*s*_>0 it is necessary to choose the durations that will be realised for each of the ⌈*β*_*a*,*s*_⌉ occurrences of activity *a*. These will be extracted from the generated $t_{a}^{i,n}$ values. If every treatment space is re-evaluated every time a perturbation is made, then a simple “indexation” counter need only be defined for each activity and updated every time a value is extracted. Re-evaluating the utilization level of all treatment spaces is rather extreme, however, given that a perturbation (described later) affects only two treatment spaces. So, we only re-evaluate two treatment spaces.

The selection of sampled activity durations adds another layer of complexity to the implementation. It is necessary to explicitly record which sampled durations are used to permit a consistent evaluation that is invariant from one evaluation to the next. Let us define ℒ_*a*,*s*_ as a dynamic list of selected “lookup indices” for the allocation *β*_*a*,*s*_ such that |ℒ_*a*,*s*_| = ⌈*β*_*a*,*s*_⌉. Any value in the set $\left\{1..{\hat{n}}_{g,p}^{2}\right\}$ can be chosen, but once selected, a particular index cannot be reused. Hence, it is necessary to keep track of unused indexes too. Each index is a lookup for a particular sampled duration. Hence, ℒ_*a*,*s*_[*n*] is the lookup for the nth realisation. For example, if *β*_*a*,*s*_ = 2.73 and ℒ_*a*,*s*_ = (2,6,7), then the values $t_{a}^{i,2},t_{a}^{i,6}$ and $t_{a}^{i,7}$ are used to compute the utilization of treatment space *s*. The occupancy level for space *s* in scenario *i* is computed in the following way:

$$U_{s}^{i}\leftarrow \sum_{a\in A_{s}}\left[\sum_{n\in \{1..\lfloor \beta_{a,s}\rfloor \}}t_{a}^{i,L_{a,s}[n]}+(\beta_{a,s}-\lfloor \beta_{a,s}\rfloor )t_{a}^{i,L_{a,s}[\bar{n}]}\right]\;where\;\bar{n}=\lceil \beta_{a,s}\rceil$$ (27)

The following example (i.e., see Table 1) demonstrates how a different number of sampled values is needed for two different allocations, the second obtained after the first is perturbed. The second allocation requires ten values, one more than the first.

**Table 1:** Shifting 0.3 occurrences of an activity from *s* =1 to *s* =3.

Space	*s* = 1	*s* = 2	*s* = 3	→	Space	*s* = 1	*s* = 2	*s* = 3
*β*	3.42	2.21	1.9		*β*	3.12 (i.e., 3.42–0.3)	2.21	2.2 (i.e., 1.9 + 0.3)
ℒ	[1,2,3,4]	[5,6,7]	[8,9]		ℒ	[1,2,3,4]	[5,6,7]	[8,9,10]

### 4.3. Finding resource allocation solutions

A key aspect of the proposed solution process is allocating hospital activities to hospital spaces. The decision variables are *β*_*a*,*s*_ and these are considered as real-valued. In theory, an MIP solver could be used for both the deterministic and stochastic versions. However, that task is often intractable. As such, a polynomial time algorithm has been developed to quickly construct an appropriate allocation with a high chance of obtaining an *SLV* score of zero if it is feasible to do so. The exact details can be found in Algorithm 9 (i.e., the kernel) and Algorithm 10. Our approach assigns each activity in the caseload to a hospital treatment space. Algorithm 9 is iterative and handles each pathway in turn. An equally acceptable alternative is to create a list of activities (i.e., an “*activity ordering”*) and to iteratively consider those in turn.

In Algorithm 10, the activities within a pathway are assigned treatment spaces. The first step is to create a candidate list, and to rank options from largest to smallest free time. The first option is always selected (and then removed) and the maximum number of activities is assigned. The remaining number to assign is then revised and the utilization is updated. This process is repeated until no more activities need to be allocated. It is worth noting that when $N>N^{opt}$, it will be necessary to assign activities to spaces that have no free time.

The approach described above will in most situations find an allocation that results in a zero *SLV* if it is possible. There are cases, however, where an allocation with *SLV*>0 is created unnecessarily. For example, consider two activities *A* = {*a*_1_, *a*_2_} with treatment durations $t_{a_{1}}=60;\;t_{a_{2}}=45$ respectively. Also consider that there are two treatment spaces *S* = {*s*_1_, *s*_2_} with $T_{s_{1}}=T_{s_{2}}=480$, and *a*_1_ can be performed in both, yet *a*_2_ can only be performed in the second, i.e., $S_{a_{1}}=\{s_{1},s_{2}\}$; $S_{a_{2}}=\{s_{1}\}$. If $\left[n_{a_{1}},n_{a_{2}}]=[10,7\right]$ then there are two activity orderings:

Ordering 1: {*a*_1_, *a*_2_}. Result: $\beta_{a_{1},s_{1}}=8;\;\beta_{a_{1},s_{2}}=2;\;\beta_{a_{2},s_{1}}=7;\;U_{s_{1}}=795;U_{s_{2}}=120;\;SLV=315>0$

Ordering 2: {*a*_1_, *a*_2_}. Result: $\beta_{a_{2},s_{1}}=7$; $\beta_{a_{1},s_{1}}=2.75;\;\beta_{a_{1},s_{2}}=7.25$; $U_{s_{1}}=480;\;U_{s_{2}}=435;\;SLV=0$

If the first activity ordering is applied, the algorithm would produce an *SLV*>0 solution. However, if the second ordering is applied, a feasible *SLV* = 0 solution is produced. The first ordering fails because activity *a*_1_ is assigned to space *s*_1_ first, leaving no spare capacity for *a*_2_ which in this situation has no other candidate locations. The second ordering however is fine. The order in which activities are assigned is evidently important. The issue we have just described could be avoided in several ways (i.e., perhaps by ordering activities according to the number of candidate spaces available), but the simplest option would be to apply a meta-heuristic.

### 4.4. Meta-Heuristic approach

At each step of the binary search algorithm a resource allocation is obtained for the current value of N. The *SLV* value of that allocation is then compared to the current lower and upper bound. To find an *SLV* = 0 allocation as quickly as possible, we use a meta-heuristic (MH). Once an *SLV* = 0 allocation is found, the MH is terminated.

Meta-heuristic methods are fast, efficient, and relatively easy to implement. A variety of meta-heuristics are applicable but, in this article, we use SA and a variant, TA. Simulated Annealing is perhaps the most successful probabilistic technique for approximating the global optimum of a given function [56–60]. A key feature of SA is the cooling schedule that mimics the annealing process of metals. Another key feature is the capability to accept non improving moves to escape locally optimal solutions. In SA, a single solution to the problem is maintained. This solution is then repeatedly perturbed using a variety of strategies, including local improvement operators. Threshold Acceptance is a variant of SA. In TA, perturbed solutions are accepted if they are strictly better, or else not much worse than the original un-perturbed solution. A threshold parameter, initially set large and gradually reduced over time, quantifies the acceptable difference in quality.

Each meta-heuristic is applied through Alg. 11. Population and agent-based approaches are avoided, as there is a need to perform many time-consuming simulations during the evaluation process. Conceptually SA and TA are quite similar. In both algorithms, new solutions are “accepted” if they are strictly better than the current solution. In SA, however, solutions of inferior quality are accepted probabilistically, according to the current temperature and the extent of the “difference”. In early stages of the search, many inferior solutions are accepted, but at the end, none are. In TA, inferior solutions are accepted if they are not too much worse. This means that the new solution is accepted if the difference between the current solution and the new one is less than the current threshold. The details of the SA and TA meta-heuristic can be found in Alg. 12–16. The initialisations occur in Alg. 12, after which the “iterate” function (see Alg. 13) is repeatably applied until the termination criterion is met. The termination criteria *SLV* = 0 or *t*<*t*_*F*_ is evaluated. A limited number of perturbations are made within Alg. 13. The control parameters for SA and TA are then updated accordingly.

Various perturbation strategies are available. A greedy approach (see Alg. 14) is suggested as we assume hospital spaces are equivalent. As such, it is unnecessary to find an optimal allocation, just one that does not overload the hospital spaces. The purpose of Alg. 14 is to re-allocate activities from spaces that are highly utilized, to spaces less so. First, spaces are sorted according to their current probability of over utilization, as given by Pr(*U*_*s*_>*T*_*s*_). The worst space is selected and a randomly selected “assigned” activity is re-allocated. The amount of the re-allocation is chosen randomly. However, there is some evidence to suggest that it should be chosen proportional to the maximum over-utilization observed during simulations and the expected activity duration, i.e., $amt=\min\left(\frac{O_{s}^{max}}{E[t_{a}]},\beta_{a,s}\right)$. The re-allocation is accepted or rejected according to Alg. 15 or Alg. 16.

### 4.5. Additional complexities and extensions

To this point, it has been assumed that specified case and path mixes are strictly enforced. A relaxation of this assumption is worth considering and has previously been shown to result in a higher level of capacity [3]. To facilitate a relaxation, one option is to introduce tolerances $\Delta_{g}^{1}$ and $\Delta_{g,p}^{2}$ and the following constraints to the model:

$$\mu_{g}^{1}N-\Delta_{g}^{1}\leq n_{g}^{1}\leq \mu_{g}^{1}N+\Delta_{g}^{1}\;\forall g\in G$$ (28)

$$\mu_{g,p}^{2}n_{g}^{1}-\Delta_{g,p}^{2}\leq n_{g,p}^{2}\leq \mu_{g,p}^{2}n_{g}^{1}+\Delta_{g,p}^{2}\;\forall (g,p)\in GP$$ (29)

In this option, apart from small deviations, the specified mixes will be met. Another alternative is to introduce explicit ranges $\left[\mu_{g}^{1[LB]},\mu_{g}^{1[UB]}\right]$ and $\left[\mu_{g,p}^{2[LB]},\mu_{g,p}^{2[UB]}\right]$ and the following constraints:

$$\mu_{g}^{1[LB]}N\leq n_{g}^{1}\leq \mu_{g}^{1[UB]}N\;\forall g\in G$$ (30)

$$\mu_{g,p}^{2[LB]}n_{g}^{1}\leq n_{g,p}^{2}\leq \mu_{g,p}^{2[UB]}n_{g}^{1}\;\forall (g,p)\in GP$$ (31)

This option would permit greater variations to be considered.

In both extensions, the resulting case and path mixes become decision variables. To compute those variables, we need to evaluate the expressions $\mu_{g}^{1}=n_{g}^{1}/N$ and $\mu_{g,p}^{2}=n_{g,p}^{2}/n_{g}^{1}$. This, however, cannot be done within the model, as the right-hand sides become non-linear. Though it can be done after the model solves.

## 5. Numerical testing

In this section, we evaluate the effectiveness of the proposed solution approach. The focus of numerical testing is to identify the effect of different parameters on the solution process, and more generally to identify the best parameters to use to obtain the highest-quality solutions, in the least amount of time. Our numerical experiments have been run on an SGI Altix XE Cluster. During the numerical testing, we have recorded the number of steps required by the binary search, the run times, theoluteions obtained for both deterministic and stochastic situations. Each problem instance is solved ten times. Four processors are used for “each run” to mimic application on a typical desktop laptop, and 15 GB memory is assumed. The following parameters have been tested:

Binary Search: *scale* ∈ {0.1,0.2,…,0.9}

Service/ Reliability Levels: SL∈{0.8,0.95,0.9,0.975,0.95,0.99};

Risk Level (a.k.a., threshold): RL(=1−SL)∈{0.01,0.025,0.05,0.1,0.15,0.2}

Sample Averaging scenarios: {500,1000,2000}

Meta heuristic: *alg* ={*SA*, *TA*}, *t*_*I*_= *SLV*.*λ*, *t*_*F*_ = 0.001, $t_{R}=e^{\left[\ln(t_{F}/t_{I})/50\right]},$ *λ* = 1*E*6, *steps* = 300

This results in 3240 test instances. To show the full capability of the approach a large sized instance is solved for a period of 52 weeks. This choice is consistent with previous articles on the topic, that considered the same time frame.

### 5.1. Case study

In our case study, we consider a large public hospital approaching 1000 treatment spaces. These reside in 65 areas, predominantly wards. There are also surgical care areas for preoperative (preop) and post anaesthesia care (pac), and 19 operating theatres for surgeries. There are 21 surgical specialties and their respective units. For each of these we consider several patient types. The first is a “typical” elective patient with a day of surgery arrival (DOSA) and no ICU stay. The pathway is hence (preop, sur, pac, postop). The second is also a DOSA, but in contrast has an ICU stay directly after surgery; hence the path is (preop, sur, ic, postop). The other two types are acute patients. They have the same pathways as just described. We assume that the pre-operative care activity occurs in a surgical care unit and not in a ward. In the considered pathways, the durations for each of the activities are random variables. The random variables have been constructed using data collected from the hospital in our case study.

For that analysis, the data has been aggregated by specialty and by the following activity types (ic, pac, postop, preop, sur). For our analysis, each activity duration is modelled by a *piecewise constant distribution* that produces floating point values uniformly distributed over each contiguous sub-interval. As such we have extracted a list of breakpoints and weights. Adjacent breakpoints define the sub-intervals, and the weights describe the probability of a value occurring within a sub-interval.

Predominantly, the activities have characteristics of normal, log-normal, exponential, beta distributions. The extracted distributions, however, are quite varied and have significantly large tails. The full details of the random variables are large and cannot be summarised easily. Consequently, they have been listed in our accompanying data and results document. Other important details concerning the number of spaces in each area can also be found in that document. The considered patient type case mix and sub type mix for the analysis are shown in Table 2. As such the first column sums to one, and the bracketed values also sum to one. The number of terms in each bracket is the number of sub types.

**Table 2:** Specified case and path mix.

#	Patient Type	Patient Case Mix	Patient Sub Type Mix
1	Acute Surgical	0.036	(0.95, 0.05)
2	Breast and Endocrine	0.033	(0.8455, 0.0445, 0.1045, 0.0055)
3	Colorectal	0.027	(0.6365, 0.0335, 0.3135, 0.0165)
4	Cardia Surgery	0.056	(0.7695, 0.0405, 0.1805, 0.0095)
5	Dental	0.0	(1.0)
6	Ear, Nose & Throat	0.036	(0.7695, 0.0405, 0.1805, 0.0095)
7	Faciomaxillary	0.028	(0.513, 0.027, 0.437, 0.023)
8	Gastroenterology	0.012	(0.95, 0.05)
9	Gynaecology	0.0	(1.0)
10	Hepatobiliary	0.027	(0.703, 0.037, 0.247, 0.013)
11	Liver Transplant	0.00489	(0.1616, 0.0085, 0.7885, 0.0415)
12	Neurosurgery	0.043	(0.6745, 0.0355, 0.2755, 0.0145)
13	Ophthalmology	0.163	(0.9025,0.0975)
14	Orthopaedic	0.216	(0.4085, 0.0215, 0.5415, 0.0285)
15	Plastics	0.109	(0.7695, 0.0405, 0.1805, 0.0095)
16	Respiratory	0.0	(0.95, 0.05)
17	Renal Transplant	0.041	(0.608, 0.032, 0.342, 0.018)
18	Trauma	0.003	(0.95, 0.05)
19	Upper Gastro-Intestinal	0.03811	(0.836, 0.044, 0.114, 0.006)
20	Urology	0.086	(0.8265, 0.0435, 0.1235, 0.0065)
21	Vascular	0.041	(0.532, 0.028, 0.418, 0.022)

### 5.2. Main results

For the deterministic case, our approach identified the capacity as 12444.4 treatments per year in under a second of computing time. The obtained caseload shown below, matches the proportions specified in Table 2.

[448, 410.667, 336, 696.889, 0, 448, 348.444, 149.333, 0, 336, 60.853, 535.111, 2028.444, 2688,1356.444, 0,510.222, 37.333, 474.258, 1070.222, 510.222]

The number of steps required by the binary search is dependent upon the scale parameter used. We considered nine options, and as such, applied the BSA nine times. The number of steps were (51,32,23,28,25,25,31,25,56) respectively. Evidently, the fewest are needed when the scale is around 0.5.

**Fig 2:**
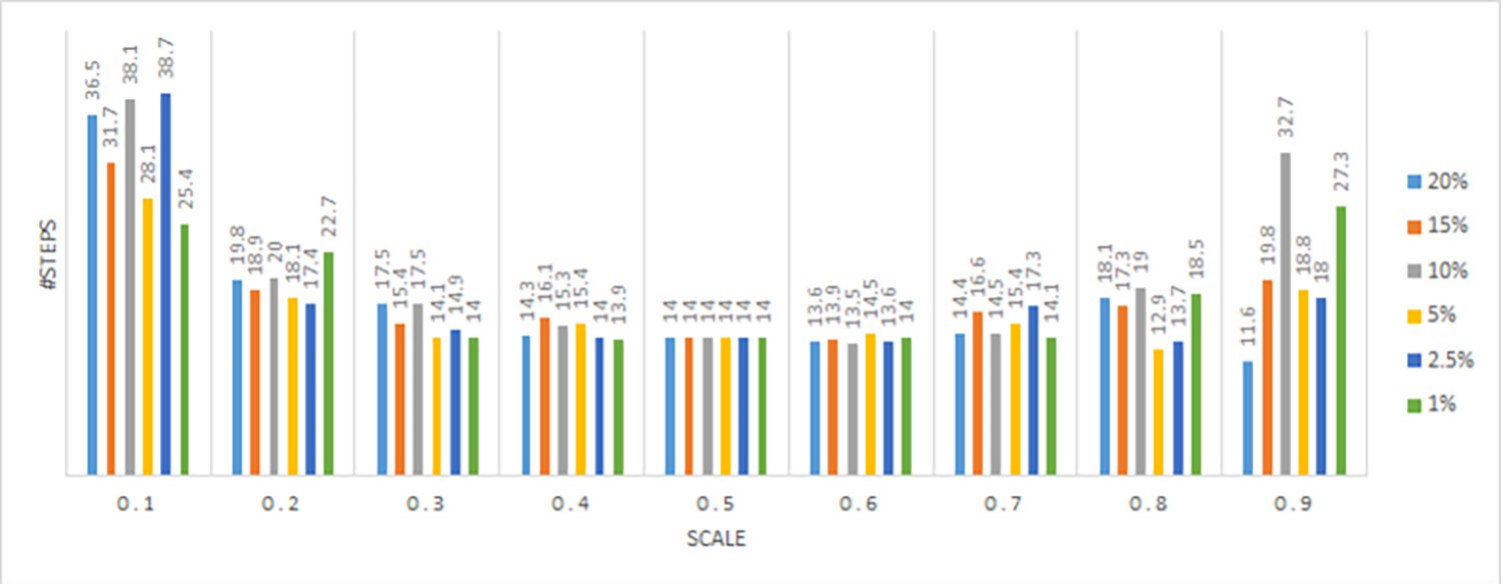
Average number of search steps required for different thresholds of risk.

**Fig 3:**
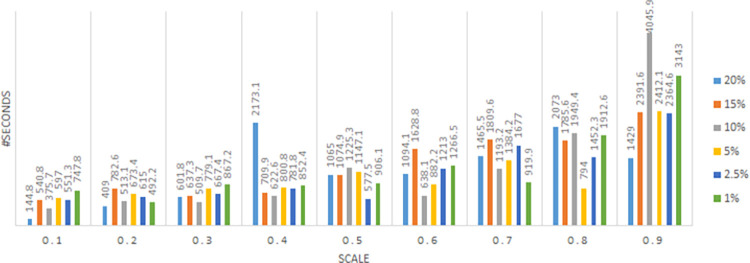
Average run time required for different thresholds of risk.

**Fig 4:**
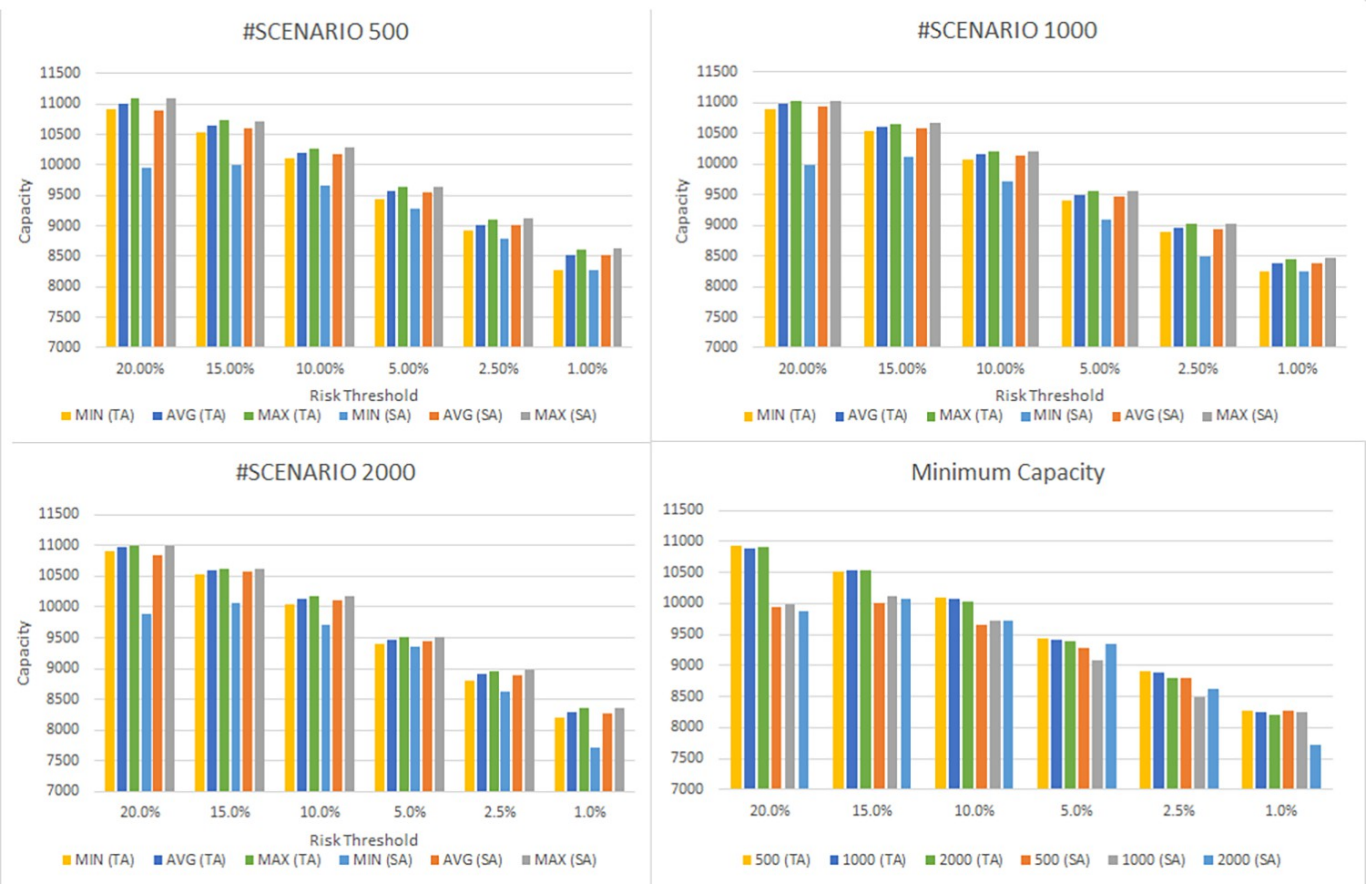
Capacity level for different thresholds of risk and scenario numbers.

The results obtained for the stochastic case are now discussed. The full set of “aggregated” results can be found in the supplementary data and results document. Overall, the number of scenarios did not seem to affect the number of steps required by the binary search. The chart in Fig 2 is representative and summarises the results of TA for 2000 scenarios. The fewest steps occurred when the scale parameter is 0.5 or 0.6. For small and large values of the scale parameter, more steps are required. The run times for different scale values are shown in Fig 3. For the same scale parameter, it is easy to see that the run time is clearly higher when the number of scenarios is increased. It is worth noting that more steps are not actually bad; the solve time is smallest when the scale parameter is 0.1. Run times however are worst when the scale parameter is 0.9. The reason for this is quite clear. When the scale parameter is smaller than 0.5, the solution is more likely to be feasible, hence the meta-heuristic can easily find an allocation that balances the variability of the treatment durations. The meta-heuristic then terminates early. When the scale parameter is larger, the solution is often infeasible, and a full solve is required to prove infeasibility. In other words, no shortcut occurs. The solution quality, number of steps and run time varies considerably from one replication to another, and for different scales and scenario numbers. The results in Figs 2 and 3 are for the average case. To see the full effect of the variation, Fig 4 has been provided. Even for a particular number of scenarios, there is a big difference in the capacity. The minimum capacity achieved for different scenario numbers and risk thresholds is shown in the fourth chart. We can see from Fig 4 that as the number of scenarios is increased, the capacity decreases. This is because more adverse realisations of the activity durations are included. Evidently the reduction in capacity could be quite significant. There does not seem to be any major differences between the TA and SA algorithm. On some instances one is better than the other in terms of either solution quality or running time. However, upon closer scrutiny SA is not as good at finding the best allocation and returns “non-optimal” allocations on occasion. This causes the binary search to output a reduced capacity. This reduced capacity can be seen by inspecting the minimum capacity values (i.e., in Fig 4) and not the averages and maximums.

### 5.3. Additional scenario testing

A greater number of scenarios was considered next in our numerical testing to further understand the difference in solution quality. Theoretically, sample averaging should become more accurate with an increased number of scenarios, and we would expect to see convergence of the capacity to some value. Scenarios numbers of 5000, 10000, 15000 and 20000 were applied next. We used the value 0.1 for the BSA scale and solved each instance 10 times. At 20000 scenarios, 16GB of memory is used. To test higher numbers on a laptop or other desktop PC, more memory is required, or else the scenarios should be stored on the hard drive, or generated “incrementally” when required, and not stored in entirety. Another option is to assess a shorter time frame, rather than a year. This would then allow a greater number of scenarios to be generated, as fewer realisations of the different care plans would occur.

**Fig 5:**
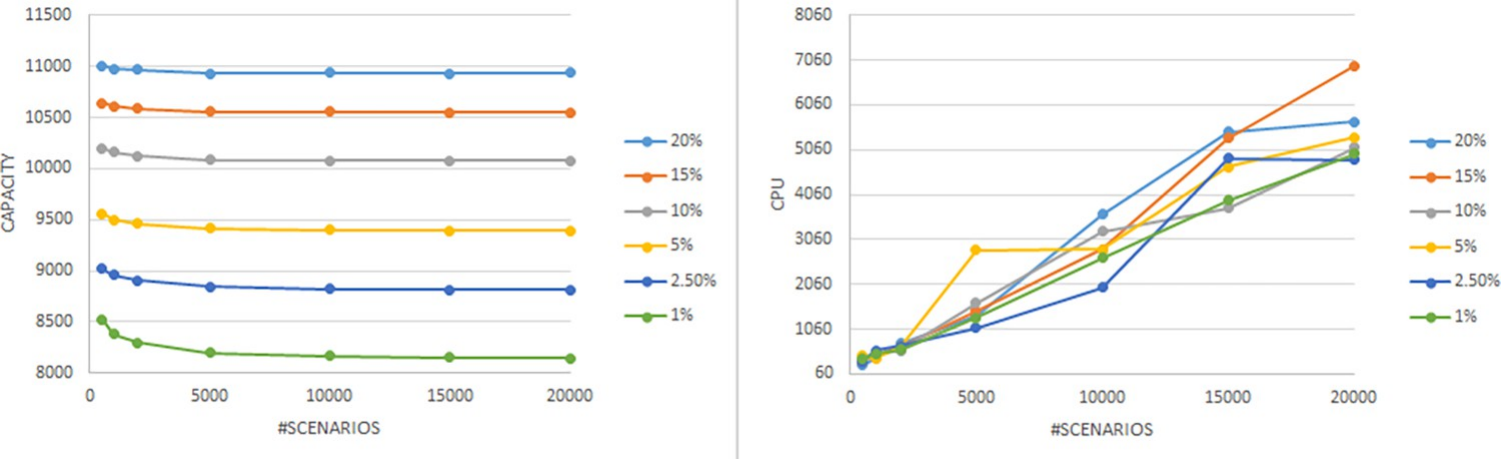
Capacity level and CPU for different risks and scenario numbers.

**Fig 6:**
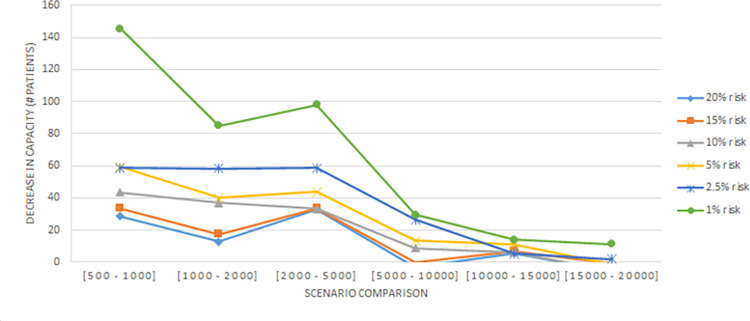
Observed decreases in capacity.

In Fig 5, the new results are summarised. In Fig 5A, the capacity level for different scenarios numbers and thresholds of risk are shown. The capacity continues to decrease with increased scenario numbers, and there is evidence of convergence at around 20000, regardless of the risk threshold used. More precise details of the decreases are shown in Fig 6. In Fig 5B the solve time increases linearly with the number of scenarios.

The fluctuations that occur in the observed “capacity decreases” shown in Fig 6 arise because only 10 repetitions have been considered. As such, the average reduction can be variable. For instance, in Fig 6, the reduction in capacity when increasing from “5000 to 10000” scenarios did not decrease as much as it did for the “2000 to 5000” case.

During numerical testing different caseloads were identified–those shown in Table 3 are indicative of “the best” caseload solutions that can be achieved for different levels of risk.

**Table 3:** Caseload identified for different risk levels (20000 scenarios).

	Deterministic Case	Risk = 20%	15%	10%	5%	2.50%	1%
AS	448	394.08	380.11	363.38	338.86	317.93	293.87
BE	410.67	361.24	348.43	333.1	310.62	291.44	269.39
COLO	336	295.56	285.08	272.53	254.15	238.45	220.41
CARD	696.89	613.01	591.28	565.26	527.12	494.56	457.14
ENT	448	394.08	380.11	363.38	338.86	317.93	293.87
FMAX	348.44	306.5	295.64	282.63	263.56	247.28	228.57
GAST	149.33	131.36	126.7	121.13	112.95	105.98	97.96
HPB	336	295.56	285.08	272.53	254.15	238.45	220.41
LTPT	60.853	53.53	51.63	49.36	46.03	43.19	39.92
NSUR	535.11	470.7	454.02	434.04	404.75	379.75	351.02
OPHT	2028.44	1784.29	1721.04	1645.3	1534.28	1439.53	1330.6
ORTH	2688	2364.46	2280.64	2180.27	2033.16	1907.6	1763.25
PLAS	1356.44	1193.18	1150.88	1100.23	1025.99	962.63	889.79
RTPT	510.22	448.81	432.9	413.85	385.92	362.09	334.69
TRMA	37.33	32.84	31.68	30.28	28.24	26.49	24.49
UGI	474.26	417.17	402.39	384.68	358.72	336.57	311.1
UROL	1070.22	941.41	908.03	868.07	809.5	759.51	702.03
VASC	510.22	448.81	432.9	413.85	385.92	362.09	334.69
	12444.44	10946.6	10558.5	10093.9	9412.77	8831.46	8163.18

**Fig 7:**
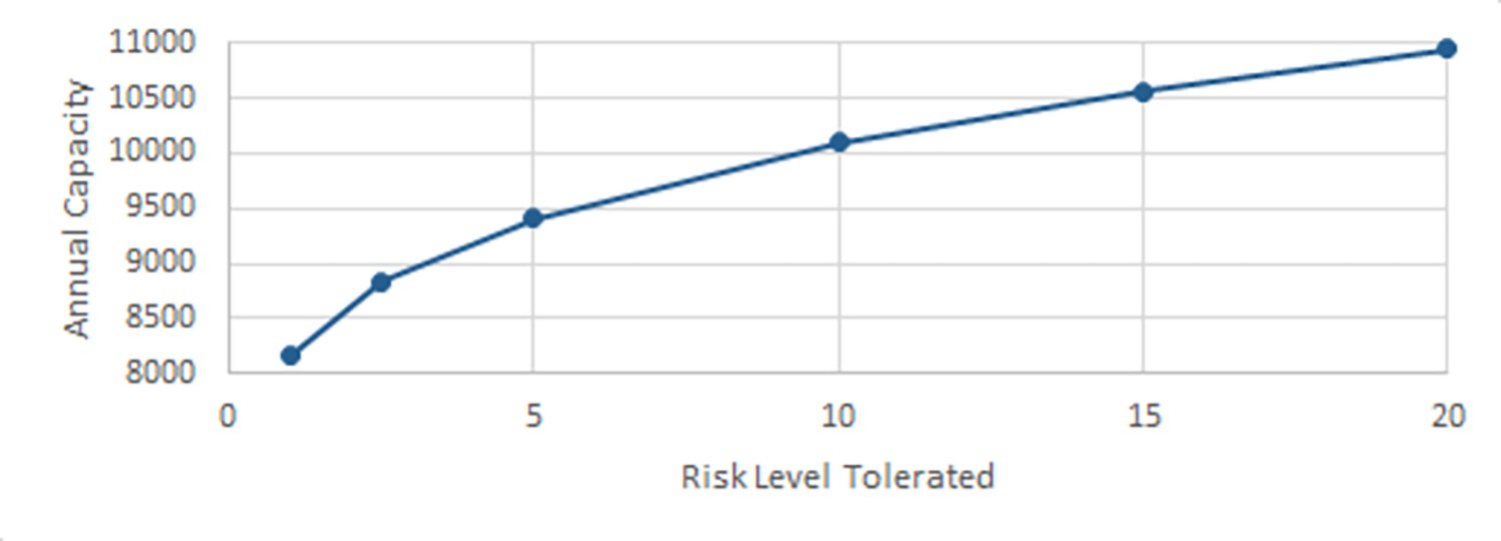
Trend in capacity (52 weeks).

Regarding hospital capacity and the risk threshold, it is apparent that the hospital capacity per annum is quite variable. Between the lowest and highest outputs, the range is 4281 patients per year, i.e., 357 per month or 89 per week. Fig 7 shows the general trend. There is a concave relationship, and as the tolerance for risk increases, the capacity tapers off. This implies that accepting higher risk has diminishing returns. Ideally, low risk and high output is most desirable, but that is not possible. If no possibility of exceeding the time availability of resources is permitted, then significantly less patients can be treated, however, that may ultimately result in far better care to those patients. Based upon multi-objective theories, the “sweet” spot lies between 10% and 15%, because those solutions are closest to the ideal. For the six thresholds, the normalized distances (i.e., between the solution and the ideal) are respectively as follows (0.344, 0.291, 0.249, 0.214, 0.213, 0.233).

### 5.4. Testing of reduced time frame

**Fig 8:**
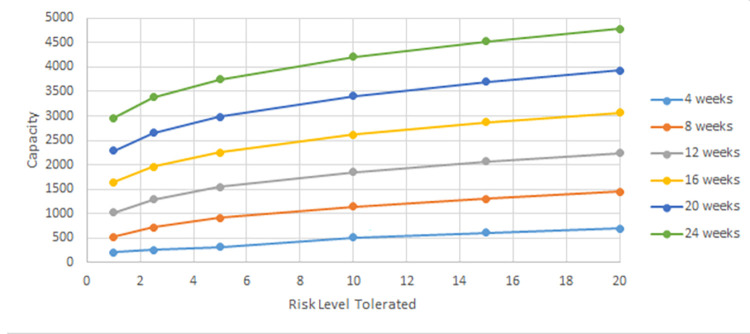
Capacity level for different levels of risk and for different time frames.

Till this point we have assessed hospital capacity for a 52-week period. A reduced time frame was considered next in our numerical testing to further understand the difference in solution quality, and to identify whether results for larger (or smaller) time frames can be simply scaled proportionally. In Fig 8 the results for 4, 8, 12, 16, 20 and 24 weeks is provided. To get those results we used a scale of 0.1 and 20000 scenarios.

**Fig 9:**
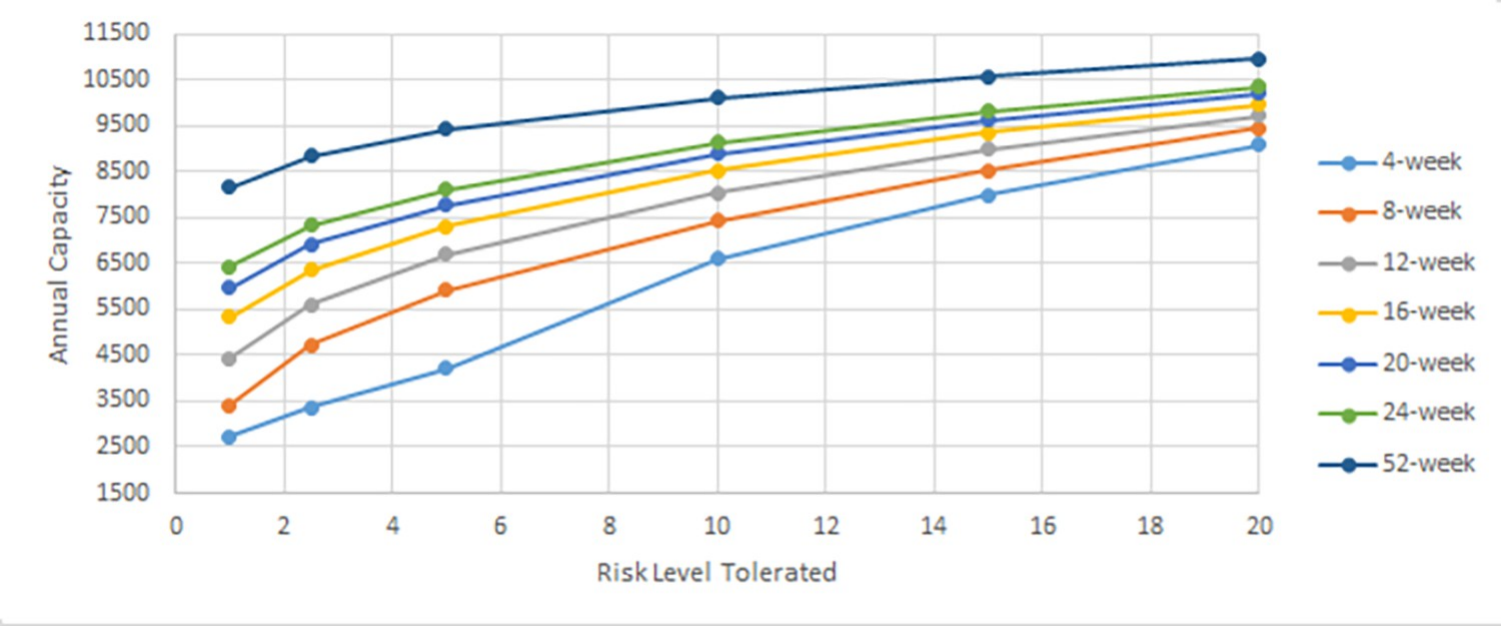
Annualised capacity for different levels of risk and for different time frames.

Fig 8 shows similar trends as the 52-week time frame in Fig 7. Upon closer scrutiny, its apparent that the results for the shorter time frames are not directly proportional to the 52-week results. To see this more clearly, the results in Fig 8 were scaled to provide annualised capacity. These are shown in Fig 9. Fig 9 shows a significant difference in capacity when aversion to risk is high. When aversion to risk is lower (i.e., at 20%), then differences are still present, but much less.

**Fig 10:**
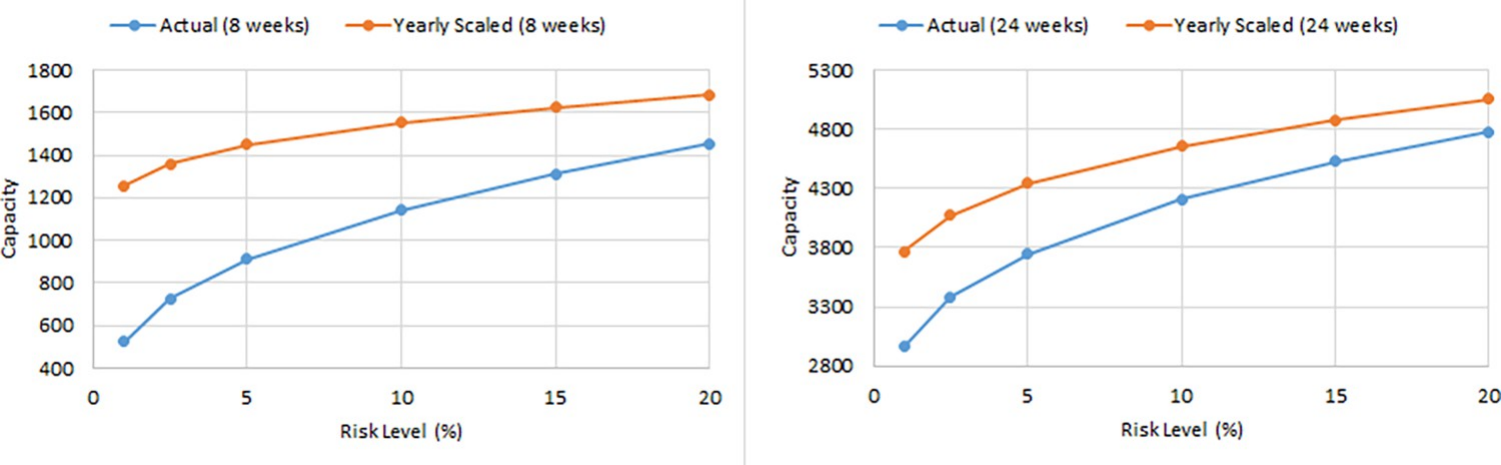
Comparison of results relative to the 52-week results.

Individual charts can be plotted for each analysis period. In Fig 10, two of the charts are shown for demonstrative purposes.

### 5.5. Final remarks

In conclusion it is not sufficient to scale up or down the results from different time frames when treatment durations and lengths of stay are stochastic. Theoretically, the maximum deterministic capacity (i.e., within any prescribed tolerance) could be reached for any given risk threshold, greater than zero, by choosing a sufficiently long time-horizon. This suggests that the time horizon should be carefully chosen based on managerial considerations. In practice, hospitals have operational flexibility to absorb short-term variability in resource utilisation and patient lengths of stay. For example, elective surgeries can be rescheduled, or some patients can be placed in alternative wards. However, these actions are not sustainable over an extended period and can lead to undesirable patient outcomes. It is therefore a process of consultation with hospital managers to inform the choice of time horizon and risk threshold, based on their experience and ability of the hospital to compensate in the short/medium term for variability in resource utilisation and patient lengths of stay.

## 6. Conclusions

### 6.1. Outcomes

For most patients treated in a hospital the surgery duration and length of stay is rarely static. Planning resource usage is therefore a significant challenge for hospitals. A hospital’s capacity and output are therefore tied to how much resource over-usage is permitted and/or tolerated. In this article the effect of stochastic treatment durations and lengths of stay on a hospitals’ capacity is investigated in a mathematically rigorous way for the first time. To identify that effect, a stochastic hospital capacity allocation model (SHCAM) is first proposed, as the basis of a comprehensive stochastic hospital capacity assessment and allocation (SHCA) approach. The purpose of the SHCAM is to determine the maximum number of patients that can be treated of different types, within a specified period, subject to case mix and time availability constraints, and sensitivity to the potential risk of resource over-usage. A plan describing which resources need to be assigned to each activity within each patient’s care pathway is also provided by the SHCAM.

The SHCAM has chance constraints, and those are handled using sample average approximation techniques (SAA). The resulting model (i.e., named SHCAM-SAA) is a large and intractable MIP formulation. To overcome this intractability, a novel “two-stage optimization” approach is proposed, which involves the application of a Binary Search Algorithm with an embedded meta-heuristic optimizer.

The number of scenarios evaluated in SAA is a critical consideration. Greater numbers should be evaluated for the most accurate assessment. A key consideration is the time-period over which the chance constraints are applied, with shorter time periods requiring fewer samples but also leading to more volatility between scenarios. The nature of the distribution of activity times also has an impact, with long tails also leading to more volatility and more scenarios required for convergence.

The scale and number of scenarios parameter are pivotal to the success of the SHCA approach; however, the effectiveness is also greatly affected, perhaps even more so, by the quality of the meta-heuristic strategy used and the specific parameters used to run them. The role of the meta-heuristic is to quickly find solutions with a service level violation (SLV) of zero if it is possible to do so. Our numerical testing indicates that TA and SA algorithms are adequate for this purpose, with TA slightly superior.

### 6.2. Managerial Insights and application

Earlier in the article we posed the question, “can an adequate approach be devised and put into practice”. To answer that question, it is necessary to discuss the pros and cons of the proposed approach, and to discuss the need if any to perform a QAHC. To the best of our knowledge a QAHC is not a task most hospitals currently perform, and as such there is no immediate need to motivate the implementation of an approach or a budget to put an approach into practice and to purchase a commercial license for state-of-the-art mathematical optimization software (i.e., like CPLEX or Gurobi), to facilitate one. A health care provider whose responsibility is to manage the public health system of a region or state, would be in a better position to value such an approach.

It would be fair to say that the details behind the proposed QAHC are somewhat intricate and nuanced. An in-depth knowledge is necessary to fully understand all the repercussions, variations, parameters, and possibilities that have been suggested. This is a limitation that may, at least initially, restrict application to real world problems by non-experts, like hospital managers and executives. A commercial grade implementation with a well-designed and simple to use graphical user interface would be a potential solution, worthy of further investigation and development. Beneficially, our approach requires no commercial solver and can be used as a “black-box”, requiring little intervention by hospital staff and other health care professionals once installed.

A major feature of the proposed approach are the thresholds of risk assigned to each hospital resource. Although this is a subjective value, it would be fair to say that low risk is particularly desirable in a health care setting and would be the first choice to evaluate at the start of a QAHC. Although higher outputs are achievable, high tolerances to risk have diminishing returns and have health care cost and quality repercussions. If higher outputs are required, it would be more sensible to use the results of the QAHC to inform hospitals how to reconfigure and expand their facilities and practices.

### 6.3. Future research

There are several directions future research could take. Alternative stochastic programming approaches and philosophies could be further investigated to see if they are competitive, like robust optimization, possibilistic programming, fuzzy logic, and joint chance constraints. Scenario reduction techniques should be further investigated as well to see if comparable solutions are obtained with less computational effort. It may also be useful to explore improvements to BSA, potentially applying an adaptive scale parameter. Other search methods such as Golden Section or Fibonacci search could also be explored. Both meta-heuristics were shown to be effective but must be run with an appropriate number of iterations and an appropriate perturbation strategy to ensure success. Otherwise, they may be prone to terminating with a service level violation when there does in fact exist a solution. These algorithms can be further improved to improve the overall solution scheme or else replaced with a MIP based solution approach. A caveat of the MIP solution approach, however, is tractability, as the number of scenarios is increased, and the annual cost of an MIP solver licence for hospitals.

It is anticipated that the extensions discussed in Section 4.5 concerning the alteration of the case mix would be quite worthwhile. A revised solution approach should be implemented and tested to identify the exact benefit achievable from relaxing case mix constraints. Should the two extensions discussed in Section 4.5 be introduced, the solution approach proposed in this article would need to change, as that would introduce new decision variables relating to independent throughputs of patient type and sub types. Additional metaheuristic perturbation operators could be devised to operate on these new decision variables; however, this comes with some challenges since perturbations made between the levels of patient type and sub-type would need to maintain consistency. It is, however, worth pointing out that the current perturbation operators for the allocation would remain unchanged in a new approach.

## Supporting information

S1 File (DOCX)

S1 Data (DOCX)

## List of abbreviations

BSA: Binary Search Algorithm
CC: Chance Constraints
CCP: Chance Constrained Problem
HCAM: Hospital Capacity Allocation Model
HCAP: Hospital Capacity Allocation Problem
JCC: Joint Chance Constraint
LOS: Length of stay
MIP: Mixed Integer Programming
QAHC: Quantitative Assessment of Hospital Capacity
SA: Simulated Annealing (meta-heuristic)
SAA: Sample Average Approximation
SHCA: Stochastic Hospital Capacity Assessment
SHCAM: SHCA Model
SLV: Service Level Violation
TA: Threshold Acceptance (meta-heuristic)
