# Supplementary material for: A stochastic programming approach to perform hospital capacity assessments
Source: PLoS One. 2023 Nov 9;18(11):e0287980. doi: 10.1371/journal.pone.0287980 (PMC10635551; doi:10.1371/journal.pone.0287980)
Supplement: S1 File — (DOCX) [file pone.0287980.s001.docx]

# Appendix A. Main Notation

Sets and parameters

$I$: Set of scenarios. Total number is $\left| I \right|$

$G$: Set of patient types (a.k.a. groupings)

$P_{g}$: Set of pathways for patients of type $g\in G$ (i.e., the patient sub type)

$A$: Set of patient care activities, occurring within patient care pathway

$A_{s},A_{w},A_{g,p}$: Set of hospital activities permitted in space $s$, ward $w$ and pathway $(g,p)$

$K_{g,p}$: Number of activities in the care pathway for patient type $g$, sub type $p$

$\Phi$: Set of treatment types (a.k.a. categories)

$W,W_{a},W_{\phi}$: Set of hospital areas, and those permitted for activity $a\in A$ and treatment type $\phi\in\Phi$

$S,S_{a},S_{\phi}$: Set of hospital treatment spaces, and those relevant to activity $a$ and treatment type $\phi$

$T_{s},T_{w}$: Time availability of treatment space $s\in S$ and treatment area $w\in W$

$t_{a}$: Random variable for the treatment duration of activity $a=(g,p,k)$

$t_{a,s}^{i,n}$: Treatment duration realisation (i.e., $n$th) for activity $a$ in scenario $i$ when performed at space $s$

$u_{a},\phi_{a}$: Unit performing activity $a=(g,p,k)$ and the activity type $\phi\in\Phi$

$\mu_{g}^{1},\mu_{g,p}^{2}$ : Proportional patient case mix and path “sub” mix

$\mathcal{SL}_{s}$: The service (a.k.a. safety) level defined for treatment space $s$

$U_{s},U_{s}^{i}$: Time that space $s$ is to be used, and in scenario $i\in I$

$U_{s}^{+}$: Maximum time that is available for space $s$

$O_{s}^{i}$: The amount of time in scenario $i\in I$ that space *s* is over-used, where $O_{s}^{i}=\max\left( U_{s}^{i}-T_{s},0 \right)$

$O_{s}^{\max}$: The maximum over-usage permitted, i.e., $\max_{i\in I} (O_{s}^{i})\leq O_{s}^{\max}$

$E\left[ O_{s} \right]$: Expected over-usage

Functions

$F_{t_{a}},F_{t_{a}}^{-1}$: CDF and inverse CDF for the random variable $t_{a}$

$\mathfrak{F}\left( n_{a},t_{a} \right)$: Function evaluates the time to process $n_{a}$ activities with duration $t_{a}$

Decisions

$\beta_{a,s},\hat{\beta}_{a,s}$: Number of activities of type $a$ assigned to treatment space $s$ and upper bound

$\alpha_{a,w}$: Number of activities of type $a$ assigned to treatment area $w$

$e_{s}^{i}$: Binary indicator of exceeded utilization, for hospital space $s$ in scenario $i$

$n_{g}^{1}(\hat{n}_{g}^{1})$: Number of patients of type $g$ (and the upper bound)

$n_{g,p}^{2}(\hat{n}_{g,p}^{2})$: Number of patients with pathway $p$ (and the upper bound)

$n_{a}^{3}$: Number of times activity $a$ occurs (i.e., the number of patients with this activity)

$\mathcal{N}$: Total number of patients treated

$\mathcal{L}_{a,s}$: The sampled durations (i.e., list) assigned to the allocation $\beta_{a,s}$

$B_{s}$: Buffering level for treatment space $s$

# Appendix B. Bounds

**Property 1.** $\hat{\beta}_{a,s}=T_{s}/t_{a} \forall a\in A_{s}$. **Proof.** If space $s$ is only used to perform activities of type $a$ then $\beta_{a,s}t_{a}\leq T_{s}$. Hence, $\beta_{a,s}\leq T_{s}/t_{a}$ and the upper bound is $T_{s}/t_{a}$.

**Corollary:** For a stochastic situation, $\hat{\beta}_{a,s}=T_{s}/t_{a}^{\min}$.

**Property 2.** $\hat{n}_{g,p}^{2}=\min_{\forall k\in\left\{ 1..K_{g,p} \right\}} \sum_{s\in S_{a}} \hat{\beta}_{g,p,k,s}$. **Proof.** The number of patients treatable is restricted by the number of activities within the plan that can be performed. By definition, $n_{g,p}^{2}=\sum_{s\in S_{g,p,k}} \beta_{g,p,k,s}\forall k\in\left\{ 1..K_{g,p} \right\}$ and $n_{g,p}^{2}\leq\hat{n}_{g,p}^{2}$. Clearly,$\beta_{g,p,k,s}\leq\hat{\beta}_{g,p,k,s}$ and $\sum_{s\in S_{g,p,k}} \beta_{g,p,k,s}\leq\sum_{s\in S_{g,p,k}} \hat{\beta}_{g,p,k,s}$. Therefore, $n_{g,p}^{2}\leq\sum_{s\in S_{g,p,k}} \hat{\beta}_{g,p,k,s}\forall k\in\left\{ 1..K_{g,p} \right\}$. Using bottleneck logic, $\hat{n}_{g,p}^{2}\leq\sum_{s\in S_{g,p,k}} \hat{\beta}_{g,p,k,s}$ and $\hat{n}_{g,p}^{2}=\min_{\forall k\in\left\{ 1..K_{g,p} \right\}} \sum_{s\in S_{g,p,k}} \hat{\beta}_{g,p,k,s}$.

**Property 3.** $\hat{n}_{g}^{1}=\frac{\hat{n}_{g,p}^{2}}{\mu_{g,p}^{1}}$. **Proof.** By definition, $n_{g,p}^{2}=\mu_{g,p}^{2}n_{g}^{1}$ and $n_{g,p}^{2}\leq\hat{n}_{g,p}^{2}$. Therefore, $\mu_{g,p}^{2}n_{g}^{1}\leq\hat{n}_{g,p}^{2}\Rightarrow n_{g}^{1}\leq\frac{\hat{n}_{g,p}^{2}}{\mu_{g,p}^{2}}$. By bottleneck logic,$n_{g}^{1}=\min_{p\in P_{g}} \left( \frac{\hat{n}_{g,p}^{2}}{\mu_{g,p}^{2}} \right)$.

**Property 4.** $\mathcal{N}\leq\hat{\mathcal{N}}=\min_{g\in G} \left( \frac{\hat{n}_{g}^{1}}{\mu_{g}^{1}} \right)$. **Proof.** $n_{g}^{1}=\mu_{g}^{1}\mathcal{N}$ and $n_{g}^{1}\leq\hat{n}_{g}^{1}$. Therefore, $\mu_{g}^{1}\mathcal{N}\leq\hat{n}_{g}^{1}\Rightarrow\mathcal{N}\leq\frac{\hat{n}_{g}^{1}}{\mu_{g}^{1}}\forall g\in G$. By bottleneck logic, $\mathcal{N}=\min_{g\in G} \left( \frac{\hat{n}_{g}^{1}}{\mu_{g}^{1}} \right)=\hat{\mathcal{N}}$.

# Appendix C. Algorithms

**Foreword**: Alg. 1 – 4 constitute the main “overriding” approach. Alg. 5 – 8 are solution evaluation procedures and Alg. 9 – 11 concern the creation of a resource allocation. Alg. 12 – 16 describe the details of the meta-heuristic used for creating a resource allocation. The terms, $evalSLV$, $makeAlloc$ and $evalFn$ are function pointers. Step 1-3 in Alg. 2 and 3 are not function calls, rather initialisations of the function pointers.

**Alg 1.** $\mathrm{QAHC}()$ // Main Capacity Allocation Procedure

1a. $\left| n^{1} \right|\leftarrow\left| n^{2} \right|\leftarrow\left| G \right|;$ $\left| n_{g}^{2} \right|\leftarrow\left| P_{g} \right|;$ $|\beta|\leftarrow|A|$; $\left| \beta_{a} \right|\leftarrow\left| S_{a} \right|$; // Initialise dimensions

1b. ${\forall g\in G: n}_{g}^{1}\leftarrow0$; $\forall p\in P_{g}n_{g,p}^{2}\leftarrow0$; $\forall a\in A,\forall s\in S_{a}:\beta_{a,s}\leftarrow0$;

2. $\forall a\in A,\forall s\in S_{a}: \hat{\beta}_{a,s}\leftarrow\frac{T_{s}}{t_{a}}$; // Compute bounds

3. $\forall g\in G: \hat{n}_{g}^{1}\leftarrow\min_{p\in P_{g}} \left( \frac{\hat{n}_{g,p}^{2}}{\mu_{g,p}^{2}} \right);$ $\forall p\in P_{g}: \hat{n}_{g,p}^{2}\leftarrow\min_{a\in A_{g,p}} \left( \sum_{s} \hat{\beta}_{a,s} \right)$; // Compute bounds

4. ${\hat{\mathcal{N}}}^{\mathrm{DET}}\leftarrow\min_{g\in G} \left( \frac{\hat{n}_{g}^{1}}{\mu_{g}^{1}} \right)$; // Compute bounds

5. $\left( {\hat{\mathcal{N}}}^{\mathrm{STOCH}}, \hat{n}^{1},\hat{n}^{2},\hat{\beta} \right)\leftarrow Capan[DET]({\hat{\mathcal{N}}}^{\mathrm{DET}})$; // Identify theoretical capacity (Alg. 2)

6. $\left( \mathcal{N},n^{1},n^{2},\beta\right)\leftarrow Capan[STOCH]({\hat{\mathcal{N}}}^{\mathrm{STOCH}},\left| I \right|,\mathcal{SL})$; // Identify operational capacity (Alg. 3)

**Alg 2.** $QAHC[DET](\hat{\mathcal{N}})$ // Assess capacity given deterministic durations

1. $evalSLV \leftarrow EvaluateSLV[DET];$ // Designate the SLV evaluator

2. $makeAlloc\leftarrow\mathrm{OptimizeAlloc}[\mathrm{DET}]$; // Designate the allocation algorithm

3. $evalFn\leftarrow\mathrm{EvaluateCaseMix}$; // Designate the objective function

4. $\mathcal{N}\leftarrow\mathrm{BinarySearch}\left( 0,\hat{\mathcal{N}},scale \right)$; // $scale\in(0,1)$. By default, $scale=0.1$

5. return $\mathcal{N},n^{1},n^{2},\beta$;

**Alg 3.** $QAHC[STOCH](\hat{\mathcal{N}},\left| I \right|,\mathcal{SL})$ // Assess capacity given stochastic durations

1. $evalSLV \leftarrow EvaluateSLV[STOCH];$ // Designate the SLV evaluator

2. $makeAlloc\leftarrow\mathrm{OptimizeAlloc}[\mathrm{STOCH}]$; // Designate the allocation algorithm

3. $evalFn\leftarrow\mathrm{Evaluate}$CaseMix; // Designate the objective function

4. $\mathrm{GenerateScenarios}(\left| I \right|)$;

5. $\mathcal{N}\leftarrow\mathrm{BinarySearch}\left( 0,\hat{\mathcal{N}},scale \right)$; // Upper level problem

6. return $\mathbb{N,}n^{1},n^{2},\beta$;

**Alg 4.** $\mathrm{BinarySearch}(lb,ub,scale)$ // Search for the largest value of $\mathcal{N}$ that is achievable

1. $left\leftarrow lb$; $score\_left\leftarrow evalFn(left)$; // Evaluate $\mathcal{N}=left$

2. $right\leftarrow ub$; $score\_right\leftarrow evalFn(right)$; $)$; // Evaluate $\mathcal{N}=right$

3. repeat

4. $next\leftarrow left+scale\times\left( right-left \right)$; // Compute next mid point

5. $score\leftarrow evalFn(next)$; // Evaluate $\mathcal{N}=next$

6. if$\left( score\geq score\_left \right)$ { $left\leftarrow next$; $score\_left\leftarrow score$; }

7. else { $right\leftarrow next;$ $score\_right\leftarrow score$; }

8. until$\left( right-left>prec \right)$; // By default $prec=1$

9. return $left$ or $next$; // Depending upon feasibility

**Alg 5.** $EvaluateCaseMix(npat)$ // General solution evaluation procedure and resource allocation

1. $\mathcal{N}\leftarrow npat$;

2. $\forall g\in G: \{n_{g}^{1}\leftarrow\mu_{g}^{1}\mathcal{N}$;$\forall p\in P_{g}: n_{g,p}^{2}\leftarrow\mu_{g,p}^{2}n_{g}^{1}$}. // Compute case & path mix. Parallel computation.

3. $\beta\leftarrow makeAlloc()$; // Apply $\mathrm{OptimizeAlloc}\left[ \mathrm{DET} \right]or [STOCH]$

4. $SLV\leftarrow evalSLV(\beta)$; // Apply $\mathrm{EvaluateSLV}\left[ DET] or [STOCH \right]$

5. return $score\leftarrow\omega_{1}\mathcal{N}-\omega_{2}SLV$; // By default $\omega_{1}=1$ and $\omega_{2}=\hat{\mathcal{N}}$

**Alg 6.** $\mathrm{EvaluateSLV}\left[ \mathrm{DET} \right]()$ // Service level violation identification & evaluation – deterministic

1. $\forall s\in S:E\left[ U_{s} \right]\leftarrow\sum_{a\in A_{s}} \beta_{a,s}E\left[ t_{a} \right]$;

2. Evaluate equation (32);

3. return $SLV$;

**Alg 7.** $\mathrm{EvaluateSLV}\left[ \mathrm{STOCH} \right]()$ // Service level violation identification & evaluation – stochastic

1. $\forall s\in S: \Pr\left( U_{s}\leq T_{s} \right)\leftarrow SimulateUtil\left( s,\beta\right)$; // Parallel evaluation step

2. Evaluate equation (33);

3. return $SLV$;

**Alg 8.** $\mathrm{SimulateUtil}\left( s,\beta\right)$ // Evaluation of resource utilization across different scenarios

1. $nbOv\leftarrow0;O_{s}^{max}\leftarrow0;$ // # of scenarios resulting in over utilization of space $s$

2. Define $A_{s}=\left\{ a|a\in A,\beta_{a,s}>0 \right\}$;

3. for$\left( i=1..|I| \right)$ // For each scenario

4. $U_{s}^{i}\leftarrow0;$

5a. $\forall a\in A_{s}$: $U_{s}^{i}\leftarrow U_{s}^{i}+\sum_{n\in\left\{ 1..\left\lfloor\beta_{a,s} \right\rfloor\right\}} t_{a}^{i,s,n}+\left( \beta_{a,s}-\left\lfloor\beta_{a,s} \right\rfloor\right)t_{a}^{i,s,\left\lceil\beta_{a,s} \right\rceil}$;

5b. $U_{s}^{i}\leftarrow\sum_{a\in A_{s}} \left[ \sum_{n\in\left\{ 1..\left\lfloor\beta_{a,s} \right\rfloor\right\}} t_{a}^{i,\mathcal{L}_{a,s}[n]}+\left( \beta_{a,s}-\left\lfloor\beta_{a,s} \right\rfloor\right)t_{a}^{i,\mathcal{L}_{a,s}[\bar{n}=\left\lceil\beta_{a,s} \right\rceil]} \right]$;

6. if$\left( U_{s}^{i}>T_{s} \right):\{ nbOv\leftarrow nbOv+1$; $O_{s}^{max}=\max\left( O_{s}^{max},U_{s}^{i}-T_{s} \right);$}

7. $\Pr\left( U_{s}>T_{s} \right)\leftarrow nbOv/\left| I \right|$;

8. $E\left[ U_{s} \right]\leftarrow\sum_{i\in\left\{ 1..\left| I \right| \right\}} U_{s}^{i}/|I|$;

**Alg 9.** $OptimizeAllocation[DET]()$ // Main resource allocation algorithm – deterministic case

1. $\forall s\in S:U_{s}\leftarrow0$; // Reset to zero

2. $\forall a\in A,\forall s\in S_{a}:\beta_{a,s}\leftarrow0$; // Reset to zero

3. for$\left( g=1\ldots|G| \right):$ // For each patient group

4. for$\left( p=1\ldots\left| P_{g} \right| \right)$: if$(n_{g,p}^{2}>0)$ $\mathrm{AllocateGreedy}(g,p,n_{g,p}^{2},\beta)$;

5. $\beta\leftarrow\mathrm{RunMetaH}(pertFn\leftarrow\mathrm{PerturbAlloc},evalFn\leftarrow EvaluateAlloc,\beta)$;

6. return $\beta$;

**Alg 10.** $\mathrm{AllocateGreedy}(g,p,n_{g,p}^{2},\beta)$ // Greedy heuristic algorithm

1. $\forall a\in A_{g,p}:$

2. $remain\leftarrow n_{g,p}^{2}$;

3. $cand\leftarrow\bigcup_{s\in S_{a}} \left( s,T_{s}-E[U_{s}] \right)$; // Spaces ordered according to free time $T_{s}-E[U_{s}]$

4. repeat

5. $\left( free,s \right)\leftarrow cand.first()$;

6. if$\left( \left| cand \right|=1 or free=0 \right)$ $amt\leftarrow remain;$

7. else $amt\leftarrow\min\left( free,E\left[ t_{a} \right]\times remain \right)/E\left[ t_{a} \right]$; // Number of activities to assign

8. if$\left( amt>0 \right)$

9. $\beta_{a,s}\leftarrow amt$; // Make assignment

10. $remain\leftarrow remain-amt$; // Update remain

11. $U_{s}\leftarrow U_{s}+E\left[ t_{a} \right]\times amt$; // Revise time used

12. $cand\leftarrow\{\}$; // Empty the set

13. until($remain=0$);

**Alg 11.** $OptimizeAllocation[STOCH]()$ // Main resource allocation algorithm – stochastic

1. $\beta\leftarrow OptimizeAllocation[DET]()$;

2. $\beta\leftarrow\mathrm{RunMetaH}(pertFn\leftarrow\mathrm{PerturbAlloc},evalFn\leftarrow EvaluateAlloc,\beta)$;

3. return $\beta$;

**Alg 12.** $\mathrm{RunMetaH}$($pertFn,evalFn,\beta$) // Meta-heuristic algorithm

1. Initialise: $t_{I},t_{F},t_{R},maxIter$;

if$\left( SA \right):$ { $acceptFn\leftarrow\mathrm{AcceptChangeMetropolis}$; }

2. if$\left( TA \right):$ { $acceptFn\leftarrow\mathrm{AcceptChangeThreshold}$; $\}$

3. Initialise: $maxNoImpr\leftarrow2; noImpr \leftarrow0$; $step \leftarrow0;$ $stop\leftarrow false;$

4. $\beta^{\mathrm{opt}} \leftarrow\beta$; // Record current allocation as the best

5. while $(!stop) iterate(pertFn,evalFn,stop);$ // Call iterate function

6. return $\beta$;

**Alg 13.** $iterate(pertFn,evalFn,stop)$ // Inner loop of SA and TA meta heuristic

1. $stop\leftarrow\left( t < t_{F} \vee SLV=0 \right);$

2. if$\left( stop \right)$ $\beta\leftarrow\beta^{\mathrm{opt}}$; // Restore best solution

3. else

4. for $\left( iter=1..maxIter \right) pertFn(evalFn)$; // Inner loop

5. $t \leftarrow t_{R} \times t$; // SA: reduce temperature, or TA: reduce threshold

6. $step\leftarrow step+1$; // Increment counter

7. if $\left( casemix.score < bestcasemix.score \right) noImpr\leftarrow noImpr+1;$

8. if $(noImpr > max\_noImpr)$ { $noImpr \leftarrow0$; $\beta\leftarrow\beta^{\mathrm{opt}};$ }

**Alg 14.** $PerturbAlloc()$ // Perturbation procedure for altering the resource allocation decisions

1. $cand\leftarrow\bigcup_{s\in S} \left( s,\Pr\left( U_{s}>T_{s} \right) \right)$; // Spaces ordered according to probability of over-utilization

2. if$\left( \left| cand \right|>0 \right)$

3. $s\leftarrow cand\left[ 1 \right].first()$; // Consider the most over utilized space

4. $choose\leftarrow Unif\left( 1,\left| A_{s} \right| \right)$; // Select an activity, where $A_{s}=\left\{ a|a\in A,\beta_{a,s}>0 \right\}$

5. $a=A_{s}[choose]$; // The selected activity

6. if$\left( \left| S_{a} \right|>1 \right)$ // Options exist. Move the activity to another space

7. $cand2\leftarrow\bigcup_{s'\in S\backslash\{s\}} \left( s',U_{s^{'}} \right)$; // Spaces ordered by time used

8. $s^{'}\leftarrow cand2\left[ 1 \right].first();$ // Choose the least utilized space

9. $amt=Unif\left( 0,1 \right)\times\beta_{a,s};$ // Amount to be removed

10. if$(amt>0)$

11. $\beta_{a,s}\leftarrow\beta_{a,s}-amt$; $\beta_{a,s^{'}}\leftarrow\beta_{a,s^{'}}+amt$; // Revise the allocation

13. $prevScore\leftarrow score;currScore\leftarrow evalFn();$ // Evaluate the new allocation

14. if$\left( acceptFn(t,prevScore,currScore \right))$ $\beta^{\mathrm{opt}} \leftarrow\beta;$ // Update best

15. else { $\beta_{a,s}\leftarrow\beta_{a,s}+amt$; $\beta_{a,s^{'}}\leftarrow\beta_{a,s^{'}}-amt$; } // Undo perturbation

**Alg 15.** $\mathrm{AcceptChangeMetropolis}(temp,prevScore,currScore)$ // SA acceptance procedure

1. if$\left( currScore>prevScore \right)$ return $true$;

2. else return$\left( Unif\left( 0,1 \right)<e^{\left( currScore-prevScore \right)/temp} \right)$;

**Alg 16.** $AcceptChangeThreshold(threshold, prevScore,currScore)$ // TA acceptance procedure

1. return $\left( currScore\geq prevScore-threshold \right)$;
