# Supplementary material for: A stochastic programming approach to perform hospital capacity assessments
Source: PLoS One. 2023 Nov 9;18(11):e0287980. doi: 10.1371/journal.pone.0287980 (PMC10635551; doi:10.1371/journal.pone.0287980)
Supplement: S1 Data — (DOCX) [file pone.0287980.s002.docx]

**CASE STUDY DETAILS**

| # | SPECIALTY | DESCRIPTION | #PATHS |
| --- | --- | --- | --- |
| 1 | ASU | Acute Surgical | 2 |
| 2 | BE | Breast and Endocrine | 4 |
| 3 | COLO | Colorectal | 4 |
| 4 | CSU | Cardia Surgery | 4 |
| 5 | DENT | Dental | 1 |
| 6 | ENT | Ear, Nose & Throat | 4 |
| 7 | FMAX | Faciomaxillary | 4 |
| 8 | GYN | Gynaecology | 2 |
| 9 | GAST | Gastroenterology | 1 |
| 10 | HPB | Hepatobillary | 4 |
| 11 | LTPT | Liver Transplant | 4 |
| 12 | NSUR | Neuro Surgery | 4 |
| 13 | OPHT | Opthalmology | 2 |
| 14 | ORTH | Orthopaedic | 4 |
| 15 | PLAS | Plastics | 4 |
| 16 | RESP | Respiratory | 1 |
| 17 | RTPT | Renal Transplant | 4 |
| 18 | TRMA | Trauma | 4 |
| 19 | UGI | Upper Gastro Intestinal | 4 |
| 20 | UROL | Urology | 4 |
| 21 | VASC | Vascular | 4 |
|  |  |  |  |
| 22 | INFD | Infectious Disease | na |
| 23 | ICU | Intensive Care | na |
| 24 | OHU | Oncology | na |
| 25 | OTU | Operating Theatre | na |
| 26 | REN | Renal | na |
| 27 | ROU | Radiation Oncology | na |
| 28 | SCU | Surgical Care | na |

| AREA | #SPACES | ACTIVITY | SPECIALTY |
| --- | --- | --- | --- |
| 1A | 20 | med | Undefined |
| 1B | 20 | med | Undefined |
| 1C | 24 | postop | VASC |
| 1D | 26 | postop | FMAX, PLAS, ENT, DENT |
| 1H | 10 | im | IMAGING |
| 1L | 10 | im | IMAGING |
| 2A | 24 | postop | ORTH |
| 2A-HD | 4 | postop | TRMA |
| 2B | 24 | postop | ORTH |
| 2C | 32 | postop | NSUR |
| 2C-HD | 8 | postop | NSUR |
| 2D | 24 | postop | RHEU, RESP |
| 2D-HD | 4 | postop | RESP |
| 2E | 29 | postop | OHU, ROU |
| 3A | 15 | ic | ICU |
| 3B | 10 | ic | ICU |
| 3C | 28 | postop | CSU |
| 3D | 20 | postop | CSU |
| 3E | 20 | postop | CSU |
| 3F | 20 | preop | SURGICAL CARE |
| 3H | 20 | pac | SURGICAL CARE |
| 3L-A | 4 | sur | OTU |
| 3L-B | 3 | sur | OTU |
| 3L-C | 4 | sur | OTU |
| 3L-D | 5 | sur | OTU |
| 3L-E | 3 | sur | OTU |
| 4A | 19 | postop | UROL |
| 4B-R | 14 | postop | REN |
| 4B-T | 16 | postop | LTPT |
| 4C | 28 | postop | HPB, GYN, ASU |
| 4D | 28 | postop | BE, COLO, UGI, PLAS, ASU |
| 4E | 26 | postop | COLO |
| 5A | 28 | med | OPHT |
| 5B | 24 | postop, med | Undefined |
| 5C | 24 | med | INFD |
| 5D | 24 | postop | INFD |

**PATHWAY ACTIVITIES AND DURATIONS**

Each duration is a random variable with a set of breakpoints, and a set of probabilities for values occurring within the given intervals.

Group::AS|Path::AS#1,

PREOP(SCU),Duration=[{0,1.5,3,4.5,6,7.5,9,10.5,12},{0.22,0.18,0.16,0.3,0.06,0.02,0.04,0.02}]

SUR(ASU),Duration=[{0,1,2,3,4,5,6,7},{0.17,0.44,0.23,0.11,0.04,0.01,0}]

PAC(SCU),Duration=[{0,1,2,3,4,5,6,7,8,9,10,11,12},{0.019,0.037,0.148,0.185,0.213,0.185,0.083,0.056,0.028,0.028,0.009,0.009}]

POSTOP(ASU),Duration=[{0,3,6,9,12,15,18,21,24,27,30,33,36,39,42},{0.569,0.257,0.097,0.036,0.022,0.009,0.004,0.002,0.001,0,0.001,0,0.001,0.001}]

Group::AS|Path::AS#2,

PREOP(SCU),Duration=[{0,1.5,3,4.5,6,7.5,9,10.5,12},{0.22,0.18,0.16,0.3,0.06,0.02,0.04,0.02}]

SUR(ASU),Duration=[{0,1,2,3,4,5,6,7},{0.17,0.44,0.23,0.11,0.04,0.01,0}]

IC(ICU),Duration=[{0,1,2,3,4,5,6,7,8,9,10,12,13},{0.4,0.2,0.1,0.02,0.06,0.02,0.06,0,0,0.04,0,0.1}]

POSTOP(ASU),Duration=[{0,3,6,9,12,15,18,21,24,27,30,33,36,39,42},{0.569,0.257,0.097,0.036,0.022,0.009,0.004,0.002,0.001,0,0.001,0,0.001,0.001}]

Group::BE|Path::BE#1,

PREOP(SCU),Duration=[{0,1.5,3,4.5,6,7.5,9,10.5,12,13.5,15,27,28.5},{0.076,0.359,0.136,0.153,0.138,0.062,0.051,0.015,0.004,0.002,0,0.004}]

SUR(BE),Duration=[{0,1,2,3,4,5},{0.15,0.44,0.31,0.07,0.03}]

PAC(SCU),Duration=[{0,1,2,3,4,5,6,7,8,9,10,11,12},{0.015,0.013,0.07,0.32,0.322,0.134,0.064,0.031,0.01,0.01,0.003,0.008}]

POSTOP(BE),Duration=[{0,2,4,6,8,10,12,14,16,18,20},{0.704,0.153,0.059,0.035,0.023,0.008,0.005,0.005,0.006,0.002}]

Group::BE|Path::BE#2,

PREOP(SCU),Duration=[{0,1.5,3,4.5,6,7.5,9,10.5,12,13.5,15,27,28.5},{0.076,0.359,0.136,0.153,0.138,0.062,0.051,0.015,0.004,0.002,0,0.004}]

SUR(BE),Duration=[{0,1,2,3,4,5},{0.15,0.44,0.31,0.07,0.03}]

IC(ICU),Duration=[{0,1,2,3,4,10,11},{0.33,0.44,0.11,0.06,0,0.06}]

POSTOP(BE),Duration=[{0,2,4,6,8,10,12,14,16,18,20},{0.704,0.153,0.059,0.035,0.023,0.008,0.005,0.005,0.006,0.002}]

Group::BE|Path::BE#3,

PREOP(SCU),Duration=[{0,1.5,3,4.5,6,7.5,9,10.5,12,13.5,15,27,28.5},{0.076,0.359,0.136,0.153,0.138,0.062,0.051,0.015,0.004,0.002,0,0.004}]

SUR(BE),Duration=[{0,1,2,3,4,5,6},{0.17,0.36,0.25,0.11,0.08,0.03}]

PAC(SCU),Duration=[{0,1,2,3,4,5,6,7,8,9,10,11,12},{0.015,0.013,0.07,0.32,0.322,0.134,0.064,0.031,0.01,0.01,0.003,0.008}]

POSTOP(BE),Duration=[{0,2,4,6,8,10,12,14,16,18,20},{0.704,0.153,0.059,0.035,0.023,0.008,0.005,0.005,0.006,0.002}]

Group::BE|Path::BE#4,

PREOP(SCU),Duration=[{0,1.5,3,4.5,6,7.5,9,10.5,12,13.5,15,27,28.5},{0.076,0.359,0.136,0.153,0.138,0.062,0.051,0.015,0.004,0.002,0,0.004}]

SUR(BE),Duration=[{0,1,2,3,4,5,6},{0.17,0.36,0.25,0.11,0.08,0.03}]

IC(ICU),Duration=[{0,1,2,3,4,10,11},{0.33,0.44,0.11,0.06,0,0.06}]

POSTOP(BE),Duration=[{0,2,4,6,8,10,12,14,16,18,20},{0.704,0.153,0.059,0.035,0.023,0.008,0.005,0.005,0.006,0.002}]

Group::COLO|Path::COLO#1,

PREOP(SCU),Duration=[{0,1.5,3,4.5,6,7.5,9,10.5,12,13.5,15,16.5},{0.069,0.444,0.113,0.16,0.127,0.047,0.018,0.015,0.004,0,0.003}]

SUR(COLO),Duration=[{0,2,4,6,8,10,12,14},{0.42,0.21,0.27,0.05,0.02,0.01,0.02}]

PAC(SCU),Duration=[{0,1,2,3,4,5,6,7,8,9,10,11,12,13},{0.014,0.01,0.096,0.111,0.096,0.12,0.13,0.173,0.154,0.048,0.01,0.005,0.033}]

POSTOP(COLO),Duration=[{0,3,6,9,12,15,18,21,24,27,30,33,36,39},{0.446,0.231,0.132,0.062,0.054,0.019,0.015,0.007,0.004,0.006,0.003,0.007,0.014}]

Group::COLO|Path::COLO#2,

PREOP(SCU),Duration=[{0,1.5,3,4.5,6,7.5,9,10.5,12,13.5,15,16.5},{0.069,0.444,0.113,0.16,0.127,0.047,0.018,0.015,0.004,0,0.003}]

SUR(COLO),Duration=[{0,2,4,6,8,10,12,14},{0.42,0.21,0.27,0.05,0.02,0.01,0.02}]

IC(ICU),Duration=[{0,1,2,3,4,5,6,7,13,15},{0.456,0.263,0.123,0.053,0,0.035,0.035,0,0.035}]

POSTOP(COLO),Duration=[{0,3,6,9,12,15,18,21,24,27,30,33,36,39},{0.446,0.231,0.132,0.062,0.054,0.019,0.015,0.007,0.004,0.006,0.003,0.007,0.014}]

Group::COLO|Path::COLO#3,

PREOP(SCU),Duration=[{0,1.5,3,4.5,6,7.5,9,10.5,12,13.5,15,16.5},{0.069,0.444,0.113,0.16,0.127,0.047,0.018,0.015,0.004,0,0.003}]

SUR(COLO),Duration=[{0,1,2,3,4,5,6},{0.27,0.36,0.22,0.08,0.04,0.03}]

PAC(SCU),Duration=[{0,1,2,3,4,5,6,7,8,9,10,11,12,13},{0.014,0.01,0.096,0.111,0.096,0.12,0.13,0.173,0.154,0.048,0.01,0.005,0.033}]

POSTOP(COLO),Duration=[{0,3,6,9,12,15,18,21,24,27,30,33,36,39},{0.446,0.231,0.132,0.062,0.054,0.019,0.015,0.007,0.004,0.006,0.003,0.007,0.014}]

Group::COLO|Path::COLO#4,

PREOP(SCU),Duration=[{0,1.5,3,4.5,6,7.5,9,10.5,12,13.5,15,16.5},{0.069,0.444,0.113,0.16,0.127,0.047,0.018,0.015,0.004,0,0.003}]

SUR(COLO),Duration=[{0,1,2,3,4,5,6},{0.27,0.36,0.22,0.08,0.04,0.03}]

IC(ICU),Duration=[{0,1,2,3,4,5,6,7,13,15},{0.456,0.263,0.123,0.053,0,0.035,0.035,0,0.035}]

POSTOP(COLO),Duration=[{0,3,6,9,12,15,18,21,24,27,30,33,36,39},{0.446,0.231,0.132,0.062,0.054,0.019,0.015,0.007,0.004,0.006,0.003,0.007,0.014}]

Group::CARD|Path::CARD#1,

PREOP(SCU),Duration=[{0,1.5,3,4.5,6,7.5,9,10.5,12,13.5},{0.083,0.189,0.226,0.258,0.138,0.037,0.041,0.023,0.005}]

SUR(CSU),Duration=[{0,1,2,3,4,5,6,7,8,9},{0.01,0.09,0.09,0.24,0.36,0.13,0.05,0.01,0.02}]

PAC(SCU),Duration=[{0,1,2,3,4,5,6,7,8,9,10,11,12,13},{0.008,0.015,0.036,0.107,0.304,0.307,0.116,0.05,0.023,0.015,0.008,0.005,0.006}]

POSTOP(CSU),Duration=[{0,3,6,9,12,15,18,21,24,27,30},{0.222,0.539,0.162,0.041,0.014,0.006,0.006,0.003,0.002,0.005}]

Group::CARD|Path::CARD#2,

PREOP(SCU),Duration=[{0,1.5,3,4.5,6,7.5,9,10.5,12,13.5},{0.083,0.189,0.226,0.258,0.138,0.037,0.041,0.023,0.005}]

SUR(CSU),Duration=[{0,1,2,3,4,5,6,7,8,9},{0.01,0.09,0.09,0.24,0.36,0.13,0.05,0.01,0.02}]

IC(ICU),Duration=[{0,1,2,3,4,5,6,7,8,9,10},{0.513,0.157,0.093,0.081,0.042,0.021,0.03,0.025,0.021,0.017}]

POSTOP(CSU),Duration=[{0,3,6,9,12,15,18,21,24,27,30},{0.222,0.539,0.162,0.041,0.014,0.006,0.006,0.003,0.002,0.005}]

Group::CARD|Path::CARD#3,

PREOP(SCU),Duration=[{0,1.5,3,4.5,6,7.5,9,10.5,12,13.5},{0.083,0.189,0.226,0.258,0.138,0.037,0.041,0.023,0.005}]

SUR(CSU),Duration=[{0,1,2,3,4,5,6,7,8,9,10},{0.05,0.2,0.12,0.18,0.21,0.1,0.04,0.04,0.02,0.04}]

PAC(SCU),Duration=[{0,1,2,3,4,5,6,7,8,9,10,11,12,13},{0.008,0.015,0.036,0.107,0.304,0.307,0.116,0.05,0.023,0.015,0.008,0.005,0.006}]

POSTOP(CSU),Duration=[{0,3,6,9,12,15,18,21,24,27,30},{0.222,0.539,0.162,0.041,0.014,0.006,0.006,0.003,0.002,0.005}]

Group::CARD|Path::CARD#4,

PREOP(SCU),Duration=[{0,1.5,3,4.5,6,7.5,9,10.5,12,13.5},{0.083,0.189,0.226,0.258,0.138,0.037,0.041,0.023,0.005}]

SUR(CSU),Duration=[{0,1,2,3,4,5,6,7,8,9,10},{0.05,0.2,0.12,0.18,0.21,0.1,0.04,0.04,0.02,0.04}]

IC(ICU),Duration=[{0,1,2,3,4,5,6,7,8,9,10},{0.513,0.157,0.093,0.081,0.042,0.021,0.03,0.025,0.021,0.017}]

POSTOP(CSU),Duration=[{0,3,6,9,12,15,18,21,24,27,30},{0.222,0.539,0.162,0.041,0.014,0.006,0.006,0.003,0.002,0.005}]

Group::DENT|Path::DENT#1,

PREOP(SCU),Duration=Normal(mean=60,stddev=15)

SUR(DENT),Duration=Normal(mean=80.4,stddev=30)

PAC(SCU),Duration=Normal(mean=184.2,stddev=15)

POSTOP(DENT),Duration=Normal(mean=492.6,stddev=180)

Group::ENT|Path::ENT#1,

PREOP(SCU),Duration=[{0,1.5,3,4.5,6,7.5,9,10.5,12,13.5,15},{0.13,0.28,0.14,0.15,0.14,0.08,0.04,0.03,0.01,0}]

SUR(ENT),Duration=[{0,1.5,3,4.5,6,7.5,9,10.5,12,13.5,15},{0.55,0.18,0.08,0.05,0.03,0.02,0.02,0.01,0.02,0.04}]

PAC(SCU),Duration=[{0,2,4,6,8,10,12,14,16,18,20},{0.06,0.39,0.19,0.13,0.1,0.04,0.03,0.02,0.03,0.01}]

POSTOP(ENT),Duration=[{0,3,6,9,12,15,18,21,24,27,30},{0.68,0.162,0.048,0.028,0.036,0.004,0.016,0.006,0.001,0.019}]

Group::ENT|Path::ENT#2,

PREOP(SCU),Duration=[{0,1.5,3,4.5,6,7.5,9,10.5,12,13.5,15},{0.13,0.28,0.14,0.15,0.14,0.08,0.04,0.03,0.01,0}]

SUR(ENT),Duration=[{0,1.5,3,4.5,6,7.5,9,10.5,12,13.5,15},{0.55,0.18,0.08,0.05,0.03,0.02,0.02,0.01,0.02,0.04}]

IC(ICU),Duration=[{0,1,2,3,4,5,6,7,8,9,10},{0.604,0.209,0.033,0.055,0.022,0.033,0.033,0,0,0.011}]

POSTOP(ENT),Duration=[{0,3,6,9,12,15,18,21,24,27,30},{0.68,0.162,0.048,0.028,0.036,0.004,0.016,0.006,0.001,0.019}]

Group::ENT|Path::ENT#3,

PREOP(SCU),Duration=[{0,1.5,3,4.5,6,7.5,9,10.5,12,13.5,15},{0.13,0.28,0.14,0.15,0.14,0.08,0.04,0.03,0.01,0}]

SUR(ENT),Duration=[{0,1,2,3,4},{0.29,0.5,0.17,0.04}]

PAC(SCU),Duration=[{0,2,4,6,8,10,12,14,16,18,20},{0.06,0.39,0.19,0.13,0.1,0.04,0.03,0.02,0.03,0.01}]

POSTOP(ENT),Duration=[{0,3,6,9,12,15,18,21,24,27,30},{0.68,0.162,0.048,0.028,0.036,0.004,0.016,0.006,0.001,0.019}]

Group::ENT|Path::ENT#4,

PREOP(SCU),Duration=[{0,1.5,3,4.5,6,7.5,9,10.5,12,13.5,15},{0.13,0.28,0.14,0.15,0.14,0.08,0.04,0.03,0.01,0}]

SUR(ENT),Duration=[{0,1,2,3,4},{0.29,0.5,0.17,0.04}]

IC(ICU),Duration=[{0,1,2,3,4,5,6,7,8,9,10},{0.604,0.209,0.033,0.055,0.022,0.033,0.033,0,0,0.011}]

POSTOP(ENT),Duration=[{0,3,6,9,12,15,18,21,24,27,30},{0.68,0.162,0.048,0.028,0.036,0.004,0.016,0.006,0.001,0.019}]

Group::FMAX|Path::FMAX#1,

PREOP(SCU),Duration=[{0,2,4,6,8,10,12,14},{0.18,0.17,0.21,0.2,0.17,0.05,0.02}]

SUR(FMAX),Duration=[{0,1,2,3,4,5,6,7,8,9},{0.3,0.49,0.13,0.02,0.02,0.01,0.01,0.01,0.01}]

PAC(SCU),Duration=[{0,1,2,3,4,5,6,7,8,9,10},{0.03,0.03,0.172,0.291,0.201,0.104,0.082,0.052,0.03,0.0071}]

POSTOP(FMAX),Duration=[{0,1,2,3,4,5,6,7,8,9,10,11},{0.32,0.365,0.137,0.102,0.018,0.018,0.004,0.004,0.014,0.007,0.011}]

Group::FMAX|Path::FMAX#2,

PREOP(SCU),Duration=[{0,2,4,6,8,10,12,14},{0.18,0.17,0.21,0.2,0.17,0.05,0.02}]

SUR(FMAX),Duration=[{0,1,2,3,4,5,6,7,8,9},{0.3,0.49,0.13,0.02,0.02,0.01,0.01,0.01,0.01}]

IC(ICU),Duration=[{0,1,2,3,4,5,6,7,8},{0.57,0.15,0.12,0.04,0,0.04,0.04,0.04}]

POSTOP(FMAX),Duration=[{0,1,2,3,4,5,6,7,8,9,10,11},{0.32,0.365,0.137,0.102,0.018,0.018,0.004,0.004,0.014,0.007,0.011}]

Group::FMAX|Path::FMAX#3,

PREOP(SCU),Duration=[{0,2,4,6,8,10,12,14},{0.18,0.17,0.21,0.2,0.17,0.05,0.02}]

SUR(FMAX),Duration=[{0,0.5,1,1.5,2,2.5,3,3.5,4,4.5,5},{0.02,0.17,0.37,0.23,0.12,0.04,0.02,0.02,0.01,0.01}]

PAC(SCU),Duration=[{0,1,2,3,4,5,6,7,8,9,10},{0.03,0.03,0.172,0.291,0.201,0.104,0.082,0.052,0.03,0.007}]

POSTOP(FMAX),Duration=[{0,1,2,3,4,5,6,7,8,9,10,11},{0.32,0.365,0.137,0.102,0.018,0.018,0.004,0.004,0.014,0.007,0.011}]

Group::FMAX|Path::FMAX#4,

PREOP(SCU),Duration=[{0,2,4,6,8,10,12,14},{0.18,0.17,0.21,0.2,0.17,0.05,0.02}]

SUR(FMAX),Duration=[{0,0.5,1,1.5,2,2.5,3,3.5,4,4.5,5},{0.02,0.17,0.37,0.23,0.12,0.04,0.02,0.02,0.01,0.01}]

IC(ICU),Duration=[{0,1,2,3,4,5,6,7,8},{0.57,0.15,0.12,0.04,0,0.04,0.04,0.04}]

POSTOP(FMAX),Duration=[{0,1,2,3,4,5,6,7,8,9,10,11},{0.32,0.365,0.137,0.102,0.018,0.018,0.004,0.004,0.014,0.007,0.011}]

Group::GAST|Path::GAST#1,

SUR(GAST),Duration=[{0,0.5,1,1.5,2,2.5,3,3.5,4,4.5,5},{0.11,0.46,0.22,0.12,0.05,0.01,0.01,0,0.01,0.01}]

PAC(SCU),Duration=[{1,2,3,4,5,6,7,8,9,10},{0.087,0.217,0.326,0.087,0.109,0.087,0.043,0.022,0.022}]

POSTOP(GAST),Duration=[{0,3,6,9,12,15,18,21,24,27,30},{0.673,0.146,0.083,0.044,0.018,0.013,0.011,0.007,0.002,0.002}]

Group::GAST|Path::GAST#2,

SUR(GAST),Duration=[{0,0.5,1,1.5,2,2.5,3,3.5,4,4.5,5},{0.11,0.46,0.22,0.12,0.05,0.01,0.01,0,0.01,0.01}]

POSTOP(GAST),Duration=[{0,1,2,3,4,5,6,7,8,9,10},{0.294,0.196,0.137,0.235,0.039,0.039,0,0.039,0.02,0.001}]

Group::GYN|Path::GYN#1,

PREOP(SCU),Duration=Normal(mean=21,stddev=15)

SUR(GYN),Duration=Normal(mean=28.8,stddev=30)

POSTOP(GYN),Duration=Normal(mean=759.6,stddev=180)

Group::HPB|Path::HPB#1,

PREOP(SCU),Duration=[{0,1.5,3,4.5,6,7.5,9,10.5,12,13.5,15},{0.074,0.382,0.14,0.162,0.111,0.048,0.046,0.026,0.009,0.002}]

SUR(HPB),Duration=[{0,1,2,3,4,5,6,7,8,9},{0.05,0.37,0.18,0.12,0.11,0.06,0.07,0.03,0.01}]

PAC(SCU),Duration=[{1,2,3,4,5,6,7,8,9,10,11,12,13,14},{0.01,0.05,0.14,0.21,0.21,0.13,0.1,0.05,0.05,0.02,0.01,0,0.02}]

POSTOP(HPB),Duration=[{0,5,10,15,20,25,30,35,40,45,50,55,60,65,70},{0.612,0.225,0.088,0.03,0.017,0.009,0.005,0.003,0.003,0.002,0.001,0.001,0.001,0.002}]

Group::HPB|Path::HPB#2,

PREOP(SCU),Duration=[{0,1.5,3,4.5,6,7.5,9,10.5,12,13.5,15},{0.074,0.382,0.14,0.162,0.111,0.048,0.046,0.026,0.009,0.002}]

SUR(HPB),Duration=[{0,1,2,3,4,5,6,7,8,9},{0.05,0.37,0.18,0.12,0.11,0.06,0.07,0.03,0.01}]

IC(ICU),Duration=[{0,1,2,3,4,5,6,7,8,9,10},{0.685,0.203,0.056,0.021,0.007,0.007,0.007,0.007,0.007,0}]

POSTOP(HPB),Duration=[{0,5,10,15,20,25,30,35,40,45,50,55,60,65,70},{0.612,0.225,0.088,0.03,0.017,0.009,0.005,0.003,0.003,0.002,0.001,0.001,0.001,0.002}]

Group::HPB|Path::HPB#3,

PREOP(SCU),Duration=[{0,1.5,3,4.5,6,7.5,9,10.5,12,13.5,15},{0.074,0.382,0.14,0.162,0.111,0.048,0.046,0.026,0.009,0.002}]

SUR(HPB),Duration=[{0,0.75,1.5,2.25,3,3.75,4.5,5.25,6},{0.074,0.213,0.331,0.228,0.051,0.029,0.044,0.03}]

PAC(SCU),Duration=[{1,2,3,4,5,6,7,8,9,10,11,12,13,14},{0.01,0.05,0.14,0.21,0.21,0.13,0.1,0.05,0.05,0.02,0.01,0,0.02}]

POSTOP(HPB),Duration=[{0,5,10,15,20,25,30,35,40,45,50,55,60,65,70},{0.612,0.225,0.088,0.03,0.017,0.009,0.005,0.003,0.003,0.002,0.001,0.001,0.001,0.002}]

Group::HPB|Path::HPB#4,

PREOP(SCU),Duration=[{0,1.5,3,4.5,6,7.5,9,10.5,12,13.5,15},{0.074,0.382,0.14,0.162,0.111,0.048,0.046,0.026,0.009,0.002}]

SUR(HPB),Duration=[{0,0.75,1.5,2.25,3,3.75,4.5,5.25,6},{0.074,0.213,0.331,0.228,0.051,0.029,0.044,0.03}]

IC(ICU),Duration=[{0,1,2,3,4,5,6,7,8,9,10},{0.685,0.203,0.056,0.021,0.007,0.007,0.007,0.007,0.007,0}]

POSTOP(HPB),Duration=[{0,5,10,15,20,25,30,35,40,45,50,55,60,65,70},{0.612,0.225,0.088,0.03,0.017,0.009,0.005,0.003,0.003,0.002,0.001,0.001,0.001,0.002}]

Group::LTPT|Path::LTPT#1,

PREOP(SCU),Duration=[{0,1,2,3,4,5,6,7},{0,0,0.125,0.5,0.125,0.125,0.125}]

SUR(LTPT),Duration=[{0,0.5,1,1.5,2,2.5,3},{0.13,0.53,0.2,0,0.07,0.07}]

PAC(SCU),Duration=[{2,3,7,8,9,10,13,14},{0.17,0,0.17,0.32,0.17,0,0.17}]

POSTOP(LTPT),Duration=[{0,3,6,9,12,15,18,21,24,27,30,33,36},{0.482,0.213,0.15,0.028,0.028,0.028,0.02,0.024,0.016,0,0,0.011}]

Group::LTPT|Path::LTPT#2,

PREOP(SCU),Duration=[{0,1,2,3,4,5,6,7},{0,0,0.125,0.5,0.125,0.125,0.125}]

SUR(LTPT),Duration=[{0,0.5,1,1.5,2,2.5,3},{0.13,0.53,0.2,0,0.07,0.07}]

IC(ICU),Duration=[{0,1,2,3,4,5,6,7,8,9,10},{0.244,0.356,0.178,0.111,0.022,0,0,0.022,0.022,0.045}]

POSTOP(LTPT),Duration=[{0,3,6,9,12,15,18,21,24,27,30,33,36},{0.482,0.213,0.15,0.028,0.028,0.028,0.02,0.024,0.016,0,0,0.011}]

Group::LTPT|Path::LTPT#3,

PREOP(SCU),Duration=[{0,1,2,3,4,5,6,7},{0,0,0.125,0.5,0.125,0.125,0.125}]

SUR(LTPT),Duration=[{0,1.5,3,4.5,6,7.5,9,10.5,12,13.5,15,16.5},{0.06,0.22,0.12,0.01,0.09,0.32,0.09,0.03,0,0.04,0.02}]

PAC(SCU),Duration=[{2,3,7,8,9,10,13,14},{0.17,0,0.17,0.33,0.17,0,0.17}]

POSTOP(LTPT),Duration=[{0,3,6,9,12,15,18,21,24,27,30,33,36},{0.482,0.213,0.15,0.028,0.028,0.028,0.02,0.024,0.016,0,0,0.011}]

Group::LTPT|Path::LTPT#4,

PREOP(SCU),Duration=[{0,1,2,3,4,5,6,7},{0,0,0.125,0.5,0.125,0.125,0.125}]

SUR(LTPT),Duration=[{0,1.5,3,4.5,6,7.5,9,10.5,12,13.5,15,16.5},{0.06,0.22,0.12,0.01,0.09,0.32,0.09,0.03,0,0.04,0.02}]

IC(ICU),Duration=[{0,1,2,3,4,5,6,7,8,9,10},{0.244,0.356,0.178,0.111,0.022,0,0,0.022,0.022,0.045}]

POSTOP(LTPT),Duration=[{0,3,6,9,12,15,18,21,24,27,30,33,36},{0.482,0.213,0.15,0.028,0.028,0.028,0.02,0.024,0.016,0,0,0.011}]

Group::NSUR|Path::NSUR#1,

PREOP(SCU),Duration=[{0,2,4,6,8,10,12,14},{0.274,0.346,0.238,0.097,0.028,0.013,0.004}]

SUR(NSUR),Duration=[{0,1,2,3,4,5,6,7,8,9},{0.08,0.26,0.31,0.17,0.08,0.04,0.02,0.02,0.02}]

PAC(SCU),Duration=[{0,2,4,6,8,10,12,14,16},{0.034,0.142,0.381,0.236,0.139,0.043,0.014,0.011}]

POSTOP(NSUR),Duration=[{0,5,10,15,20,25,30,35,40,45,50,55,60},{0.592,0.23,0.091,0.027,0.027,0.013,0.007,0.006,0.001,0.001,0.001,0.004}]

Group::NSUR|Path::NSUR#2,

PREOP(SCU),Duration=[{0,2,4,6,8,10,12,14},{0.274,0.346,0.238,0.097,0.028,0.013,0.004}]

SUR(NSUR),Duration=[{0,1,2,3,4,5,6,7,8,9},{0.08,0.26,0.31,0.17,0.08,0.04,0.02,0.02,0.02}]

IC(ICU),Duration=[{0,2,4,6,8,10,12,14,16,18,20,22,24},{0.421,0.197,0.092,0.061,0.079,0.039,0.022,0.026,0.022,0.018,0.013,0.01}]

POSTOP(NSUR),Duration=[{0,5,10,15,20,25,30,35,40,45,50,55,60},{0.592,0.23,0.091,0.027,0.027,0.013,0.007,0.006,0.001,0.001,0.001,0.004}]

Group::NSUR|Path::NSUR#3,

PREOP(SCU),Duration=[{0,2,4,6,8,10,12,14},{0.274,0.346,0.238,0.097,0.028,0.013,0.004}]

SUR(NSUR),Duration=[{0,1,2,3,4,5,6,7,8},{0.03,0.46,0.37,0.08,0.03,0.02,0.01,0}]

PAC(SCU),Duration=[{0,2,4,6,8,10,12,14,16},{0.034,0.142,0.381,0.236,0.139,0.043,0.014,0.011}]

POSTOP(NSUR),Duration=[{0,5,10,15,20,25,30,35,40,45,50,55,60},{0.592,0.23,0.091,0.027,0.027,0.013,0.007,0.006,0.001,0.001,0.001,0.004}]

Group::NSUR|Path::NSUR#4,

PREOP(SCU),Duration=[{0,2,4,6,8,10,12,14},{0.274,0.346,0.238,0.097,0.028,0.013,0.004}]

SUR(NSUR),Duration=[{0,1,2,3,4,5,6,7,8},{0.03,0.46,0.37,0.08,0.03,0.02,0.01,0}]

IC(ICU),Duration=[{0,2,4,6,8,10,12,14,16,18,20,22,24},{0.421,0.197,0.092,0.061,0.079,0.039,0.022,0.026,0.022,0.018,0.013,0.01}]

POSTOP(NSUR),Duration=[{0,5,10,15,20,25,30,35,40,45,50,55,60},{0.592,0.23,0.091,0.027,0.027,0.013,0.007,0.006,0.001,0.001,0.001,0.004}]

Group::OPHT|Path::OPHT#1,

PREOP(SCU),Duration=[{0,1.5,3,4.5,6,7.5,9,10.5,12,13.5,15},{0.007,0.054,0.384,0.351,0.14,0.046,0.012,0.004,0.001,0.001}]

SUR(OPHT),Duration=[{0,0.5,1,1.5,2,2.5,3,3.5,4,4.5,5},{0.03,0.16,0.24,0.25,0.15,0.06,0.07,0.01,0.02,0.01}]

PAC(SCU),Duration=[{0,1,2,3,4,5,6,7,8,9,10},{0.048,0.229,0.276,0.17,0.114,0.048,0.057,0.029,0.019,0.01}]

POSTOP(OPHT),Duration=[{0,3,6,9,12,15,18,21,24,27,30},{0.602,0.218,0.077,0.035,0.018,0.024,0.006,0.006,0.003,0.011}]

Group::OPHT|Path::OPHT#2,

PREOP(SCU),Duration=[{0,1.5,3,4.5,6,7.5,9,10.5,12,13.5,15},{0.007,0.054,0.384,0.351,0.14,0.046,0.012,0.004,0.001,0.001}]

SUR(OPHT),Duration=[{0,0.4,0.8,1.2,1.6,2,2.4,2.8,3.2,3.6,4},{0.19,0.53,0.13,0.08,0.03,0.02,0.01,0.002,0.004,0.004}]

PAC(SCU),Duration=[{0,1,2,3,4,5,6,7,8,9,10},{0.048,0.229,0.276,0.17,0.114,0.048,0.057,0.029,0.019,0.01}]

POSTOP(OPHT),Duration=[{0,3,6,9,12,15,18,21,24,27,30},{0.602,0.218,0.077,0.035,0.018,0.024,0.006,0.006,0.003,0.011}]

Group::ORTH|Path::ORTH#1,

PREOP(SCU),Duration=[{0,2,4,6,8,10,12,14,16,18,20,22,24,26,28},{0.1654,0.2266,0.1966,0.1773,0.1416,0.0686,0.0193,0.0023,0.0011,0,0.0006,0,0,0.0006}]

SUR(ORTH),Duration=[{0,0.75,1.5,2.25,3,3.375,4.5,5.25,6,6.75,7.5,8.25,9},{0.11,0.26,0.23,0.17,0.1,0.06,0.03,0.02,0,0,0,0.02}]

PAC(SCU),Duration=[{0,2,4,6,8,10,12,14,16,18,20},{0.04,0.23,0.36,0.21,0.08,0.03,0.01,0.01,0.01,0.02}]

POSTOP(ORTH),Duration=[{0,5,10,15,20,25,30,35,40,45,50,55,60,65,70},{0.614,0.216,0.077,0.042,0.017,0.011,0.008,0.005,0.003,0.003,0.001,0.001,0,0.002}]

Group::ORTH|Path::ORTH#2,

PREOP(SCU),Duration=[{0,2,4,6,8,10,12,14,16,18,20,22,24,26,28},{0.1654,0.2266,0.1966,0.1773,0.1416,0.0686,0.0193,0.0023,0.0011,0,0.0006,0,0,0.0006}]

SUR(ORTH),Duration=[{0,0.75,1.5,2.25,3,3.375,4.5,5.25,6,6.75,7.5,8.25,9},{0.11,0.26,0.23,0.17,0.1,0.06,0.03,0.02,0,0,0,0.02}]

IC(ICU),Duration=[{0,2,4,6,8,10,12,14,16,18,20,22,24,26,28},{0.64,0.09,0.045,0.045,0.045,0.034,0.022,0.011,0.022,0.011,0,0.011,0,0.022}]

POSTOP(ORTH),Duration=[{0,5,10,15,20,25,30,35,40,45,50,55,60,65,70},{0.614,0.216,0.077,0.042,0.017,0.011,0.008,0.005,0.003,0.003,0.001,0.001,0,0.002}]

Group::ORTH|Path::ORTH#3,

PREOP(SCU),Duration=[{0,2,4,6,8,10,12,14,16,18,20,22,24,26,28},{0.1654,0.2266,0.1966,0.1773,0.1416,0.0686,0.0193,0.0023,0.0011,0,0.0006,0,0,0.0006}]

SUR(ORTH),Duration=[{0,0.75,1.5,2.25,3,3.375,4.5,5.25,6,6.75,7.5},{0.12,0.36,0.2,0.13,0.08,0.04,0.03,0.02,0.01,0.01}]

PAC(SCU),Duration=[{0,2,4,6,8,10,12,14,16,18,20},{0.04,0.23,0.36,0.21,0.08,0.03,0.01,0.01,0.01,0.02}]

POSTOP(ORTH),Duration=[{0,5,10,15,20,25,30,35,40,45,50,55,60,65,70},{0.614,0.216,0.077,0.042,0.017,0.011,0.008,0.005,0.003,0.003,0.001,0.001,0,0.002}]

Group::ORTH|Path::ORTH#4,

PREOP(SCU),Duration=[{0,2,4,6,8,10,12,14,16,18,20,22,24,26,28},{0.1654,0.2266,0.1966,0.1773,0.1416,0.0686,0.0193,0.0023,0.0011,0,0.0006,0,0,0.0006}]

SUR(ORTH),Duration=[{0,0.75,1.5,2.25,3,3.375,4.5,5.25,6,6.75,7.5},{0.12,0.36,0.2,0.13,0.08,0.04,0.03,0.02,0.01,0.01}]

IC(ICU),Duration=[{0,2,4,6,8,10,12,14,16,18,20,22,24,26,28},{0.64,0.09,0.045,0.045,0.045,0.034,0.022,0.011,0.022,0.011,0,0.011,0,0.022}]

POSTOP(ORTH),Duration=[{0,5,10,15,20,25,30,35,40,45,50,55,60,65,70},{0.614,0.216,0.077,0.042,0.017,0.011,0.008,0.005,0.003,0.003,0.001,0.001,0,0.002}]

Group::PLAS|Path::PLAS#1,

PREOP(SCU),Duration=[{0,2,4,6,8,10,12,14},{0.067,0.248,0.337,0.213,0.098,0.031,0.006}]

SUR(PLAS),Duration=[{0,0.5,1,1.5,2,2.5,3,3.5,4,4.5,5,5.5,6,12},{0.11,0.39,0.25,0.1,0.05,0.03,0.02,0.01,0.01,0,0,0,0.03}]

PAC(SCU),Duration=[{0,2,4,6,8,10,12,14,16},{0.047,0.36,0.381,0.127,0.06,0.018,0.005,0.003}]

POSTOP(PLAS),Duration=[{0,5,10,15,20,25,30,35,40,45,50,55,60,65},{0.744,0.022,0.09,0.049,0.03,0.015,0.015,0.011,0.011,0.004,0.006,0.002,0.001}]

Group::PLAS|Path::PLAS#2,

PREOP(SCU),Duration=[{0,2,4,6,8,10,12,14},{0.067,0.248,0.337,0.213,0.098,0.031,0.006}]

SUR(PLAS),Duration=[{0,0.5,1,1.5,2,2.5,3,3.5,4,4.5,5,5.5,6,12},{0.11,0.39,0.25,0.1,0.05,0.03,0.02,0.01,0.01,0,0,0,0.03}]

IC(ICU),Duration=[{0,1,2,8,9},{0.692,0.231,0,0.077}]

POSTOP(PLAS),Duration=[{0,5,10,15,20,25,30,35,40,45,50,55,60,65},{0.744,0.022,0.09,0.049,0.03,0.015,0.015,0.011,0.011,0.004,0.006,0.002,0.001}]

Group::PLAS|Path::PLAS#3,

PREOP(SCU),Duration=[{0,2,4,6,8,10,12,14},{0.067,0.248,0.337,0.213,0.098,0.031,0.006}]

SUR(PLAS),Duration=[{0,0.5,1,1.5,2,2.5,3,3.5,4,4.5,5},{0.01,0.28,0.37,0.17,0.09,0.04,0.01,0.02,0.01,0}]

PAC(SCU),Duration=[{0,2,4,6,8,10,12,14,16},{0.047,0.36,0.381,0.127,0.06,0.018,0.005,0.003}]

POSTOP(PLAS),Duration=[{0,5,10,15,20,25,30,35,40,45,50,55,60,65},{0.744,0.022,0.09,0.049,0.03,0.015,0.015,0.011,0.011,0.004,0.006,0.002,0.001}]

Group::PLAS|Path::PLAS#4,

PREOP(SCU),Duration=[{0,2,4,6,8,10,12,14},{0.067,0.248,0.337,0.213,0.098,0.031,0.006}]

SUR(PLAS),Duration=[{0,0.5,1,1.5,2,2.5,3,3.5,4,4.5,5},{0.01,0.28,0.37,0.17,0.09,0.04,0.01,0.02,0.01,0}]

IC(ICU),Duration=[{0,1,2,8,9},{0.692,0.231,0,0.077}]

POSTOP(PLAS),Duration=[{0,5,10,15,20,25,30,35,40,45,50,55,60,65},{0.744,0.022,0.09,0.049,0.03,0.015,0.015,0.011,0.011,0.004,0.006,0.002,0.001}]

Group::RESP|Path::RESP#1,

POSTOP(RESP),Duration=[{0,3,6,9,12,15,18,21,24,27,30},{0.456,0.291,0.146,0.044,0.031,0.017,0.003,0.004,0.003,0.005}]

Group::RTPT|Path::RTPT#1,

PREOP(SCU),Duration=[{0,2,4,6,8,10,12,28,30},{0.15,0.406,0.288,0.105,0.045,0.004,0,0.002}]

SUR(RTPT),Duration=[{0,1,2,3,4,5,6,7,8},{0.293,0.39,0.209,0.064,0.03,0.008,0.002,0.004}]

PAC(SCU),Duration=[{0,1.5,3,4.5,6,7.5,9,10.5,12},{0.02,0.09,0.3,0.28,0.15,0.11,0.03,0.02}]

POSTOP(RTPT),Duration=[{0,3,6,9,12,15,18,21,24,27,30},{0.577,0.171,0.153,0.045,0.017,0.021,0.004,0.007,0.001,0.004}]

Group::RTPT|Path::RTPT#2,

PREOP(SCU),Duration=[{0,2,4,6,8,10,12,28,30},{0.15,0.406,0.288,0.105,0.045,0.004,0,0.002}]

SUR(RTPT),Duration=[{0,1,2,3,4,5,6,7,8},{0.293,0.39,0.209,0.064,0.03,0.008,0.002,0.004}]

IC(ICU),Duration=[{0,1,2,3,4,5,6,7,8,9,10},{0.333,0.333,0.133,0,0.067,0,0.067,0,0,0.067}]

POSTOP(RTPT),Duration=[{0,3,6,9,12,15,18,21,24,27,30},{0.577,0.171,0.153,0.045,0.017,0.021,0.004,0.007,0.001,0.004}]

Group::RTPT|Path::RTPT#3,

PREOP(SCU),Duration=[{0,2,4,6,8,10,12,28,30},{0.15,0.406,0.288,0.105,0.045,0.004,0,0.002}]

SUR(RTPT),Duration=[{0,1,2,3,4,5,6,7,8},{0.06,0.3,0.13,0.16,0.24,0.08,0.02,0.01}]

PAC(SCU),Duration=[{0,1.5,3,4.5,6,7.5,9,10.5,12},{0.02,0.09,0.3,0.28,0.15,0.11,0.03,0.02}]

POSTOP(RTPT),Duration=[{0,3,6,9,12,15,18,21,24,27,30},{0.577,0.171,0.153,0.045,0.017,0.021,0.004,0.007,0.001,0.004}]

Group::RTPT|Path::RTPT#4,

PREOP(SCU),Duration=[{0,2,4,6,8,10,12,28,30},{0.15,0.406,0.288,0.105,0.045,0.004,0,0.002}]

SUR(RTPT),Duration=[{0,1,2,3,4,5,6,7,8},{0.06,0.3,0.13,0.16,0.24,0.08,0.02,0.01}]

IC(ICU),Duration=[{0,1,2,3,4,5,6,7,8,9,10},{0.333,0.333,0.133,0,0.067,0,0.067,0,0,0.067}]

POSTOP(RTPT),Duration=[{0,3,6,9,12,15,18,21,24,27,30},{0.577,0.171,0.153,0.045,0.017,0.021,0.004,0.007,0.001,0.004}]

Group::TRMA|Path::TRMA#1,

SUR(TRMA),Duration=[{0,1,2,3,4,5,6,7,8,10},{0.02,0.1,0.17,0.24,0.17,0.15,0.07,0.05,0.03}]

PAC(SCU),Duration=[{0,2,4,6,8,10,12,14,16,18,20,22,24},{0.111,0.2407,0.3148,0.0556,0.037,0.0185,0.0556,0.0556,0.0556,0.037,0,0.0185}

POSTOP(TRMA),Duration=[{0,1,2,3,4,5,6,7,10,11},{0.572,0.252,0.114,0.041,0.005,0.011,0.003,0,0.002}]

Group::TRMA|Path::TRMA#2,

SUR(TRMA),Duration=[{0,1,2,3,4,5,6,7,8,10},{0.02,0.1,0.17,0.24,0.17,0.15,0.07,0.05,0.03}]

POSTOP(TRMA),Duration=[{0,2,4,6,8,10,12,14,16,18,20},{0.39,0.2,0.12,0.07,0.04,0.03,0.02,0.01,0.03,0.09}]

Group::UGI|Path::UGI#1,

PREOP(SCU),Duration=[{0,2,4,6,8,10,12,14,16,18},{0.169,0.29,0.31,0.123,0.063,0.038,0.006,0,0.001}]

SUR(UGI),Duration=[{0,1,2,3,4,5,6,7,8,9,10,11,12},{0.3,0.34,0.18,0.08,0.04,0.02,0.01,0.01,0.01,0.01,0.01,0}]

PAC(SCU),Duration=[{0,2,4,6,8,10,12,14,16},{0.04,0.24,0.44,0.19,0.07,0.01,0,0.01}]

POSTOP(UGI),Duration=[{0,3,6,9,12,15,18,21,24,27,30},{0.616,0.168,0.09,0.051,0.024,0.02,0.013,0.009,0.006,0.003}]

Group::UGI|Path::UGI#2,

PREOP(SCU),Duration=[{0,2,4,6,8,10,12,14,16,18},{0.169,0.29,0.31,0.123,0.063,0.038,0.006,0,0.001}]

SUR(UGI),Duration=[{0,1,2,3,4,5,6,7,8,9,10,11,12},{0.3,0.34,0.18,0.08,0.04,0.02,0.01,0.01,0.01,0.01,0.01,0}]

IC(ICU),Duration=[{0,1,2,3,4,5,14,15},{0.67,0.21,0.04,0.02,0.04,0,0.02}]

POSTOP(UGI),Duration=[{0,3,6,9,12,15,18,21,24,27,30},{0.616,0.168,0.09,0.051,0.024,0.02,0.013,0.009,0.006,0.003}]

Group::UGI|Path::UGI#3,

PREOP(SCU),Duration=[{0,2,4,6,8,10,12,14,16,18},{0.169,0.29,0.31,0.123,0.063,0.038,0.006,0,0.001}]

SUR(UGI),Duration=[{0,1,2,3,4,5,6,7},{0.21,0.41,0.23,0.1,0.01,0.03,0.01}]

PAC(SCU),Duration=[{0,2,4,6,8,10,12,14,16},{0.04,0.24,0.44,0.19,0.07,0.01,0,0.01}]

POSTOP(UGI),Duration=[{0,3,6,9,12,15,18,21,24,27,30},{0.616,0.168,0.09,0.051,0.024,0.02,0.013,0.009,0.006,0.003}]

Group::UGI|Path::UGI#4,

PREOP(SCU),Duration=[{0,2,4,6,8,10,12,14,16,18},{0.169,0.29,0.31,0.123,0.063,0.038,0.006,0,0.001}]

SUR(UGI),Duration=[{0,1,2,3,4,5,6,7},{0.21,0.41,0.23,0.1,0.01,0.03,0.01}]

IC(ICU),Duration=[{0,1,2,3,4,5,14,15},{0.67,0.21,0.04,0.02,0.04,0,0.02}]

POSTOP(UGI),Duration=[{0,3,6,9,12,15,18,21,24,27,30},{0.616,0.168,0.09,0.051,0.024,0.02,0.013,0.009,0.006,0.003}]

Group::UROL|Path::UROL#1,

PREOP(SCU),Duration=[{0,2,4,6,8,10,12,14},{0.195,0.306,0.234,0.172,0.069,0.021,0.003}]

SUR(UROL),Duration=[{0,1,2,3,4,5,6,7,8,9},{0.509,0.276,0.063,0.067,0.059,0.018,0.005,0.002,0.001}]

PAC(SCU),Duration=[{0,2,4,6,8,10,12,14,16},{0.07,0.388,0.272,0.187,0.057,0.01,0.004,0.012}]

POSTOP(UROL),Duration=[{0,3,6,9,12,15,18,21,24,27,30},{0.607,0.229,0.072,0.048,0.021,0.007,0.005,0.003,0.004,0.004}]

Group::UROL|Path::UROL#2,

PREOP(SCU),Duration=[{0,2,4,6,8,10,12,14},{0.195,0.306,0.234,0.172,0.069,0.021,0.003}]

SUR(UROL),Duration=[{0,1,2,3,4,5,6,7,8,9},{0.509,0.276,0.063,0.067,0.059,0.018,0.005,0.002,0.001}]

IC(ICU),Duration=[{0,1,2,3,4,5},{0.611,0.111,0.222,0,0.056}]

POSTOP(UROL),Duration=[{0,3,6,9,12,15,18,21,24,27,30},{0.607,0.229,0.072,0.048,0.021,0.007,0.005,0.003,0.004,0.004}]

Group::UROL|Path::UROL#3,

PREOP(SCU),Duration=[{0,2,4,6,8,10,12,14},{0.195,0.306,0.234,0.172,0.069,0.021,0.003}]

SUR(UROL),Duration=[{0,0.5,1,1.5,2,2.5,3,3.5,4,4.5,5,5.5},{0.041,0.548,0.276,0.065,0.028,0.005,0.014,0.005,0,0.014,0.004}]

PAC(SCU),Duration=[{0,2,4,6,8,10,12,14,16},{0.07,0.388,0.272,0.187,0.057,0.01,0.004,0.012}]

POSTOP(UROL),Duration=[{0,3,6,9,12,15,18,21,24,27,30},{0.607,0.229,0.072,0.048,0.021,0.007,0.005,0.003,0.004,0.004}]

Group::UROL|Path::UROL#4,

PREOP(SCU),Duration=[{0,2,4,6,8,10,12,14},{0.195,0.306,0.234,0.172,0.069,0.021,0.003}]

SUR(UROL),Duration=[{0,0.5,1,1.5,2,2.5,3,3.5,4,4.5,5,5.5},{0.041,0.548,0.276,0.065,0.028,0.005,0.014,0.005,0,0.014,0.004}]

IC(ICU),Duration=[{0,1,2,3,4,5},{0.611,0.111,0.222,0,0.056}]

POSTOP(UROL),Duration=[{0,3,6,9,12,15,18,21,24,27,30},{0.607,0.229,0.072,0.048,0.021,0.007,0.005,0.003,0.004,0.004}]

Group::VASC|Path::VASC#1,

PREOP(SCU),Duration=[{0,2,4,6,8,10,12},{0.29,0.36,0.15,0.12,0.06,0.02}]

SUR(VASC),Duration=[{0,1,2,3,4,5,6,7,8,9,10,11,12},{0.09,0.268,0.303,0.198,0.092,0.043,0.002,0.002,0,0,0,0.002}]

PAC(SCU),Duration=[{0,2,4,6,8,10,12,14,16,18},{0.021,0.22,0.39,0.252,0.082,0.014,0.011,0.004,0.006}]

POSTOP(VASC),Duration=[{0,3,6,9,12,15,18,21,24,27,30,33,36},{0.522,0.165,0.121,0.05,0.048,0.029,0.023,0.013,0.008,0.003,0.003,0.015}]

Group::VASC|Path::VASC#2,

PREOP(SCU),Duration=[{0,2,4,6,8,10,12},{0.29,0.36,0.15,0.12,0.06,0.02}]

SUR(VASC),Duration=[{0,1,2,3,4,5,6,7,8,9,10,11,12},{0.09,0.268,0.303,0.198,0.092,0.043,0.002,0.002,0,0,0,0.002}]

IC(ICU),Duration=[{0,1,2,3,4,5,6,7,8,9,10},{0.457,0.257,0.071,0.014,0.029,0.014,0.029,0.014,0.014,0.101}]

POSTOP(VASC),Duration=[{0,3,6,9,12,15,18,21,24,27,30,33,36},{0.522,0.165,0.121,0.05,0.048,0.029,0.023,0.013,0.008,0.003,0.003,0.015}]

Group::VASC|Path::VASC#3,

PREOP(SCU),Duration=[{0,2,4,6,8,10,12},{0.29,0.36,0.15,0.12,0.06,0.02}]

SUR(VASC),Duration=[{0,1,2,3,4,5,6,7,8,9,10,11},{0.2,0.36,0.21,0.13,0.07,0.02,0,0,0.01,0,0}]

PAC(SCU),Duration=[{0,2,4,6,8,10,12,14,16,18},{0.021,0.22,0.39,0.252,0.082,0.014,0.011,0.004,0.006}]

POSTOP(VASC),Duration=[{0,3,6,9,12,15,18,21,24,27,30,33,36},{0.522,0.165,0.121,0.05,0.048,0.029,0.023,0.013,0.008,0.003,0.003,0.015}]

Group::VASC|Path::VASC#4,

PREOP(SCU),Duration=[{0,2,4,6,8,10,12},{0.29,0.36,0.15,0.12,0.06,0.02}]

SUR(VASC),Duration=[{0,1,2,3,4,5,6,7,8,9,10,11},{0.2,0.36,0.21,0.13,0.07,0.02,0,0,0.01,0,0}]

IC(ICU),Duration=[{0,1,2,3,4,5,6,7,8,9,10},{0.457,0.257,0.071,0.014,0.029,0.014,0.029,0.014,0.014,0.101}]

POSTOP(VASC),Duration=[{0,3,6,9,12,15,18,21,24,27,30,33,36},{0.522,0.165,0.121,0.05,0.048,0.029,0.023,0.013,0.008,0.003,0.003,0.015}]

**THRESHOLD ACCEPTANCE ALGORITHM: AGGREGATED RESULTS**

| **RISK** |  |  | **CAPACITY** | | | **CPU** | | | **STEPS** | | |
| --- | --- | --- | --- | --- | --- | --- | --- | --- | --- | --- | --- |
| **LEVEL** | **#SCEN** | **SCALE** | **MIN** | **AVG** | **MAX** | **MIN** | **AVG** | **MAX** | **MIN** | **AVG** | **MAX** |
| 0.2 | 500 | 0.1 | 10932 | 10968.18 | 11011.1 | 170 | 262.6 | 648 | 27 | 28.4 | 37 |
| 0.2 | 500 | 0.2 | 10979.4 | 10998.75 | 11020.6 | 223 | 363.2 | 529 | 18 | 21.2 | 26 |
| 0.2 | 500 | 0.3 | 10921.7 | 10994.99 | 11025.3 | 188 | 487.7 | 749 | 13 | 15.4 | 20 |
| 0.2 | 500 | 0.4 | 10929.3 | 11013.25 | 11089.7 | 296 | 532.4 | 872 | 13 | 15.1 | 16 |
| 0.2 | 500 | 0.5 | 10951.9 | 11000.28 | 11054.4 | 380 | 602.5 | 878 | 14 | 14 | 14 |
| 0.2 | 500 | 0.6 | 10984.9 | 11018.12 | 11043.3 | 267 | 468.6 | 781 | 13 | 13.3 | 14 |
| 0.2 | 500 | 0.7 | 10980.6 | 11036.64 | 11071.3 | 416 | 964.4 | 1633 | 12 | 14 | 17 |
| 0.2 | 500 | 0.8 | 10958.4 | 11006.17 | 11051.7 | 301 | 1286.4 | 2618 | 10 | 16.6 | 24 |
| 0.2 | 500 | 0.9 | 10968.5 | 11040.94 | 11080 | 531 | 1167.5 | 1932 | 9 | 15 | 22 |
| 0.15 | 500 | 0.1 | 10543.9 | 10619.97 | 10659.8 | 168 | 346.2 | 507 | 26 | 33.3 | 48 |
| 0.15 | 500 | 0.2 | 10525.2 | 10615.74 | 10666.3 | 165 | 344.7 | 451 | 18 | 22 | 30 |
| 0.15 | 500 | 0.3 | 10579.9 | 10660.32 | 10687 | 198 | 571.9 | 759 | 13 | 16.2 | 20 |
| 0.15 | 500 | 0.4 | 10578.7 | 10644.37 | 10692.3 | 217 | 442.3 | 757 | 14 | 15.5 | 17 |
| 0.15 | 500 | 0.5 | 10590.3 | 10632.43 | 10674.7 | 275 | 473.3 | 967 | 14 | 14 | 14 |
| 0.15 | 500 | 0.6 | 10607.5 | 10657.27 | 10698.4 | 541 | 839.4 | 1023 | 13 | 14.1 | 15 |
| 0.15 | 500 | 0.7 | 10540 | 10643.6 | 10728.6 | 139 | 884.3 | 1466 | 10 | 14.6 | 20 |
| 0.15 | 500 | 0.8 | 10607.8 | 10665.34 | 10695 | 560 | 1231.4 | 1515 | 12 | 16.7 | 19 |
| 0.15 | 500 | 0.9 | 10556.8 | 10639.68 | 10674 | 584 | 944.3 | 1833 | 12 | 14.7 | 21 |
| 0.1 | 500 | 0.1 | 10099 | 10173.36 | 10246.5 | 155 | 405.1 | 988 | 22 | 27.1 | 48 |
| 0.1 | 500 | 0.2 | 10144.8 | 10190.9 | 10233.7 | 161 | 431.9 | 802 | 18 | 20.2 | 24 |
| 0.1 | 500 | 0.3 | 10136.8 | 10190.73 | 10250.3 | 238 | 401.2 | 652 | 15 | 17.2 | 22 |
| 0.1 | 500 | 0.4 | 10130.1 | 10176.64 | 10229.4 | 176 | 370.4 | 840 | 13 | 15.1 | 17 |
| 0.1 | 500 | 0.5 | 10158.9 | 10216.35 | 10269.1 | 349 | 546.2 | 792 | 14 | 14 | 14 |
| 0.1 | 500 | 0.6 | 10135.4 | 10201.94 | 10236.1 | 308 | 501.1 | 812 | 13 | 13.2 | 14 |
| 0.1 | 500 | 0.7 | 10161.1 | 10212.55 | 10249 | 540 | 844.9 | 1109 | 12 | 14.6 | 17 |
| 0.1 | 500 | 0.8 | 10202.2 | 10228.02 | 10263.3 | 1114 | 1510.8 | 2100 | 15 | 18.7 | 25 |
| 0.1 | 500 | 0.9 | 10197.5 | 10237.64 | 10274 | 1587 | 2150.8 | 3227 | 24 | 27.2 | 29 |
| 0.05 | 500 | 0.1 | 9475.2 | 9544.402 | 9600.62 | 122 | 468.3 | 893 | 18 | 29.9 | 41 |
| 0.05 | 500 | 0.2 | 9485.64 | 9543.566 | 9636.22 | 207 | 359.5 | 569 | 15 | 19.5 | 28 |
| 0.05 | 500 | 0.3 | 9493.94 | 9535.963 | 9566.72 | 222 | 423.3 | 848 | 12 | 14.6 | 17 |
| 0.05 | 500 | 0.4 | 9430.65 | 9538.118 | 9617.06 | 149 | 445.1 | 863 | 14 | 15.7 | 17 |
| 0.05 | 500 | 0.5 | 9517.11 | 9561.391 | 9602.94 | 373 | 792 | 1726 | 14 | 14 | 14 |
| 0.05 | 500 | 0.6 | 9514.99 | 9567.912 | 9621.83 | 327 | 723.3 | 1152 | 13 | 14.1 | 16 |
| 0.05 | 500 | 0.7 | 9553.7 | 9583.652 | 9617.33 | 500 | 816.1 | 1415 | 13 | 14.5 | 17 |
| 0.05 | 500 | 0.8 | 9483.06 | 9581.301 | 9640.81 | 188 | 1259.9 | 2614 | 8 | 16.7 | 24 |
| 0.05 | 500 | 0.9 | 9528.71 | 9585.144 | 9626.34 | 811 | 1700.5 | 2570 | 12 | 21.9 | 27 |
| 0.025 | 500 | 0.1 | 8928.77 | 8998.739 | 9052.91 | 196 | 350.8 | 554 | 19 | 27.2 | 69 |
| 0.025 | 500 | 0.2 | 8933.26 | 8989.263 | 9019.95 | 159 | 363.7 | 535 | 17 | 21.9 | 28 |
| 0.025 | 500 | 0.3 | 8916.4 | 9003.864 | 9059.82 | 266 | 547.8 | 1176 | 13 | 16 | 19 |
| 0.025 | 500 | 0.4 | 8973.74 | 9030.057 | 9112.35 | 324 | 549.8 | 891 | 13 | 14.7 | 16 |
| 0.025 | 500 | 0.5 | 8942.13 | 9014.214 | 9090.25 | 151 | 487.5 | 896 | 14 | 14 | 14 |
| 0.025 | 500 | 0.6 | 8971.21 | 9040.401 | 9103.47 | 266 | 750.8 | 1079 | 12 | 13.8 | 15 |
| 0.025 | 500 | 0.7 | 8925.78 | 9010.723 | 9059.66 | 681 | 1001.3 | 1441 | 14 | 15.8 | 20 |
| 0.025 | 500 | 0.8 | 8974.4 | 9039.562 | 9079.77 | 446 | 1014.7 | 1479 | 12 | 15.6 | 19 |
| 0.025 | 500 | 0.9 | 8964.48 | 9061.148 | 9102.27 | 530 | 1742.4 | 3759 | 9 | 23.7 | 45 |
| 0.01 | 500 | 0.1 | 8282.02 | 8504.563 | 8578.27 | 206 | 397 | 700 | 15 | 23.4 | 48 |
| 0.01 | 500 | 0.2 | 8464.72 | 8523.908 | 8555.83 | 311 | 578.1 | 895 | 12 | 13.7 | 18 |
| 0.01 | 500 | 0.3 | 8457.43 | 8541.294 | 8589.41 | 315 | 604.3 | 949 | 12 | 14.3 | 17 |
| 0.01 | 500 | 0.4 | 8469.37 | 8531.968 | 8594.83 | 391 | 588 | 898 | 14 | 14.6 | 16 |
| 0.01 | 500 | 0.5 | 8487.93 | 8538.283 | 8582.11 | 328 | 547.5 | 776 | 14 | 14 | 14 |
| 0.01 | 500 | 0.6 | 8486.94 | 8535.924 | 8577.01 | 321 | 678 | 968 | 13 | 14.6 | 16 |
| 0.01 | 500 | 0.7 | 8425.56 | 8522.435 | 8591.13 | 137 | 637.2 | 1194 | 10 | 13.4 | 17 |
| 0.01 | 500 | 0.8 | 8381.31 | 8499.087 | 8572.69 | 576 | 813.8 | 1270 | 12 | 14.1 | 19 |
| 0.01 | 500 | 0.9 | 8509.27 | 8552.383 | 8598.68 | 1038 | 1655.3 | 3878 | 15 | 21.9 | 48 |

**THRESHOLD ACCEPTANCE ALGORITHM: AGGREGATED RESULTS**

|  |  |  | **CAPACITY** | | | **CPU** | | | **STEPS** | | |
| --- | --- | --- | --- | --- | --- | --- | --- | --- | --- | --- | --- |
| **RISK** | **#SCEN** | **SCALE** | **MIN** | **AVG** | **MAX** | **MIN** | **AVG** | **MAX** | **MIN** | **AVG** | **MAX** |
| 0.2 | 1000 | 0.1 | 10941 | 10970.7 | 11002.3 | 279 | 425.2 | 699 | 26 | 28.3 | 33 |
| 0.2 | 1000 | 0.2 | 10893 | 10958.8 | 11007.2 | 252 | 415.9 | 657 | 18 | 23.1 | 30 |
| 0.2 | 1000 | 0.3 | 10945 | 10975.8 | 11019.9 | 184 | 535.8 | 805 | 13 | 16.9 | 22 |
| 0.2 | 1000 | 0.4 | 10934 | 10972.1 | 10998.3 | 358 | 632.1 | 1027 | 14 | 15.1 | 17 |
| 0.2 | 1000 | 0.5 | 10931 | 10979.5 | 11007.3 | 500 | 861.8 | 1161 | 14 | 14 | 14 |
| 0.2 | 1000 | 0.6 | 10920 | 10993.6 | 11027.9 | 394 | 664 | 922 | 13 | 13.9 | 15 |
| 0.2 | 1000 | 0.7 | 10924 | 10982.9 | 11029.4 | 432 | 870.1 | 1343 | 10 | 13.6 | 15 |
| 0.2 | 1000 | 0.8 | 10934 | 10990.1 | 11022.1 | 895 | 1914.2 | 3185 | 11 | 17.7 | 22 |
| 0.2 | 1000 | 0.9 | 10977 | 10996.5 | 11027.4 | 392 | 1375.7 | 2424 | 7 | 15.1 | 25 |
| 0.15 | 1000 | 0.1 | 10541 | 10603.2 | 10643.1 | 269 | 482.8 | 820 | 24 | 32.4 | 69 |
| 0.15 | 1000 | 0.2 | 10559 | 10598.4 | 10623.3 | 259 | 442 | 817 | 17 | 21 | 30 |
| 0.15 | 1000 | 0.3 | 10556 | 10595.8 | 10628 | 320 | 496.7 | 648 | 14 | 15.3 | 17 |
| 0.15 | 1000 | 0.4 | 10594 | 10611.7 | 10628.5 | 262 | 533.9 | 926 | 14 | 15.2 | 17 |
| 0.15 | 1000 | 0.5 | 10578 | 10608.2 | 10645.8 | 388 | 701.7 | 994 | 14 | 14 | 14 |
| 0.15 | 1000 | 0.6 | 10582 | 10605.5 | 10639.3 | 471 | 710.8 | 1160 | 13 | 13.9 | 15 |
| 0.15 | 1000 | 0.7 | 10581 | 10621.9 | 10654 | 869 | 1271.8 | 1697 | 13 | 15.6 | 19 |
| 0.15 | 1000 | 0.8 | 10579 | 10615.9 | 10645.7 | 536 | 1130.9 | 1758 | 12 | 17.4 | 24 |
| 0.15 | 1000 | 0.9 | 10562 | 10617 | 10653.6 | 940 | 1516.3 | 2682 | 12 | 16.8 | 26 |
| 0.1 | 1000 | 0.1 | 10075 | 10146.2 | 10187.3 | 172 | 481.1 | 731 | 20 | 25.9 | 43 |
| 0.1 | 1000 | 0.2 | 10097 | 10152.4 | 10185.5 | 250 | 520.5 | 775 | 16 | 19.3 | 27 |
| 0.1 | 1000 | 0.3 | 10126 | 10147.7 | 10180.1 | 318 | 501.8 | 777 | 15 | 17.5 | 20 |
| 0.1 | 1000 | 0.4 | 10093 | 10153.1 | 10198.6 | 322 | 621.2 | 1243 | 13 | 15.6 | 17 |
| 0.1 | 1000 | 0.5 | 10107 | 10158.5 | 10196.9 | 283 | 876.2 | 1171 | 14 | 14 | 14 |
| 0.1 | 1000 | 0.6 | 10134 | 10159.9 | 10188.7 | 299 | 627.6 | 1198 | 12 | 13.6 | 15 |
| 0.1 | 1000 | 0.7 | 10123 | 10169.9 | 10188.5 | 669 | 1171.5 | 1604 | 13 | 14.5 | 17 |
| 0.1 | 1000 | 0.8 | 10125 | 10165.9 | 10205.8 | 1305 | 1919.8 | 2522 | 17 | 20.9 | 26 |
| 0.1 | 1000 | 0.9 | 10138 | 10185 | 10205.3 | 2380 | 3806.5 | 5588 | 27 | 36.1 | 48 |
| 0.05 | 1000 | 0.1 | 9433 | 9480.64 | 9508.18 | 180 | 411.7 | 841 | 26 | 29.4 | 37 |
| 0.05 | 1000 | 0.2 | 9416.1 | 9485.89 | 9533.17 | 237 | 522.9 | 842 | 14 | 18.8 | 30 |
| 0.05 | 1000 | 0.3 | 9441.5 | 9501.24 | 9543.32 | 127 | 687.5 | 895 | 12 | 14.3 | 20 |
| 0.05 | 1000 | 0.4 | 9459.7 | 9491.65 | 9519.83 | 371 | 523.2 | 699 | 15 | 15.8 | 17 |
| 0.05 | 1000 | 0.5 | 9476.1 | 9508.3 | 9531.54 | 547 | 862.9 | 1206 | 14 | 14 | 14 |
| 0.05 | 1000 | 0.6 | 9481.5 | 9510.99 | 9534.55 | 442 | 866.8 | 1819 | 13 | 13.8 | 15 |
| 0.05 | 1000 | 0.7 | 9463.7 | 9501.28 | 9540.34 | 616 | 895.3 | 1362 | 12 | 14 | 17 |
| 0.05 | 1000 | 0.8 | 9475 | 9513.12 | 9551.08 | 325 | 833.7 | 1709 | 9 | 12.1 | 17 |
| 0.05 | 1000 | 0.9 | 9461.7 | 9515.17 | 9544.54 | 821 | 1416.6 | 2767 | 14 | 17.7 | 26 |
| 0.025 | 1000 | 0.1 | 8896.3 | 8943.4 | 8978.19 | 261 | 584.7 | 905 | 18 | 22.6 | 41 |
| 0.025 | 1000 | 0.2 | 8890.2 | 8948.19 | 9012.51 | 268 | 530.3 | 967 | 17 | 18.7 | 22 |
| 0.025 | 1000 | 0.3 | 8884.7 | 8965.35 | 9017.28 | 377 | 621 | 832 | 13 | 16.5 | 20 |
| 0.025 | 1000 | 0.4 | 8939.1 | 8962.42 | 8986.41 | 499 | 647.3 | 898 | 13 | 15 | 16 |
| 0.025 | 1000 | 0.5 | 8895 | 8940.84 | 8974.03 | 241 | 606.7 | 1178 | 14 | 14 | 14 |
| 0.025 | 1000 | 0.6 | 8902.7 | 8962.64 | 9017.99 | 379 | 718.1 | 1044 | 12 | 14 | 17 |
| 0.025 | 1000 | 0.7 | 8939.4 | 8973.25 | 8990.86 | 934 | 1453.7 | 2035 | 15 | 17.1 | 20 |
| 0.025 | 1000 | 0.8 | 8908.6 | 8978.44 | 9028.05 | 442 | 1522.1 | 2971 | 11 | 18.3 | 24 |
| 0.025 | 1000 | 0.9 | 8947.3 | 8985.22 | 9029.46 | 551 | 1283.8 | 2843 | 8 | 15.2 | 27 |
| 0.01 | 1000 | 0.1 | 8258.1 | 8358.16 | 8418 | 214 | 516.2 | 836 | 22 | 27.2 | 48 |
| 0.01 | 1000 | 0.2 | 8369 | 8390.44 | 8408.64 | 484 | 656.6 | 824 | 12 | 13.1 | 17 |
| 0.01 | 1000 | 0.3 | 8280.8 | 8362.78 | 8442.71 | 307 | 601.7 | 863 | 13 | 14.9 | 17 |
| 0.01 | 1000 | 0.4 | 8316 | 8378.86 | 8430.41 | 515 | 734 | 954 | 13 | 14.7 | 16 |
| 0.01 | 1000 | 0.5 | 8336 | 8380.3 | 8434 | 399 | 757.7 | 1151 | 14 | 14 | 14 |
| 0.01 | 1000 | 0.6 | 8326 | 8390.43 | 8432.43 | 531 | 1031.8 | 1467 | 13 | 14.5 | 16 |
| 0.01 | 1000 | 0.7 | 8276 | 8377.28 | 8433.15 | 207 | 822.4 | 2571 | 10 | 12.3 | 15 |
| 0.01 | 1000 | 0.8 | 8297.9 | 8391.98 | 8437.67 | 650 | 1345.1 | 2097 | 13 | 17.4 | 22 |
| 0.01 | 1000 | 0.9 | 8349.5 | 8409.52 | 8447.6 | 1590 | 2009.2 | 2579 | 20 | 22.7 | 27 |

**THRESHOLD ACCEPTANCE ALGORITHM: AGGREGATED RESULTS**

| **RISK** |  |  | **CAPACITY** | | | **CPU** | | | **STEPS** | | |
| --- | --- | --- | --- | --- | --- | --- | --- | --- | --- | --- | --- |
| **LEVEL** | **#SCEN** | **SCALE** | **MIN** | **AVG** | **MAX** | **MIN** | **AVG** | **MAX** | **MIN** | **AVG** | **MAX** |
| 0.2 | 2000 | 0.1 | 10909.5 | 10948.56 | 10976 | 518 | 743.1 | 1076 | 27 | 36.5 | 69 |
| 0.2 | 2000 | 0.2 | 10925 | 10959.76 | 10981 | 389 | 811.7 | 1023 | 18 | 19.8 | 24 |
| 0.2 | 2000 | 0.3 | 10932.5 | 10966.97 | 10992 | 444 | 801 | 1231 | 13 | 17.5 | 22 |
| 0.2 | 2000 | 0.4 | 10937.9 | 10960.55 | 10975 | 695 | 987.5 | 1399 | 14 | 14.3 | 15 |
| 0.2 | 2000 | 0.5 | 10932.9 | 10962.99 | 10996 | 914 | 1272.8 | 1801 | 14 | 14 | 14 |
| 0.2 | 2000 | 0.6 | 10949.8 | 10971.37 | 10986 | 706 | 1076.4 | 1833 | 13 | 13.6 | 14 |
| 0.2 | 2000 | 0.7 | 10951 | 10974.09 | 10994 | 923 | 1637.4 | 3466 | 13 | 14.4 | 15 |
| 0.2 | 2000 | 0.8 | 10957.7 | 10980.19 | 10997 | 681 | 1824.5 | 3457 | 11 | 18.1 | 24 |
| 0.2 | 2000 | 0.9 | 10961.9 | 10979.89 | 10992 | 646 | 1325.7 | 4618 | 7 | 11.6 | 33 |
| 0.15 | 2000 | 0.1 | 10537.1 | 10579.02 | 10614 | 517 | 665.6 | 877 | 23 | 31.7 | 48 |
| 0.15 | 2000 | 0.2 | 10563.6 | 10591.09 | 10605 | 503 | 723.9 | 957 | 17 | 18.9 | 24 |
| 0.15 | 2000 | 0.3 | 10565.7 | 10594.97 | 10614 | 570 | 943.6 | 1219 | 15 | 15.4 | 17 |
| 0.15 | 2000 | 0.4 | 10543 | 10590.16 | 10614 | 454 | 897 | 1333 | 14 | 16.1 | 17 |
| 0.15 | 2000 | 0.5 | 10566 | 10588.37 | 10602 | 662 | 1005 | 1359 | 14 | 14 | 14 |
| 0.15 | 2000 | 0.6 | 10545.2 | 10582.92 | 10608 | 620 | 1119.4 | 3289 | 13 | 13.9 | 17 |
| 0.15 | 2000 | 0.7 | 10576.7 | 10595.77 | 10624 | 1303 | 1852.9 | 2557 | 15 | 16.6 | 20 |
| 0.15 | 2000 | 0.8 | 10576.1 | 10598.7 | 10628 | 709 | 1720.2 | 2773 | 13 | 17.3 | 24 |
| 0.15 | 2000 | 0.9 | 10561.6 | 10600.59 | 10620 | 1414 | 2443.6 | 4706 | 13 | 19.8 | 34 |
| 0.1 | 2000 | 0.1 | 10038.2 | 10096.21 | 10143 | 365 | 594.1 | 933 | 20 | 38.1 | 48 |
| 0.1 | 2000 | 0.2 | 10084.7 | 10111.33 | 10151 | 305 | 767.7 | 1728 | 15 | 20 | 24 |
| 0.1 | 2000 | 0.3 | 10090.2 | 10117.31 | 10157 | 446 | 885.4 | 1590 | 15 | 17.5 | 20 |
| 0.1 | 2000 | 0.4 | 10090 | 10119.9 | 10153 | 500 | 839.5 | 1095 | 14 | 15.3 | 17 |
| 0.1 | 2000 | 0.5 | 10086 | 10125.27 | 10164 | 318 | 1107.5 | 1683 | 14 | 14 | 14 |
| 0.1 | 2000 | 0.6 | 10078.2 | 10120.94 | 10157 | 571 | 970.6 | 1656 | 13 | 13.5 | 15 |
| 0.1 | 2000 | 0.7 | 10121.6 | 10141.77 | 10172 | 933 | 1471.7 | 1920 | 13 | 14.5 | 17 |
| 0.1 | 2000 | 0.8 | 10092.1 | 10126.27 | 10157 | 1402 | 1783.8 | 2411 | 18 | 19 | 23 |
| 0.1 | 2000 | 0.9 | 10127 | 10145.69 | 10158 | 3139 | 3727.7 | 4724 | 31 | 32.7 | 35 |
| 0.05 | 2000 | 0.1 | 9408.69 | 9447.813 | 9487.2 | 368 | 665.7 | 916 | 24 | 28.1 | 42 |
| 0.05 | 2000 | 0.2 | 9398.62 | 9444.198 | 9500.6 | 366 | 626.7 | 914 | 14 | 18.1 | 24 |
| 0.05 | 2000 | 0.3 | 9421.99 | 9458.831 | 9484 | 321 | 882.5 | 1368 | 11 | 14.1 | 20 |
| 0.05 | 2000 | 0.4 | 9417.2 | 9460.69 | 9492.9 | 530 | 902.4 | 1302 | 15 | 15.4 | 16 |
| 0.05 | 2000 | 0.5 | 9427.48 | 9455.358 | 9491.3 | 777 | 1147.5 | 1582 | 14 | 14 | 14 |
| 0.05 | 2000 | 0.6 | 9445.96 | 9468.515 | 9490.3 | 1003 | 1308.2 | 1736 | 14 | 14.5 | 15 |
| 0.05 | 2000 | 0.7 | 9454.21 | 9473.362 | 9509.8 | 1060 | 1579.3 | 2236 | 13 | 15.4 | 18 |
| 0.05 | 2000 | 0.8 | 9439.47 | 9477.213 | 9508.3 | 967 | 1305.6 | 1821 | 11 | 12.9 | 16 |
| 0.05 | 2000 | 0.9 | 9422.77 | 9462.959 | 9493.5 | 1419 | 2153.2 | 3299 | 15 | 18.8 | 27 |
| 0.025 | 2000 | 0.1 | 8806.21 | 8865.558 | 8933.3 | 323 | 681.9 | 1291 | 17 | 38.7 | 48 |
| 0.025 | 2000 | 0.2 | 8875.47 | 8904.013 | 8925.6 | 666 | 885.3 | 1184 | 16 | 17.4 | 19 |
| 0.025 | 2000 | 0.3 | 8845.01 | 8902.173 | 8928.1 | 473 | 923.2 | 1188 | 13 | 14.9 | 17 |
| 0.025 | 2000 | 0.4 | 8847.74 | 8892.978 | 8920.3 | 461 | 858.3 | 1083 | 13 | 14 | 16 |
| 0.025 | 2000 | 0.5 | 8882.89 | 8906.511 | 8950.5 | 640 | 883.7 | 1429 | 14 | 14 | 14 |
| 0.025 | 2000 | 0.6 | 8893.39 | 8905.327 | 8927.9 | 669 | 952.7 | 1532 | 13 | 13.6 | 15 |
| 0.025 | 2000 | 0.7 | 8892.52 | 8918.28 | 8943.4 | 1008 | 1602.4 | 2392 | 15 | 17.3 | 21 |
| 0.025 | 2000 | 0.8 | 8862.82 | 8912.675 | 8940.7 | 1092 | 1618.8 | 2408 | 12 | 13.7 | 18 |
| 0.025 | 2000 | 0.9 | 8884.51 | 8927.116 | 8958.5 | 689 | 2060.5 | 3734 | 9 | 18 | 27 |
| 0.01 | 2000 | 0.1 | 8224.38 | 8263.968 | 8352.4 | 349 | 603.1 | 1209 | 20 | 25.4 | 36 |
| 0.01 | 2000 | 0.2 | 8256.19 | 8291.191 | 8330.8 | 573 | 795.2 | 1132 | 19 | 22.7 | 30 |
| 0.01 | 2000 | 0.3 | 8273.96 | 8300.345 | 8315.9 | 821 | 1055 | 1193 | 12 | 14 | 17 |
| 0.01 | 2000 | 0.4 | 8247.55 | 8294.314 | 8322.6 | 716 | 1023.5 | 1256 | 13 | 13.9 | 16 |
| 0.01 | 2000 | 0.5 | 8261.58 | 8301 | 8349.7 | 746 | 1042.2 | 1459 | 14 | 14 | 14 |
| 0.01 | 2000 | 0.6 | 8203.7 | 8294.839 | 8337.4 | 667 | 1139 | 1593 | 13 | 14 | 15 |
| 0.01 | 2000 | 0.7 | 8252.01 | 8305.129 | 8338.9 | 439 | 1287.3 | 3275 | 11 | 14.1 | 19 |
| 0.01 | 2000 | 0.8 | 8231.23 | 8294.9 | 8341.2 | 934 | 2288.1 | 4986 | 14 | 18.5 | 24 |
| 0.01 | 2000 | 0.9 | 8309.87 | 8329.728 | 8349.7 | 2280 | 2636.8 | 3490 | 25 | 27.3 | 30 |

**SIMULATED ANNEALING ALGORITHM: AGGREGATED RESULTS**

| **RISK** |  |  | **CAPACITY** | | | **CPU** | | | **STEPS** | | |
| --- | --- | --- | --- | --- | --- | --- | --- | --- | --- | --- | --- |
| **LEVEL** | **#SCEN** | **SCALE** | **MIN** | **AVG** | **MAX** | **MIN** | **AVG** | **MAX** | **MIN** | **AVG** | **MAX** |
| 0.2 | 500 | 0.1 | 9953.68 | 10396.94 | 11002.3 | 17 | 57.3 | 299 | 24 | 30.8 | 43 |
| 0.2 | 500 | 0.2 | 10375.1 | 10819.3 | 11004.4 | 17 | 181.3 | 478 | 18 | 22.2 | 30 |
| 0.2 | 500 | 0.3 | 10552 | 10915.79 | 11038 | 19 | 279.9 | 734 | 13 | 16.2 | 20 |
| 0.2 | 500 | 0.4 | 10441.3 | 10934.68 | 11062.1 | 190 | 407.6 | 803 | 14 | 15.5 | 17 |
| 0.2 | 500 | 0.5 | 10924.5 | 11000.28 | 11062 | 266 | 455.3 | 656 | 14 | 14 | 14 |
| 0.2 | 500 | 0.6 | 9974.96 | 10908.73 | 11049.9 | 10 | 542.2 | 903 | 12 | 13.3 | 15 |
| 0.2 | 500 | 0.7 | 9990.68 | 10848.81 | 11038.9 | 74 | 421.8 | 969 | 10 | 12.2 | 14 |
| 0.2 | 500 | 0.8 | 10769.6 | 11006.69 | 11068.1 | 316 | 1280.1 | 1757 | 11 | 16.6 | 18 |
| 0.2 | 500 | 0.9 | 11005.7 | 11049.75 | 11089.6 | 481 | 1463.7 | 4210 | 8 | 17.4 | 43 |
| 0.15 | 500 | 0.1 | 10589.4 | 10635.96 | 10656 | 196 | 343.8 | 609 | 25 | 27.4 | 32 |
| 0.15 | 500 | 0.2 | 9999.71 | 10549.38 | 10669.8 | 17 | 237.6 | 491 | 18 | 21.6 | 28 |
| 0.15 | 500 | 0.3 | 10573 | 10640.42 | 10675.4 | 263 | 434.9 | 697 | 14 | 17.1 | 20 |
| 0.15 | 500 | 0.4 | 10365.8 | 10593.24 | 10692.3 | 82 | 336.6 | 633 | 14 | 15.4 | 17 |
| 0.15 | 500 | 0.5 | 10011.6 | 10520.71 | 10694.4 | 95 | 429.1 | 961 | 14 | 14 | 14 |
| 0.15 | 500 | 0.6 | 10574.6 | 10652.72 | 10711.3 | 452 | 1150.6 | 4031 | 14 | 14.9 | 17 |
| 0.15 | 500 | 0.7 | 10623.8 | 10645.21 | 10688.6 | 483 | 773 | 1254 | 13 | 14.4 | 17 |
| 0.15 | 500 | 0.8 | 10580.4 | 10650.28 | 10709.1 | 469 | 992.9 | 1637 | 12 | 14.9 | 19 |
| 0.15 | 500 | 0.9 | 10079.9 | 10583.95 | 10674.6 | 66 | 1054.6 | 2322 | 7 | 13.9 | 27 |
| 0.1 | 500 | 0.1 | 9849.85 | 10113.17 | 10246.5 | 41 | 231.5 | 417 | 21 | 29.9 | 47 |
| 0.1 | 500 | 0.2 | 9662.62 | 10098.76 | 10230.9 | 35 | 361 | 787 | 18 | 22 | 30 |
| 0.1 | 500 | 0.3 | 9697.64 | 10094.26 | 10235.4 | 101 | 369.9 | 941 | 15 | 18.5 | 21 |
| 0.1 | 500 | 0.4 | 10119.5 | 10177.36 | 10228.4 | 196 | 501.1 | 839 | 13 | 14.6 | 17 |
| 0.1 | 500 | 0.5 | 10104.2 | 10190.35 | 10258.4 | 135 | 596.2 | 959 | 14 | 14 | 14 |
| 0.1 | 500 | 0.6 | 10119.5 | 10197.71 | 10247.8 | 216 | 576.7 | 813 | 13 | 13.2 | 14 |
| 0.1 | 500 | 0.7 | 10172.4 | 10214.22 | 10249.9 | 536 | 758.7 | 1095 | 12 | 13.5 | 15 |
| 0.1 | 500 | 0.8 | 10160.5 | 10215.23 | 10277.6 | 1061 | 1555.8 | 2373 | 16 | 19.7 | 28 |
| 0.1 | 500 | 0.9 | 10188.2 | 10234.24 | 10272.6 | 1989 | 2564.6 | 3746 | 24 | 28.8 | 43 |
| 0.05 | 500 | 0.1 | 9365.95 | 9533.178 | 9598.29 | 123 | 338.6 | 558 | 18 | 28.2 | 48 |
| 0.05 | 500 | 0.2 | 9499.61 | 9541.682 | 9586.62 | 166 | 374.9 | 720 | 15 | 19.5 | 30 |
| 0.05 | 500 | 0.3 | 9484.93 | 9527.383 | 9574.27 | 322 | 448.3 | 653 | 13 | 15 | 17 |
| 0.05 | 500 | 0.4 | 9288.61 | 9515.278 | 9578.81 | 116 | 359.6 | 681 | 14 | 15.3 | 17 |
| 0.05 | 500 | 0.5 | 9380.39 | 9541.718 | 9602.18 | 269 | 689.6 | 1667 | 14 | 14 | 14 |
| 0.05 | 500 | 0.6 | 9536.69 | 9584.297 | 9630.96 | 458 | 802.4 | 1447 | 13 | 14 | 16 |
| 0.05 | 500 | 0.7 | 9526 | 9582.133 | 9617.33 | 402 | 1045.1 | 1981 | 13 | 15.1 | 22 |
| 0.05 | 500 | 0.8 | 9550.61 | 9594.583 | 9636.17 | 332 | 1171.8 | 2010 | 12 | 16.9 | 24 |
| 0.05 | 500 | 0.9 | 9461.74 | 9582.622 | 9635.84 | 811 | 1310.2 | 2199 | 12 | 17.5 | 27 |
| 0.025 | 500 | 0.1 | 8885.51 | 8973.016 | 9073.66 | 103 | 313.9 | 562 | 19 | 26.7 | 45 |
| 0.025 | 500 | 0.2 | 8910.34 | 8988.265 | 9045.57 | 133 | 341.9 | 756 | 18 | 20.1 | 24 |
| 0.025 | 500 | 0.3 | 8951.93 | 9019.464 | 9084.48 | 278 | 463.6 | 746 | 13 | 15.5 | 20 |
| 0.025 | 500 | 0.4 | 8933.47 | 9031.568 | 9127.81 | 312 | 622.1 | 994 | 13 | 14.4 | 16 |
| 0.025 | 500 | 0.5 | 8882.89 | 8998.112 | 9062.9 | 138 | 549.7 | 885 | 14 | 14 | 14 |
| 0.025 | 500 | 0.6 | 8799.46 | 8966.268 | 9047.76 | 212 | 459.7 | 853 | 12 | 13.2 | 14 |
| 0.025 | 500 | 0.7 | 8983.83 | 9018.142 | 9075.22 | 646 | 991.2 | 1532 | 14 | 15.8 | 18 |
| 0.025 | 500 | 0.8 | 9008 | 9052.088 | 9117.85 | 807 | 1208.5 | 1570 | 13 | 16.4 | 20 |
| 0.025 | 500 | 0.9 | 8978.44 | 9036.481 | 9108.85 | 268 | 1197.2 | 4165 | 7 | 17.7 | 46 |
| 0.01 | 500 | 0.1 | 8278.25 | 8485.322 | 8578.27 | 112 | 378.4 | 631 | 16 | 26 | 37 |
| 0.01 | 500 | 0.2 | 8411.1 | 8510.037 | 8576.71 | 270 | 503.8 | 740 | 12 | 14.9 | 18 |
| 0.01 | 500 | 0.3 | 8366.26 | 8493.015 | 8584.33 | 173 | 464.8 | 839 | 12 | 14.8 | 17 |
| 0.01 | 500 | 0.4 | 8417.19 | 8499.256 | 8566.02 | 226 | 502 | 769 | 13 | 14.6 | 17 |
| 0.01 | 500 | 0.5 | 8360.32 | 8509.951 | 8580.59 | 229 | 550.6 | 1040 | 14 | 14 | 14 |
| 0.01 | 500 | 0.6 | 8463.88 | 8531.738 | 8580.09 | 450 | 897.5 | 1437 | 13 | 14.9 | 17 |
| 0.01 | 500 | 0.7 | 8470.5 | 8541.013 | 8625.7 | 178 | 642.3 | 945 | 10 | 13.8 | 15 |
| 0.01 | 500 | 0.8 | 8478.32 | 8558.975 | 8619.21 | 593 | 1290.4 | 2240 | 13 | 18.2 | 26 |
| 0.01 | 500 | 0.9 | 8512.41 | 8549.117 | 8613.81 | 769 | 1290.5 | 1681 | 16 | 20.4 | 27 |

**SIMULATED ANNEALING ALGORITHM: AGGREGATED RESULTS**

|  |  |  | **CAPACITY** | | | **CPU** | | | **STEPS** | | |
| --- | --- | --- | --- | --- | --- | --- | --- | --- | --- | --- | --- |
| **RISK** | **#SCEN** | **SCALE** | **MIN** | **AVG** | **MAX** | **MIN** | **AVG** | **MAX** | **MIN** | **AVG** | **MAX** |
| 0.2 | 1000 | 0.1 | 9994.2 | 10643.9 | 11003.9 | 33 | 231.9 | 690 | 27 | 31.4 | 43 |
| 0.2 | 1000 | 0.2 | 10893 | 10959.1 | 11007.2 | 165 | 420.6 | 1012 | 19 | 22.6 | 28 |
| 0.2 | 1000 | 0.3 | 10670 | 10919.3 | 10995.6 | 122 | 520.4 | 798 | 13 | 16.6 | 22 |
| 0.2 | 1000 | 0.4 | 10944 | 10980.8 | 11006.3 | 308 | 901.7 | 3566 | 14 | 14.5 | 16 |
| 0.2 | 1000 | 0.5 | 10969 | 10986.4 | 11024 | 598 | 862.1 | 1260 | 14 | 14 | 14 |
| 0.2 | 1000 | 0.6 | 10964 | 10995 | 11022.7 | 536 | 933.6 | 1402 | 13 | 14 | 15 |
| 0.2 | 1000 | 0.7 | 10784 | 10971.9 | 11018 | 267 | 937 | 1450 | 12 | 13.9 | 17 |
| 0.2 | 1000 | 0.8 | 10982 | 11001.3 | 11028.4 | 1295 | 1836.3 | 2312 | 18 | 21.3 | 24 |
| 0.2 | 1000 | 0.9 | 10975 | 10988 | 11010.2 | 234 | 1194.6 | 3327 | 7 | 14.4 | 27 |
| 0.15 | 1000 | 0.1 | 10297 | 10547.6 | 10654.2 | 47 | 368 | 683 | 22 | 29.5 | 60 |
| 0.15 | 1000 | 0.2 | 10394 | 10554.3 | 10637.3 | 150 | 346.3 | 648 | 18 | 21 | 26 |
| 0.15 | 1000 | 0.3 | 10540 | 10598.7 | 10631.7 | 238 | 548.2 | 954 | 15 | 16.6 | 22 |
| 0.15 | 1000 | 0.4 | 10571 | 10616.2 | 10643.5 | 348 | 683 | 931 | 14 | 15.1 | 17 |
| 0.15 | 1000 | 0.5 | 10110 | 10551.5 | 10632.9 | 88 | 459.9 | 924 | 14 | 14 | 14 |
| 0.15 | 1000 | 0.6 | 10580 | 10603.3 | 10635.8 | 462 | 821.6 | 1430 | 13 | 14.2 | 16 |
| 0.15 | 1000 | 0.7 | 10556 | 10620.2 | 10648.2 | 814 | 1355.8 | 3035 | 14 | 15.8 | 17 |
| 0.15 | 1000 | 0.8 | 10581 | 10619.8 | 10651.6 | 573 | 1401.3 | 1745 | 12 | 19.2 | 26 |
| 0.15 | 1000 | 0.9 | 10561 | 10619.9 | 10675.2 | 920 | 2105.4 | 5221 | 13 | 22.4 | 48 |
| 0.1 | 1000 | 0.1 | 9893.2 | 10104.5 | 10167.1 | 58 | 356.2 | 835 | 21 | 29.6 | 48 |
| 0.1 | 1000 | 0.2 | 9954.6 | 10112.1 | 10185.5 | 118 | 519.5 | 1925 | 17 | 21.1 | 24 |
| 0.1 | 1000 | 0.3 | 9724.1 | 10037.7 | 10180.1 | 100 | 397.2 | 1163 | 16 | 18.9 | 22 |
| 0.1 | 1000 | 0.4 | 9905.5 | 10128.9 | 10193.7 | 166 | 498.1 | 1519 | 13 | 15.1 | 16 |
| 0.1 | 1000 | 0.5 | 10110 | 10145.6 | 10179.4 | 153 | 703.9 | 1057 | 14 | 14 | 14 |
| 0.1 | 1000 | 0.6 | 10103 | 10150.8 | 10180.6 | 302 | 661.5 | 993 | 13 | 13.7 | 15 |
| 0.1 | 1000 | 0.7 | 10083 | 10163.9 | 10194.8 | 548 | 822.8 | 1260 | 13 | 14 | 16 |
| 0.1 | 1000 | 0.8 | 10092 | 10147.2 | 10189.1 | 1022 | 1513.1 | 2063 | 17 | 18 | 20 |
| 0.1 | 1000 | 0.9 | 10160 | 10182.3 | 10199.2 | 2083 | 2809.6 | 3482 | 27 | 29.7 | 39 |
| 0.05 | 1000 | 0.1 | 9322.8 | 9433.77 | 9509.08 | 106 | 343.5 | 747 | 23 | 32.6 | 48 |
| 0.05 | 1000 | 0.2 | 9402.1 | 9473.94 | 9505.38 | 223 | 428.7 | 703 | 15 | 20.1 | 24 |
| 0.05 | 1000 | 0.3 | 9083.7 | 9458.48 | 9545.81 | 43 | 495.9 | 832 | 12 | 15.2 | 20 |
| 0.05 | 1000 | 0.4 | 9109.1 | 9416.67 | 9539.03 | 125 | 527.1 | 1044 | 14 | 15.5 | 17 |
| 0.05 | 1000 | 0.5 | 9466.2 | 9519.01 | 9552.05 | 683 | 1103.4 | 1295 | 14 | 14 | 14 |
| 0.05 | 1000 | 0.6 | 9412.8 | 9480.05 | 9535.41 | 439 | 638.5 | 930 | 13 | 13.7 | 15 |
| 0.05 | 1000 | 0.7 | 9336.7 | 9489.23 | 9546.15 | 339 | 920.1 | 1561 | 13 | 14 | 17 |
| 0.05 | 1000 | 0.8 | 9238.3 | 9488.04 | 9547.23 | 241 | 733 | 1317 | 9 | 11.7 | 15 |
| 0.05 | 1000 | 0.9 | 9458.2 | 9523.15 | 9557.55 | 764 | 1795.2 | 3170 | 14 | 21.4 | 36 |
| 0.025 | 1000 | 0.1 | 8742 | 8921.89 | 8996.51 | 120 | 374.7 | 645 | 17 | 25.3 | 48 |
| 0.025 | 1000 | 0.2 | 8902.7 | 9163.02 | 9366.1 | 126 | 218.4 | 307 | 13 | 13.7 | 15 |
| 0.025 | 1000 | 0.3 | 8894.5 | 8945.37 | 9017.28 | 251 | 554.4 | 974 | 13 | 16.8 | 20 |
| 0.025 | 1000 | 0.4 | 8889.2 | 8957.93 | 8996.1 | 384 | 703.4 | 1842 | 13 | 14.4 | 16 |
| 0.025 | 1000 | 0.5 | 8554.8 | 8906.51 | 8974.79 | 104 | 500 | 832 | 14 | 14 | 14 |
| 0.025 | 1000 | 0.6 | 8893.4 | 8946.89 | 8987.99 | 361 | 825.9 | 1399 | 13 | 14 | 16 |
| 0.025 | 1000 | 0.7 | 8919.3 | 8968.39 | 9001.33 | 650 | 1295.4 | 2287 | 15 | 16.6 | 20 |
| 0.025 | 1000 | 0.8 | 8616.7 | 8928.41 | 9005.75 | 305 | 1123.5 | 2621 | 11 | 15.8 | 24 |
| 0.025 | 1000 | 0.9 | 8898.2 | 8972.82 | 9022.86 | 345 | 972.5 | 2422 | 9 | 16 | 33 |
| 0.01 | 1000 | 0.1 | 8253.6 | 8314.59 | 8388.07 | 214 | 291.3 | 446 | 22 | 29.5 | 48 |
| 0.01 | 1000 | 0.2 | 8371.4 | 8407.27 | 8429.51 | 309 | 550.8 | 817 | 12 | 13.7 | 16 |
| 0.01 | 1000 | 0.3 | 8335.9 | 8384.59 | 8440.22 | 346 | 545 | 856 | 12 | 13.5 | 15 |
| 0.01 | 1000 | 0.4 | 8313.8 | 8391.04 | 8443.64 | 468 | 641.4 | 1012 | 13 | 13.9 | 16 |
| 0.01 | 1000 | 0.5 | 8336 | 8388.65 | 8415.77 | 426 | 788 | 1025 | 14 | 14 | 14 |
| 0.01 | 1000 | 0.6 | 8339.6 | 8381.11 | 8436.23 | 383 | 719.6 | 1437 | 13 | 14.4 | 16 |
| 0.01 | 1000 | 0.7 | 8310.3 | 8396.24 | 8439.2 | 253 | 614.4 | 846 | 10 | 13.2 | 15 |
| 0.01 | 1000 | 0.8 | 8348.9 | 8396.71 | 8443.64 | 821 | 1593.9 | 2979 | 14 | 18.6 | 24 |
| 0.01 | 1000 | 0.9 | 8351.1 | 8411.74 | 8466.1 | 1291 | 1637 | 2073 | 20 | 22.9 | 27 |

**SIMULATED ANNEALING ALGORITHM: AGGREGATED RESULTS**

| **RISK** |  |  | **CAPACITY** | | | **CPU** | | | **STEPS** | | |
| --- | --- | --- | --- | --- | --- | --- | --- | --- | --- | --- | --- |
| **LEVEL** | **#SCEN** | **SCALE** | **MIN** | **AVG** | **MAX** | **MIN** | **AVG** | **MAX** | **MIN** | **AVG** | **MAX** |
| 0.2 | 2000 | 0.1 | 9883.09 | 10127.05 | 10950 | 40 | 144.8 | 783 | 23 | 28 | 36 |
| 0.2 | 2000 | 0.2 | 10286.5 | 10825.16 | 10972 | 32 | 409 | 639 | 18 | 20.6 | 24 |
| 0.2 | 2000 | 0.3 | 10300.3 | 10778 | 10985 | 50 | 601.8 | 1479 | 13 | 17.6 | 22 |
| 0.2 | 2000 | 0.4 | 10951.2 | 10964.09 | 10973 | 619 | 2173.1 | 5464 | 14 | 14.6 | 15 |
| 0.2 | 2000 | 0.5 | 10923.8 | 10956.37 | 10983 | 844 | 1065 | 1332 | 14 | 14 | 14 |
| 0.2 | 2000 | 0.6 | 10942.2 | 10968.51 | 10991 | 684 | 1094.1 | 1640 | 13 | 13.9 | 16 |
| 0.2 | 2000 | 0.7 | 10951.5 | 10971.27 | 10992 | 1025 | 1465.5 | 2157 | 13 | 14.1 | 17 |
| 0.2 | 2000 | 0.8 | 10770.8 | 10963.31 | 10993 | 468 | 2073 | 2991 | 11 | 20.5 | 25 |
| 0.2 | 2000 | 0.9 | 10895.6 | 10971.93 | 10995 | 349 | 1429 | 3517 | 8 | 15 | 32 |
| 0.15 | 2000 | 0.1 | 10377.8 | 10555.46 | 10601 | 66 | 540.8 | 879 | 23 | 25.9 | 29 |
| 0.15 | 2000 | 0.2 | 10497 | 10582.81 | 10610 | 278 | 782.6 | 1294 | 17 | 19 | 24 |
| 0.15 | 2000 | 0.3 | 10537.7 | 10570.72 | 10591 | 251 | 637.3 | 1232 | 14 | 16.3 | 20 |
| 0.15 | 2000 | 0.4 | 10068.6 | 10517.46 | 10615 | 166 | 709.9 | 1291 | 14 | 15.2 | 17 |
| 0.15 | 2000 | 0.5 | 10499.2 | 10589.43 | 10615 | 227 | 1074.9 | 1464 | 14 | 14 | 14 |
| 0.15 | 2000 | 0.6 | 10582.4 | 10599.43 | 10614 | 959 | 1628.8 | 2432 | 13 | 15 | 17 |
| 0.15 | 2000 | 0.7 | 10571.2 | 10593.01 | 10607 | 1303 | 1809.6 | 2575 | 15 | 16.4 | 20 |
| 0.15 | 2000 | 0.8 | 10476.6 | 10578.47 | 10625 | 706 | 1785.6 | 3560 | 12 | 16.8 | 24 |
| 0.15 | 2000 | 0.9 | 10561.2 | 10600.38 | 10621 | 1090 | 2391.6 | 4960 | 12 | 19.6 | 36 |
| 0.1 | 2000 | 0.1 | 9718.89 | 10019.49 | 10139 | 63 | 375.7 | 878 | 20 | 33.1 | 48 |
| 0.1 | 2000 | 0.2 | 10019.6 | 10104.17 | 10146 | 215 | 513.1 | 765 | 16 | 19.6 | 26 |
| 0.1 | 2000 | 0.3 | 10044.5 | 10100 | 10139 | 235 | 509.7 | 722 | 14 | 17.8 | 22 |
| 0.1 | 2000 | 0.4 | 10046.7 | 10101.62 | 10140 | 308 | 622.6 | 1048 | 14 | 15.2 | 17 |
| 0.1 | 2000 | 0.5 | 10103.5 | 10124.14 | 10139 | 503 | 1225.3 | 1810 | 14 | 14 | 14 |
| 0.1 | 2000 | 0.6 | 10078.2 | 10115.2 | 10143 | 399 | 638.1 | 798 | 13 | 13.4 | 14 |
| 0.1 | 2000 | 0.7 | 10116.4 | 10136.91 | 10177 | 705 | 1193.2 | 2662 | 13 | 14.2 | 15 |
| 0.1 | 2000 | 0.8 | 10092.1 | 10125.54 | 10151 | 1475 | 1949.4 | 3253 | 17 | 18.7 | 24 |
| 0.1 | 2000 | 0.9 | 10121.5 | 10146.83 | 10166 | 3107 | 4045.9 | 5158 | 29 | 32.6 | 37 |
| 0.05 | 2000 | 0.1 | 9421.37 | 9449.768 | 9488.3 | 455 | 597 | 788 | 24 | 26.5 | 29 |
| 0.05 | 2000 | 0.2 | 9426.04 | 9451.848 | 9471 | 383 | 673.4 | 1314 | 17 | 18.3 | 21 |
| 0.05 | 2000 | 0.3 | 9403.93 | 9450.011 | 9485.8 | 234 | 779.1 | 1185 | 11 | 15.6 | 22 |
| 0.05 | 2000 | 0.4 | 9393.42 | 9449.43 | 9480.8 | 523 | 800.8 | 1085 | 14 | 15.3 | 16 |
| 0.05 | 2000 | 0.5 | 9414.57 | 9449.738 | 9480.7 | 647 | 1147.1 | 2919 | 14 | 14 | 14 |
| 0.05 | 2000 | 0.6 | 9350.58 | 9449.601 | 9487.9 | 526 | 882.2 | 1245 | 13 | 13.9 | 15 |
| 0.05 | 2000 | 0.7 | 9430.03 | 9468.357 | 9500 | 648 | 1384.2 | 2031 | 13 | 15 | 17 |
| 0.05 | 2000 | 0.8 | 9440.72 | 9458.684 | 9491 | 459 | 794 | 1637 | 10 | 11.1 | 13 |
| 0.05 | 2000 | 0.9 | 9423.08 | 9471.786 | 9507.9 | 1639 | 2412.1 | 3890 | 16 | 22.4 | 41 |
| 0.025 | 2000 | 0.1 | 8618.43 | 8862.908 | 8933.3 | 267 | 551.3 | 687 | 16 | 27.3 | 40 |
| 0.025 | 2000 | 0.2 | 8838.24 | 8865.345 | 8917.1 | 437 | 615 | 1135 | 16 | 20.4 | 30 |
| 0.025 | 2000 | 0.3 | 8831.63 | 8883.26 | 8925.8 | 351 | 667.4 | 1098 | 13 | 14.3 | 15 |
| 0.025 | 2000 | 0.4 | 8868.48 | 8897.913 | 8925.3 | 559 | 781.8 | 1097 | 13 | 14.4 | 16 |
| 0.025 | 2000 | 0.5 | 8840.35 | 8895.195 | 8933 | 313 | 577.5 | 784 | 14 | 14 | 14 |
| 0.025 | 2000 | 0.6 | 8875.73 | 8909.853 | 8928.7 | 529 | 1213 | 3898 | 13 | 14.1 | 16 |
| 0.025 | 2000 | 0.7 | 8854.22 | 8910.431 | 8971.5 | 888 | 1677 | 2844 | 15 | 17.4 | 20 |
| 0.025 | 2000 | 0.8 | 8779.71 | 8902.227 | 8945.9 | 686 | 1452.3 | 2186 | 11 | 14.6 | 19 |
| 0.025 | 2000 | 0.9 | 8873.85 | 8925.487 | 8953.2 | 844 | 2364.6 | 3747 | 9 | 19.6 | 27 |
| 0.01 | 2000 | 0.1 | 8147.72 | 8261.774 | 8331.9 | 307 | 747.8 | 1553 | 19 | 26.1 | 48 |
| 0.01 | 2000 | 0.2 | 7713.21 | 8190.337 | 8282.6 | 178 | 492.2 | 852 | 20 | 23.1 | 30 |
| 0.01 | 2000 | 0.3 | 8228.08 | 8285.91 | 8316.2 | 539 | 867.2 | 1245 | 11 | 14.2 | 17 |
| 0.01 | 2000 | 0.4 | 8213.28 | 8274.139 | 8322.6 | 509 | 852.4 | 1138 | 13 | 14.1 | 17 |
| 0.01 | 2000 | 0.5 | 8238.79 | 8288.315 | 8361.1 | 632 | 906.1 | 1466 | 14 | 14 | 14 |
| 0.01 | 2000 | 0.6 | 8258.82 | 8301.375 | 8331.7 | 851 | 1266.5 | 1550 | 13 | 14.2 | 16 |
| 0.01 | 2000 | 0.7 | 7926.68 | 8261.911 | 8317.9 | 108 | 919.9 | 1675 | 10 | 13.3 | 17 |
| 0.01 | 2000 | 0.8 | 8231.23 | 8299.901 | 8350.9 | 1207 | 1912.6 | 3288 | 15 | 17.9 | 24 |
| 0.01 | 2000 | 0.9 | 8244.37 | 8304.673 | 8324.1 | 2440 | 3143 | 3572 | 25 | 27 | 29 |

**THRESHOLD ACCEPTANCE. SCALE 0.1: LARGER SCENARIOS**

**CAPACITY AND CPU TIMES**

| #SCEN | REP | 0.2 | 0.15 | 0.1 | 0.05 | 0.025 | 0.01 |  | 0.2 | 0.15 | 0.1 | 0.05 | 0.025 | 0.01 |
| --- | --- | --- | --- | --- | --- | --- | --- | --- | --- | --- | --- | --- | --- | --- |
| 5000 | 0 | 10951.3 | 10576.7 | 10086.2 | 9417.39 | 8834.53 | 8231.47 |  | 1545 | 1803 | 1077 | 1020 | 1019 | 1663 |
| 5000 | 1 | 10883 | 10576.6 | 10066.8 | 9435.2 | 8850.13 | 8197.53 |  | 787 | 1242 | 577 | 1368 | 981 | 1175 |
| 5000 | 2 | 10933.3 | 10539.4 | 10103.8 | 9416.17 | 8807.42 | 8177.82 |  | 957 | 1193 | 1243 | 1074 | 678 | 1163 |
| 5000 | 3 | 10947.9 | 10517 | 10100 | 9435.31 | 8849.33 | 8191.26 |  | 923 | 1339 | 1082 | 2812 | 1075 | 638 |
| 5000 | 4 | 10947.9 | 10580.1 | 10099 | 9429.58 | 8819.55 | 8191.26 |  | 1538 | 1477 | 4054 | 1386 | 740 | 838 |
| 5000 | 5 | 10939.3 | 10551.8 | 10057 | 9397.12 | 8865.84 | 8223.75 |  | 1482 | 1601 | 1243 | 5995 | 1419 | 1857 |
| 5000 | 6 | 10909.9 | 10560.7 | 10087.5 | 9420.48 | 8857.37 | 8212.97 |  | 1482 | 1813 | 1248 | 9844 | 1150 | 1880 |
| 5000 | 7 | 10920.6 | 10538.1 | 10093.2 | 9380.06 | 8859.86 | 8226.34 |  | 1118 | 900 | 1732 | 824 | 1546 | 1790 |
| 5000 | 8 | 10960.2 | 10578.9 | 10104.2 | 9411.24 | 8849.33 | 8165.63 |  | 1770 | 1561 | 2022 | 1276 | 816 | 1050 |
| 5000 | 9 | 10948.1 | 10558.2 | 10098.6 | 9429.73 | 8858.74 | 8173.14 |  | 1797 | 1706 | 2116 | 2663 | 1477 | 1143 |
| 10000 | 0 | 10918.4 | 10558.7 | 10088.7 | 9410.74 | 8830.47 | 8164.69 |  | 3816 | 4014 | 4712 | 1834 | 1807 | 2672 |
| 10000 | 1 | 10960.2 | 10567.8 | 10091.4 | 9410.74 | 8811.4 | 8165.13 |  | 2993 | 4337 | 2815 | 1917 | 1820 | 2932 |
| 10000 | 2 | 10946.7 | 10549.5 | 10088.9 | 9365.95 | 8822.43 | 8157.6 |  | 3484 | 2329 | 4264 | 3892 | 1987 | 2713 |
| 10000 | 3 | 10930.5 | 10576.6 | 10085.7 | 9394.5 | 8821.54 | 8169.43 |  | 6737 | 2367 | 2047 | 3009 | 1652 | 3276 |
| 10000 | 4 | 10933 | 10543.8 | 10078.5 | 9429.42 | 8819.55 | 8187.75 |  | 1974 | 2337 | 3814 | 3731 | 1739 | 2214 |
| 10000 | 5 | 10946.7 | 10576.9 | 10064.6 | 9387.94 | 8830.47 | 8191.58 |  | 2866 | 2842 | 2746 | 3976 | 2218 | 3128 |
| 10000 | 6 | 10947.9 | 10537.1 | 10080.4 | 9412.6 | 8815.98 | 8144.42 |  | 2811 | 2596 | 1957 | 2811 | 3286 | 2659 |
| 10000 | 7 | 10927.2 | 10539.7 | 10078.7 | 9413.29 | 8810.51 | 8152.6 |  | 5676 | 2179 | 5135 | 2783 | 1701 | 1431 |
| 10000 | 8 | 10930.1 | 10576.6 | 10079.8 | 9411.09 | 8796.27 | 8191.26 |  | 4084 | 2474 | 1889 | 1899 | 1493 | 2015 |
| 10000 | 9 | 10934.6 | 10554.5 | 10071.8 | 9401.8 | 8830.47 | 8171.39 |  | 1675 | 2924 | 2932 | 2584 | 2146 | 3441 |
| 15000 | 0 | 10931.6 | 10547.5 | 10065.1 | 9390.19 | 8815.98 | 8160.45 |  | 3051 | 4950 | 8137 | 5488 | 12489 | 5062 |
| 15000 | 1 | 10918.4 | 10558.9 | 10081.4 | 9411.24 | 8816.32 | 8187.75 |  | 3733 | 5878 | 3137 | 4838 | 4960 | 3455 |
| 15000 | 2 | 10933.3 | 10549.3 | 10074 | 9392.56 | 8819.44 | 8159.28 |  | 4591 | 4687 | 2262 | 4048 | 2723 | 2952 |
| 15000 | 3 | 10940.1 | 10543.9 | 10067.4 | 9390.96 | 8785.53 | 8149.44 |  | 6887 | 3662 | 2374 | 4600 | 3200 | 3553 |
| 15000 | 4 | 10929.6 | 10552.6 | 10084.7 | 9411.24 | 8808.52 | 8162.82 |  | 12345 | 6688 | 5753 | 7690 | 2644 | 4712 |
| 15000 | 5 | 10913.9 | 10552.4 | 10080.9 | 9390.55 | 8807.31 | 8126.95 |  | 3785 | 4502 | 3285 | 4443 | 2900 | 3014 |
| 15000 | 6 | 10928.4 | 10556.5 | 10085.7 | 9410.74 | 8812.93 | 8156.43 |  | 5386 | 5091 | 3135 | 3978 | 3361 | 3055 |
| 15000 | 7 | 10946.6 | 10530.7 | 10062.2 | 9364.88 | 8820.54 | 8171.26 |  | 3856 | 2656 | 2776 | 5614 | 9351 | 5589 |
| 15000 | 8 | 10929.6 | 10564.7 | 10055.5 | 9395.76 | 8830.47 | 8156.43 |  | 6890 | 7126 | 2884 | 3259 | 4128 | 4594 |
| 15000 | 9 | 10947.9 | 10556.7 | 10090.9 | 9370.31 | 8819.44 | 8124.64 |  | 4083 | 7993 | 3897 | 2785 | 2876 | 3271 |
| 20000 | 0 | 10931.6 | 10554.6 | 10086.2 | 9395.61 | 8830.47 | 8129.03 |  | 3803 | 6824 | 5263 | 4008 | 4659 | 4540 |
| 20000 | 1 | 10934.6 | 10551.3 | 10093.9 | 9384.12 | 8796.16 | 8149.76 |  | 5560 | 4514 | 8639 | 4887 | 2989 | 4343 |
| 20000 | 2 | 10946.6 | 10555.1 | 10086.2 | 9397.26 | 8812.2 | 8163.18 |  | 5340 | 8774 | 4406 | 4488 | 5516 | 7583 |
| 20000 | 3 | 10946.6 | 10541 | 10075.5 | 9392.06 | 8824.61 | 8153.55 |  | 5488 | 7307 | 3848 | 3358 | 7120 | 5376 |
| 20000 | 4 | 10935.6 | 10558.5 | 10085.7 | 9389.01 | 8797.26 | 8118.72 |  | 9442 | 6298 | 3973 | 9225 | 3080 | 4567 |
| 20000 | 5 | 10931.5 | 10545.4 | 10084.3 | 9391.07 | 8831.46 | 8149.44 |  | 4949 | 5179 | 10238 | 4806 | 6461 | 3549 |
| 20000 | 6 | 10931.9 | 10556.8 | 10066.8 | 9377.96 | 8807.42 | 8150.53 |  | 7561 | 9317 | 2853 | 3475 | 4409 | 4725 |
| 20000 | 7 | 10936.7 | 10555.1 | 10070.2 | 9412.77 | 8819.55 | 8142.84 |  | 3750 | 8517 | 4355 | 6020 | 4541 | 6842 |
| 20000 | 8 | 10933.2 | 10554.3 | 10079.8 | 9398.48 | 8807.42 | 8152.95 |  | 5170 | 7429 | 3691 | 6565 | 5233 | 3850 |
| 20000 | 9 | 10946.6 | 10548.5 | 10076 | 9402.7 | 8790.45 | 8132.34 |  | 5700 | 5200 | 3846 | 6580 | 4381 | 4333 |

**Figure 2a.** Average number of search steps [#scenarios = 500, TA]

**Figure 2a.** Average number of search steps [#scenarios = 500, SA]

**Figure 2b.** Average number of search steps [#scenarios = 1000, TA]

**Figure 2b.** Average number of search steps [#scenarios = 1000, SA]

**Figure 2c.** Average number of search steps [#scenarios = 2000, TA]

**Figure 2c.** Average number of search steps [#scenarios = 2000, SA]

**Figure 3a.** Average run time [#scenarios = 500, TA]

**Figure 3a.** Average run time [#scenarios = 500, SA]

**Figure 3b.** Average run time [#scenarios = 1000, TA]

**Figure 3b.** Average run time [#scenarios = 1000, SA]

**Figure 3c.** Average run time [#scenarios = 2000, TA]

**Figure 3c.** Average run time [#scenarios = 2000, SA]
